# Discovery of biaryl macrocyclic peptides with C-terminal β-amino-α-keto acid groups

**DOI:** 10.1101/2023.10.30.564719

**Published:** 2023-11-07

**Authors:** Dinh T. Nguyen, Lingyang Zhu, Douglas A. Mitchell, Wilfred A. van der Donk

**Affiliations:** 1 Department of Chemistry, University of Illinois at Urbana-Champaign, Urbana, IL, United States; 2 Carl R. Woese Institute for Genomic Biology, University of Illinois at Urbana-Champaign, Urbana, Illinois, 61801, USA; 3 School of Chemical Sciences NMR Laboratory, University of Illinois at Urbana-Champaign, Urbana, 61801, IL, USA.

## Abstract

Advances in genome sequencing and bioinformatics methods have identified myriad biosynthetic gene clusters (BGCs) encoding uncharacterized molecules. By examining genomic databases for BGCs containing a prevalent peptide-binding domain used for the biosynthesis of approximately half of ribosomally synthesized and post-translationally modified peptides (RiPPs), we uncovered a new class involving modifications installed by a cytochrome P450, a multi-nuclear iron-dependent non-heme oxidative enzyme (MNIO, formerly DUF692), a cobalamin- and radical *S*-adenosyl-L-methionine-dependent enzyme (B12-rSAM), and a methyltransferase. The activity of each enzyme was reconstituted *in vivo* using *Burkholderia* sp. FERM BP-3421. Structural characterization demonstrated that the P450 catalyzed a biaryl C-C crosslink formation between two Tyr residues. The B12-rSAM generated β-methyltyrosine, while the MNIO transformed a C-terminal Asp residue into a C-terminal aminopyruvic acid. The methyltransferase acted on the β-carbon of the α-keto acid. Marfey’s method and exciton-coupled circular dichroism spectroscopy were used to elucidate the stereochemical configurations for the chiral carbons and the atropisomer that formed upon biaryl crosslinking. To the best of our knowledge, the MNIO featured in this biosynthetic pathway is the first to act on a non-Cys residue. Our study underscores that the pace of discovery of new macrocyclic peptides deriving from ribosomal peptides continues to accelerate and that RiPP BGCs remain a significant source of previously undiscovered enzyme chemistry.

## Introduction

Natural products are prolific sources of structurally diverse and biologically active compounds of high societal value.^1^ The rapid expansion of genomic sequence databases, which when combined with the development of high-throughput, accurate, and open-access bioinformatics tools, has unveiled many new natural product biosynthetic pathways.^2–5^ Just as advances in directed evolution revolutionized the use of enzymes in commodity chemical synthesis, enzymes sourced from natural product biosynthetic pathways have become a rich reservoir for the development of innovative biocatalytic processes.^6–9^

Ribosomally synthesized and post-translationally modified peptides (RiPPs) are a large and growing family of natural products featuring a diverse range of molecular scaffolds.^10^ The ribosomal precursor peptide frequently contains an N-terminal leader region responsible for recruiting the modifying enzymes, while a C-terminal core region receives the post-translational modifications.^11^ Exceptional transformations catalyzed by RiPP enzymes result in diverse structural moieties^10^ possessing antimicrobial, antiviral, antifungal, herbicidal, cytotoxic, anticancer, and other activities.^12–17^ With nearly 50 reported structural classes, defined by the post-translational modification(s) installed, RiPP biosynthesis is a highly productive arena for the discovery of new enzyme chemistry,^18–32^ identification of versatile bioengineering catalysts,^33–36^ and isolation of structurally exotic compounds with unprecedented biological modes of action.^37,38^

While tools like AlphaFold have provided high quality structures for millions of enzymes, a major unsolved challenge is the determination of enzyme function. Typically, one does not know the substrate for a novel enzyme of interest.^39^ However, this challenge is simplified for enzymes involved in prokaryotic RiPP biosynthesis, as the substrate(s) are typically encoded near the modifying enzyme(s) in a biosynthetic gene cluster (BGC).^10^ Advances in identifying and analyzing short open-reading frames in prokaryotic genomes has enabled reliable annotation of RiPP precursor peptides of known classes and high-confidence prediction of precursor peptides encoded by yet-uncharacterized RiPP classes.^4,40–42^

Our approach to finding BGCs that encode first-in-class RiPPs starts with bioinformatic searches centered on a prevalent, class-agnostic protein domain termed the RiPP precursor recognition element (RRE).^41,43,44^ These domains are found in the majority of prokaryotic RiPP BGCs but have been difficult to identify owing to their small size (80–90 residues), frequent fusion to much larger proteins, and high sequence variability. All known RRE domains contain three alpha helices and a three-stranded beta sheet. The cognate precursor peptides are engaged as if they were the fourth strand of the beta sheet, often with nanomolar affinities. While the structural fold of RREs is well conserved, sequence homology tools like BLAST-P are insufficient to retrieve RRE domains across RiPP classes. This diversity has necessitated the use of Hidden Markov model-(HMM) based retrieval for cataloguing RRE domains. The bioinformatic tool RRE-Finder uses a collection of HMMs and secondary structure prediction to identify these domains, which essentially serve as a biomarker of a RiPP BGC. This allows prioritization of BGCs that encode novel collections of enzymes or hypothetical enzymes with no known function. The uniqueness of the precursor peptide is also considered paramount in BGC prioritization, as it is the foundation on which the final RiPP is constructed.^4,45^ This procedure was recently followed to discover a new class of RiPPs, now termed the daptides, which modify an invariant C-terminal Thr into (*S*)-*N*_2_,*N*_2_-dimethyl-1,2-propanediamine.^41^ Thus, daptides are ribosomal peptides with two N-termini.

In this work, we pursued the structural characterization of a RiPP BGC from *Burkholderia thailandensis* E264 that is unlike any other reported. The BGC uniquely encodes three metalloenzymes that we predicted would transform the precursor peptide into an unprecedented scaffold. Indeed, we show the cumulative actions of a multinuclear non-heme iron-dependent oxidative enzyme (from protein family PF05114, abbreviated as MNIO), a cobalamin- and radical *S*-adenosyl-L-methionine-dependent enzyme, a cytochrome P450, and a methyltransferase. These enzymes installed a biaryl macrocyclic peptide formed from two Tyr residues, with one Tyr also undergoing *C*-methylation on an unactivated sp^3^ carbon center, and the resulting biaryl atropisomer-containing peptide was further decorated by the conversion of the C-terminal Asp residue to 3-amino-2-oxobutanoic acid. The MNIO is fully conserved in this pathway and is considered the class-defining enzyme. The enzyme converts the conserved C-terminal Asp residue in the precursor peptide to an aminopyruvate, and therefore we termed the products of these RiPP pathways aminopyruvatides; the BGC encoding this pathway in *B. thailandensis* was termed *apy*.

## Results

### Target prioritization.

**Figure 1.**
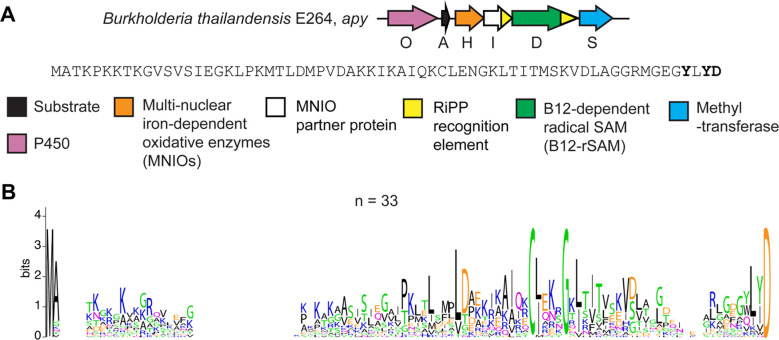
(A) BGC diagram of the RiPP pathway of interest (*apy*) from *B. thailandensis* E264, with the sequence of the precursor peptide shown. (B) Sequence logo of precursor peptides from homologous BGCs with identical sequences removed (total unique sequences = 33, total sequences = 69).

We specifically sought out an RRE-dependent RiPP BGC containing multiple uncharacterized metalloenzymes, given their unparalleled ability to catalyze difficult and diverse chemical transformations.^46–48^ The BGC that caught our interest is from *Burkholderia thailandensis* E264 and is composed of a sequence-unique substrate peptide (, NCBI accession code: ABC34935.1), an RRE-domain (ApyI), an MNIO enzyme (ApyH),^29^ methyltransferase (ApyS), cobalamin- and radical *S*-adenosyl-L-methionine-dependent enzyme (B12-rSAM, ApyD),^48,49^ and a cytochrome P450 enzyme (ApyO) (Figure 1, Supplementary Dataset 1).^47,50^ Homologous BGCs with a high level of genetic synteny and sequence conservation are prevalent in the *Burkholderia pseudomallei* group, but are also found in select strains from other classes of Pseudomonodota and even from phylogenetically distant Actinomycetota (Supplementary Dataset 1). The homologous BGCs contain a MNIO (100% occurrence), a B12-rSAM (97% occurrence), and a methyltransferase (93% occurrence), whereas only 74% of BGCs contain a P450. A sequence alignment of the predicted precursor peptides showed unique features such as a conserved C-terminal Asp and a conserved CxxxG motif in the central portion, which *a priori* could not be assigned as being part of the leader or core region (Figure 1B). Further, we noticed that two aromatic (either Trp or Tyr) residues were only present as the 2^nd^ and 4^th^ residue from the C-terminus when the BGC encoded a P450 enzyme. Identification of such correlations are often informative for determining the site(s) of enzymatic action.

### Reconstitution of enzymatic activities via heterologous expression in *E. coli.*

Initially, we constructed an *E. coli* expression plasmid encoding the precursor peptide (ApyA) containing an N-terminal hexa-His tag and all associated modifying enzymes in a pRSF-based vector (Table S1). All genes were codon-optimized for *E. coli* expression. Given the reliance of the B12-rSAM (ApyD) on cobalamin, and the inability of *E. coli* to synthesize vitamin B12 and derivatives, we co-transformed a pCDF-based vector containing the vitamin B12-uptake pathway (*btu*) and supplemented the medium with cobalamin during expression.^51,52^ The peptide products resulting from coexpression were purified using Ni-NTA affinity chromatography and digested by endoproteinase LysC or GluC, and the samples were subjected to high-resolution electrospray ionization mass spectrometry (HR-ESI-MS).

**Figure 2.**
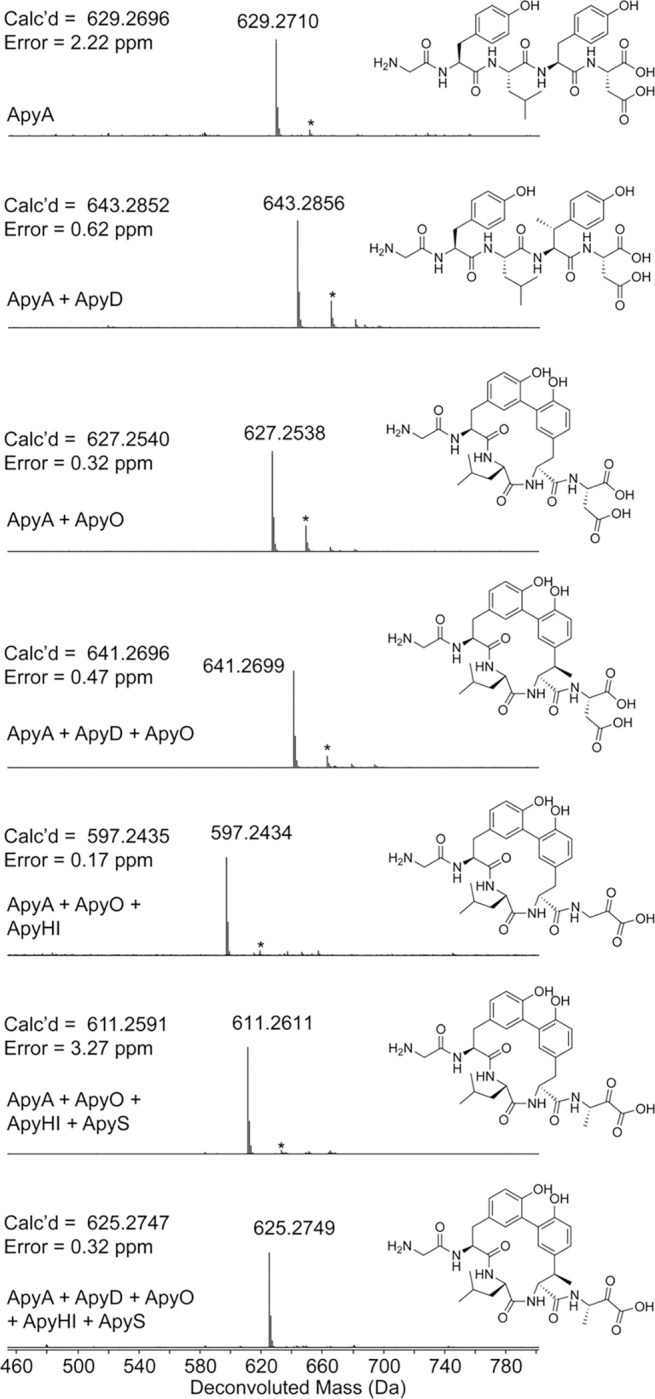
Deconvoluted HR-ESI mass spectra and elucidated structures of ApyA modified by different combinations of enzymes followed by proteolysis with GluC. Structures drawn in 2^nd^, 3^rd^, 4^th^, and 6^th^ spectra were verified by 2D-NMR. Other drawn structures were inferred from HR-ESI-MS/MS experiments and determined enzyme functions. The asterisk indicates a sodium adduct of the peptide. ApyA, precursor peptide; ApyD, B12-rSAM; ApyO, P450; ApyHI, MNIO and partner protein; ApyS, methyltransferase. The first four spectra were from expression in *E. coli* and the last three spectra were from expression in *Burkholderia* sp. FERM BP-3421. All products were purified by HPLC prior to MS analysis.

**Figure 3.**
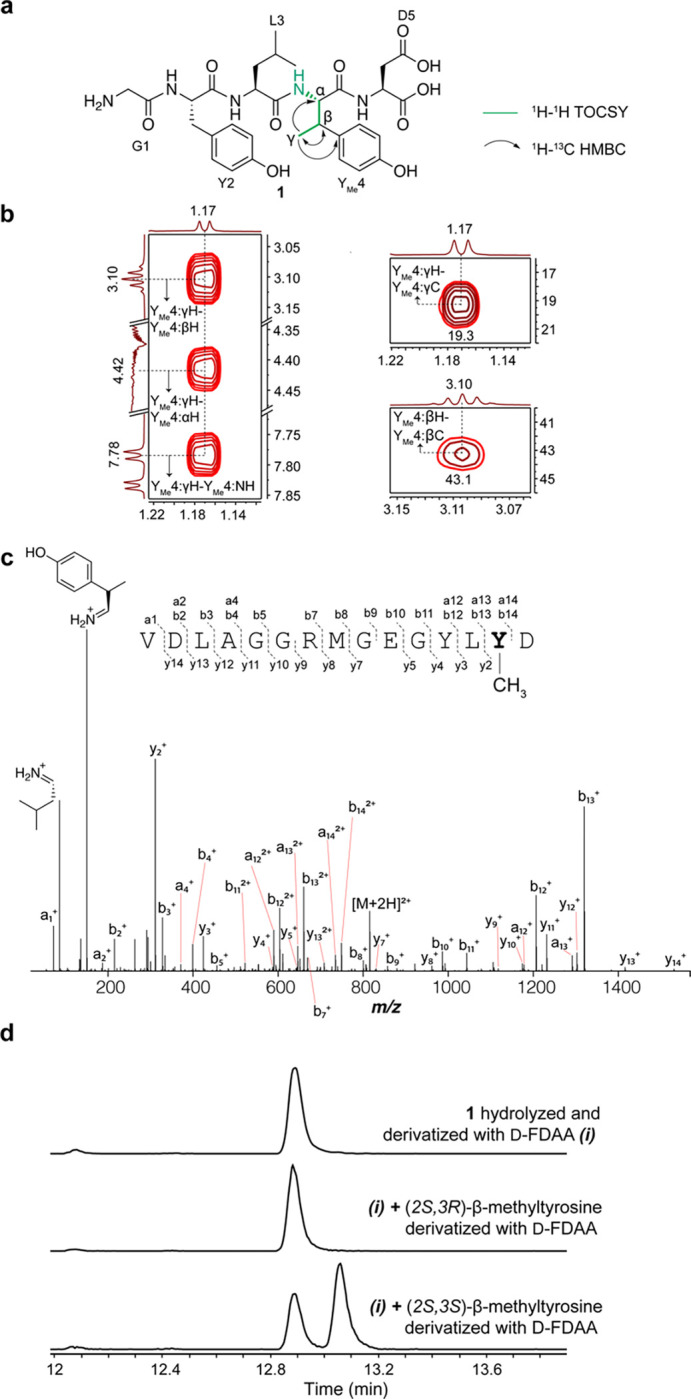
Structural elucidation of the ApyD-catalyzed modification. (A) Key NMR correlations consistent with the presence of β-methyltyrosine. (B) NMR spectral data of diagnostic cross peaks indicating the position of the new methyl group. *Left*: ^1^H-^1^H TOCSY. *Right*: ^1^H-^13^C HSQC. (C) HR-ESI-MS/MS data showing the site of methylation. (D) Extracted ion chromatograms (EICs, *m/z* = 700.1937) of **1** or authentic standards after derivatization with Marfey’s reagent (D-FDAA). The EICs of **1** and authentic standards after derivatization with L-FDAA are shown in Figure S25.

Upon coexpression of ApyA and ApyD/*btu* an increase of +14 Da was observed, suggestive of a single methylation (Figure 2, Figure S1). HR-ESI tandem mass spectrometry (HR-ESI-MS/MS) localized the 14 Da mass increase to the penultimate residue of ApyA (Tyr), which is found in a Gly-Tyr-Leu-Tyr-Asp motif (Figure 3, Figure S2). Upon coexpression of ApyA with ApyO, a mass loss of 2 Da was observed (Figure 2, Figure S1). HR-ESI-MS/MS analysis localized this change to the Tyr-Leu-Tyr region, and the lack of observable single fragmentation in this part of the peptide was suggestive of a crosslink (Figure S2). Co-expression of ApyA with ApyO and ApyD/*btu* yielded a mass change of +12 Da, consistent with the ApyO-catalyzed 2 Da mass loss and ApyD-catalyzed 14 Da mass gain (Figure S1).

Coexpression of ApyA with ApyHI (MNIO and RRE domain) in *E. coli* did not lead to any mass changes (Figure S1). Coexpression of ApyA with all protein components encoded by the BGC (ApyODHIS) yielded the same mass deviations observed upon coexpression with just ApyO and ApyD (Figure S1). Therefore, these results imply that ApyHI and ApyS were either non-functional in *E. coli* or performed mass-neutral modifications that preserved the molecular formula.

### Reconstitution of enzymatic activities via heterologous expression in *Burkholderia.*

To address the first (and more probable) hypothesis, we performed heterologous expression in *Burkholderia* sp. FERM BP-3421. This emerging chassis strain is closely related to *B. thailandensis* E264, and has been shown to significantly enhance the production of RiPP and non-RiPP natural products.^53–55^ To facilitate expression, we inserted the genomic DNA containing the BGC from *B. thailandensis* E264 downstream of the rhamnose-inducible promoter in a pSCrhaB2 plasmid used previously for protein expression in *Burkholderia* (Table S2).^56^ Additionally, we introduced an N-terminal hexa-His tag on ApyA to facilitate isolation. Upon coexpression of the entire *apy* BGC and Ni-NTA purification, the mass of the ApyA precursor peptide was decreased by 18 Da (Figure 2, Figure S3). Analysis of tandem MS data suggested that a loss of 2 Da localized to the Tyr-Leu-Tyr region identical to the ApyO-catalyzed modification observed in *E. coli* (Figure S4). These data also indicated that ApyD was inactive, as we did not observe an additional +14 Da from methylation. The remaining loss of 16 Da was confined to the conserved C-terminal Asp residue (Figure S4). Omission of ApyH or ApyI from the coexpression cultures resulted in no modification of the C-terminal Asp (Figures S5–S8). Omission of ApyS from the expression trials resulted in a 32 Da mass loss from unmodified ApyA (Figure S9). Given an inactive ApyD enzyme and 2 Da mass loss assigned to the P450 reaction, the remaining 30 Da mass loss was attributed to ApyHI. Tandem MS localized this mass deviation to the C-terminal Asp residue (Figure S10). Collectively, these data suggested that ApyHI are active upon expression in *Burkholderia*, but not *E. coli*, and work together to modify the C-terminal Asp. This finding was unexpected since all reported reactions for MNIOs conduct chemistry on Cys residues.^19,29,57^

Our initial expression trials in *Burkholderia* sp. FERM BP-3421 appeared to produce active ApyHI, ApyO, and ApyS. However, the Tyr methylation catalyzed by ApyD observed in *E. coli* was not recapitulated in *Burkholderia*. In a troubleshooting experiment, we found that expression of the pSCrhaB2 expression vector with *btu* in *E. coli* also failed to yield Tyr methylation (Figure S11). This observation indicated that the lack of ApyD activity might be attributable to a problem with the construct rather than a chassis limitation. We then redesigned the expression vector (Table S3) and introduced a separate ribosome-binding site for ApyD instead of utilizing the native gene architecture with overlapping *apyH*, *apyI*, and *apyD* genes. This construct resulted in successful Tyr methylation in *Burkholderia* sp. FERM BP-3421 and allowed isolation of a ApyA-derived product that encompassed modification by each enzyme, as supported by HR-ESI-MS/MS data (Figure 2, Figure S12–S13). This product mass loss was 4 Da compared to unmodified ApyA, which is accounted for by a putative Tyr-Tyr crosslink catalyzed by ApyO (−2 Da), methylation of the penultimate Tyr by ApyD (+14 Da), an unknown (−30 Da) modification to the C-terminal Asp, and methylation by ApyS (+14 Da).

### Structural elucidation of the ApyD and ApyO reaction products.

The MS/MS data described thus far suggested that ApyD methylates the penultimate Tyr of the ApyA peptide; however, the atomic site of methylation remained unclear. We therefore prepared a larger quantity of ApyD-modified ApyA, digested the product with endoproteinase GluC, and purified the resulting methylated Gly-Tyr-Leu-Tyr-Asp pentapeptide by high-performance liquid chromatography (HPLC). Multi-dimensional nuclear magnetic resonance (NMR) spectroscopy was then used to elucidate the structure (Figures 3, S14). A combination of ^1^H-^1^H TOCSY and ^1^H-^1^H ROESY spectra were used to assign the ^1^H chemical shifts arising from the pentapeptide (Table S4). Importantly, the ^1^H-^1^H TOCSY spectrum showed a spin system containing the typical aromatic substitution patterns of Tyr residues (AA’XX’) and correlations between N-H, C_α_-H, C_β_-H, and the new methyl group. ^1^H-^13^C HSQC analysis demonstrated the β-carbon of this Tyr to be CH instead of CH_2_, and ^1^H-^13^C HMBC produced correlations between the new methyl group, C_β_-H and C_α_-H of Tyr, and the C4 carbon of the aromatic side chain. Overall, these spectroscopic data support ApyD-catalyzed formation of β-methyltyrosine.

To ascertain the stereochemical configuration of the β-methyltyrosine product, Marfey’s method was employed.^58,59^ Specifically, the methylated pentapeptide was hydrolyzed using 6 M DCl/D_2_O, followed by derivatization with both the L- and D- forms of Marfey’s reagent (1-fluoro-2–4-dinitrophenyl-5-alanine amide) (Figure S15). The commercial synthetic standards (2*S*,3*R*)-β-methyltyrosine and (2*S*,3*S*)-β-methyltyrosine were subjected to analogous hydrolysis and derivatization conditions. Coelution in LCMS of the derivatized β-methyltyrosine obtained enzymatically with the similarly derivatized authentic standard of (2*S*,3*R*)-β-methyltyrosine (Figures 3, S15) allowed assignment of the ApyD-catalyzed product as (2*S*,3*R*)-β-methyltyrosine.

We next determined the structure of ApyO-modified ApyA utilizing a similar workflow. Integration of the ^1^H-NMR spectrum of the pentapeptide detected only six aromatic C-H protons, instead of the eight that would be expected for two unmodified Tyr residues. This finding suggested a C-C biaryl crosslink (Figure S16, Table S5). The spin system of the aromatic rings observed by ^1^H and ^1^H-^1^H TOCSY showed two groups of peaks with splitting patterns of doublet (d, 8 Hz), d of doublets (dd, 8 Hz, 2 Hz), and d (2 Hz). This change in the aromatic spin system (compared to unmodified Tyr) along with correlations between the meta-hydrogen (d, 2 Hz) of ring 1 and the ortho-carbon of ring 2 (and vice versa, demonstrated by ^1^H-^13^C HMBC), supported a crosslink between the ortho carbons of each Tyr residue. Analogous 2D-NMR experiments on the peptide resulting from coexpression of ApyA with both ApyO and ApyD were consistent with the product containing the C-C crosslink and β-methyltyrosine (Figure S17, Table S6).

We next sought to assign the stereochemistry of the atropisomer formed by the ApyO-catalyzed biaryl crosslink. We utilized exciton-coupled circular dichroism (ECD), a non-empirical method for the assignment of the spatial orientation between chromophores absorbing at similar wavelengths.^60^ We first obtained ECD spectra of four chiral synthetic molecules with a similar aromatic C-C crosslink. The ECD spectra of molecules with *R* axial chirality exhibited a negative first and positive second Cotton effect, while the ECD spectra of molecules with *S* axial chirality exhibited the opposite Cotton effect (Figure S18). Analysis of the ApyO-modified peptide yielded a negative first (307 nm) and positive second Cotton effect (285 nm), suggesting a counterclockwise orientation between the two Tyr residues, and hence we assigned *R* axial chirality (Figure S19). Control samples lacking the biaryl crosslink did not exhibit these CD signals (Figure S20).

### Structural elucidation of the reaction product from ApyO, ApyHI, and ApyS modification.

**Figure 4:**
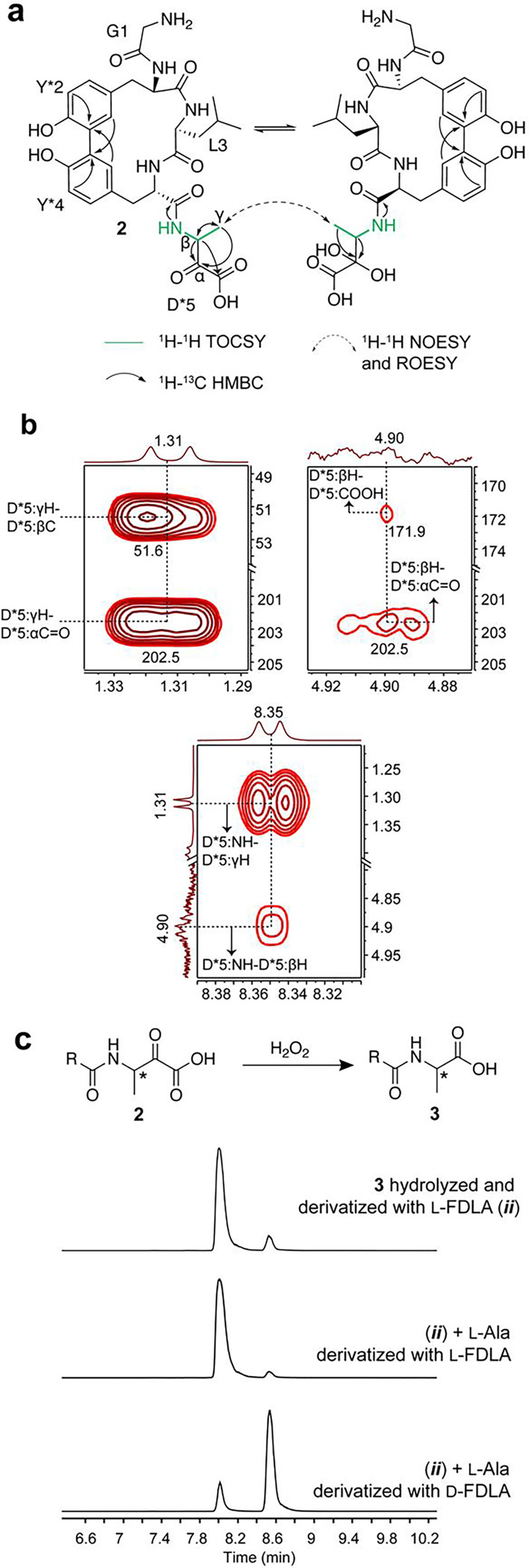
Structural elucidation of the chemistry performed by ApyO, ApyHI and ApyS. (A) Depiction of the 2D-NMR data consistent with a C-C biaryl crosslink and C-terminal 3-amino-2-oxobutanoic acid. (B) 2D-NMR spectra (panels on top: ^1^H-^13^C HMBC; panel on right: ^1^H-^1^H TOCSY) with key cross peaks elucidating the C-terminal 3-amino-2-oxobutanoic acid. (C) Schematic depiction of the oxidation of the C-terminal 3-amino-2-oxobutanoic acid by H_2_O_2_ to an Ala residue, and the EIC correspond to the *m/z* value (m/z = 382.1368) of hydrolyzed and derivatized Ala residues after Marfey’s derivatization.

We next determined the structures of the ApyA modification by ApyHI and ApyS. Omission of ApyO from the coexpression tests resulted in the absence of C-terminal Asp modification, suggesting that the biaryl crosslink is a prerequisite for ApyHI and/or ApyS activity (Figures S21–S22). Consequently, we prepared a larger quantity of ApyA modified by ApyO, ApyHI and ApyS in *Burkholderia* sp. FERM BP-3421 for structure determination. Analysis of the pentapeptide product after GluC proteolysis confirmed the C-C crosslink between the ortho carbons of the two Tyr, identical to expression in *E. coli* (Figure S23, Table S7). ^1^H-^1^H TOCSY and ^1^H-^13^C HSQC analysis revealed a new spin system for the structure originating from the C-terminal Asp that was comprised of an N-H amide (8.35 ppm), a C-H proton with a chemical shift reminiscent of α carbons of amino acid residues (4.91 ppm for ^1^H and 51.6 ppm for ^13^C), and a new methyl group (1.31 ppm). The ^1^H-^13^C HMBC spectrum revealed correlations between the carbon atoms in the C-terminal structure and all the C-H protons and a new C=O ketone based on the ^13^C chemical shift (202.5 ppm). A secondary species was detected containing an N-H amide (7.81 ppm), a C-H group (4.18 ppm for ^1^H and 50.5 ppm for ^13^C), and a methyl group (1.04 ppm) on the C-terminal residue. The ^1^H-^13^C HMBC cross peak between the methyl protons and a carbon exhibiting a chemical shift consistent with a geminal diol (95.1 ppm) suggested this secondary species represented the hydrate of the new C=O ketone, as the data were acquired in 10% D_2_O and 90% H_2_O. Consistent with this interpretation, ^1^H-^1^H NOESY and ^1^H-^1^H ROESY analysis demonstrated that the two species were in equilibrium, as both spectra exhibited a cross peak with the same phase as the diagonal peak between the 1.31 ppm and the 1.04 ppm methyl groups from each species.^61^ These results, combined with the HR-ESI-MS data showing ApyHI catalysis resulted in a loss of 30 Da from unmodified ApyA, suggested that ApyHI and ApyS converted the C-terminal Asp residue into a C-terminal 3-amino-2-oxobutanoic acid (Figure 4).

The stereochemistry of the β-carbon of the newly installed α-keto acid was determined by Marfey’s analysis. To circumvent acid-catalyzed epimerization adjacent to the ketone, we first oxidized the α-keto acid to a carboxylic acid using H_2_O_2_ (Figure 4).^62,63^ The successful conversion of the C-terminal 3-amino-2-oxobutanoic acid to a C-terminal Ala further affirmed the structural assignments. We then performed acid hydrolysis and derivatization with the advanced Marfey’s reagent 1-fluoro-2,4-dinitrophenyl-5-L-leucine-amide, and in parallel, derivatized authentic standards L- and D-Ala.^64^ LC-MS analysis indicated that the derivatized Ala post peptide hydrolysis had the L configuration, suggesting the new C-terminus installed by ApyHI and ApyS was *(S)*-3-amino-2-oxobutanoic acid.

As mentioned above, omission of ApyS from the coexpression system abolished methyl group transfer to the C-terminus of the modified peptide, implying ApyHI converted the C-terminal Asp residue into a C-terminal aminopyruvic acid moiety (Figures 2, S9–S10). Supporting this hypothesis, we made an AlphaFold^65^ model of ApyS and the closest structural homolog was MppJ, a methyltransferase converting phenylpyruvate to 3-methylphenylpyruvate (Figure S24).^66,67^ The structural and functional resemblance supports that ApyS methylates the β-carbon of the α-keto acid generated by ApyHI.

## Discussion

Using the RRE as a handle for bioinformatic identification of unique BGCs that have not been investigated previously, we prioritized a BGC from *B. thailandensis* E264. Similar BGCs are present in the genomes of other classes of Pseudomonodota and phylogenetically distant Actinomycetota. The selected BGC encodes an unprecedented combination of metalloenzymes. One of these is ApyD, a B12-dependent rSAM enzyme, which have been reported to perform methylation(s), C-S bond formation, C-P bond formation, ring contraction, and C-C cyclization.^68^ To the best of our knowledge, ApyD is the first enzyme reported to methylate the Cβ of Tyr. In contrast, the ApyO-catalyzed reaction has been observed in the biosynthesis of non-RiPP natural products arylomycin (AryC)^69^ and mycocyclosin (CYP121),^70^ albeit with different substrates and stereochemical outcomes.^71,72^ C-C bond formations involving two aromatic rings (Tyr-His; Tyr-Trp) have been reported to be catalyzed by cytochrome P450 enzymes in RiPP biosynthesis,^47^ but only the cittilins involve Tyr-Tyr coupling.^23^ Some of the precursor peptides in the identified BGCs here have other aromatic residues in the 2^nd^ and 4^th^ positions from the C-terminus (Trp/Tyr; Supplementary Dataset 1) suggesting that other biaryl couplings may be formed in their products. Since P450s are not completely conserved in the BGCs we identified, the group of natural products deriving from these clusters are not part of the biarylitides.^24,73^ Perhaps the most interesting modification we observed is catalyzed by ApyHI, which are completely conserved in all orthologous BGCs. Previously reported MNIOs catalyze distinct chemical reactions on Cys residues. Instead, ApyHI performs an unprecedented enzymatic modification on an Asp residue. We propose a mechanism for the MNIO-catalyzed reaction that first involves a four-electron oxidation of the β-carbon to form a ketone group (Figure S25), consistent with the four-electron oxidations catalyzed by all previously characterized MNIOs.^19,29,57^ The resulting β-keto acid will then decarboxylate to generate the observed C-terminal 3-amino-2-oxobutanoic acid. This structure is an oxidized analog of β-alanine, and after methylation by ApyS an analog of β-homoalanine is formed. Hence, ApyHI provides a route to β-amino acids containing a ketone functionality at the C-terminus of a ribosomally synthesized peptide, akin to the function of spliceases, rSAM enzymes that form such structures within a peptide.^18^ Further work will be necessary to substantiate the proposed enzymatic mechanism for ApyHI.

The experimental workflow in characterizing RiPP pathways often involves *E. coli* as the heterologous host. However, using *E. coli* to obtain functional enzymes from distant bacterial taxa has not been universally successful.^74–76^ We demonstrate here the successful utilization of a *Burkholderia* chassis strain to obtain active biosynthetic enzymes from a novel biosynthetic pathway, which illustrates the value of employing non-*E. coli* chassis strains for future RiPP characterization.^77–79^ This study also showcases the discovery of new enzyme functions by mining for RiPP BGCs, which contain information on both substrate(s) and biosynthetic enzymes.

In conclusion, we report the bioinformatic discovery and experimental characterization of a novel RiPP class with modifications coming from three distinct families of metalloenzymes. We successfully reconstituted the activity of each enzyme, elucidated the structures of the resulting products by HR-ESI-MS/MS and 2D-NMR, and identified the stereochemistry of the transformations. Our data suggest that the activity of ApyS is dependent on the α-keto acid moiety installed by ApyHI. Furthermore, removal of ApyO resulted in abolishment of the modifications catalyzed by ApyHI and ApyS. We thus suggest the post-translational pathway to start with the installation of the (2*S*-3*R*)-β-methyltyrosine by the B12-dependent rSAM enzyme ApyD and biaryl crosslink formation by the cytochrome P450 ApyO. These steps are apparently independent of each other. Next, the C-terminal Asp residue is converted to an aminopyruvic acid by the MNIO ApyHI. Lastly, methylation of the β-carbon of the aminopyruvic acid forms a C-terminal *(S)*-3-amino-2-oxobutanoic acid by the methyltransferase ApyS.

## Supplementary Material

Supplement 1

Supplement 2

Supplement 3
