# Supplementary material for: Discovery of biaryl macrocyclic peptides with C-terminal α-keto acid groups
Source: bioRxiv. 2023 Oct 30:2023.10.30.564719. Preprint. [Version 1] doi: 10.1101/2023.10.30.564719 (PMC10635010; doi:10.1101/2023.10.30.564719)
Supplement: Supplementary file 1 [file media-1.pdf]

SUPPORTING INFORMATION

Discovery of biaryl macrocyclic peptides with C-terminal α-keto acid groups

Dinh T. Nguyen,[1,2] Lingyang Zhu,[3] Douglas A. Mitchell[1,2,*], and Wilfred A. van der Donk[1,2,*]

[1]Department of Chemistry, [2]Carl R. Woese Institute for Genomic Biology, University of Illinois at Urbana-Champaign, Urbana, Illinois, 61801, USA, [3]School of Chemical Sciences NMR Laboratory, University of Illinois at Urbana-Champaign, Urbana, 61801, IL, USA.

* To whom correspondence should be addressed.

Supplementary Methods

Molecular cloning. All primers for polymerase chain reactions (PCR) were purchased from Integrated DNA Technologies Inc. (Coralville, IA). Genes optimized for *Escherichia coli* expressions were purchased from Twist Bioscience. Genes used for *Burkholderia.* sp FERM BP-3421 expressions were amplified from the genomic DNA of *Burkholderia thailandensis* E264, which was extracted using Qiagen gDNA Isolation Kits. The sequences of all genes are provided in Tables S1-S3.

Except for the linearized pRSF backbone generated using BamHI and XhoI (NEB), all DNA fragments were generated by PCR were performed using Q5 polymerase (NEB) following the recommended protocol of the manufacturer. Specifically, 5 cycles of 98 °C denaturation (10 s), annealing (30 s), and 72 °C extension (30 s) were first performed with annealing temperature calculated by the NEB T_m calculator. Then, 30 cycles of 98 °C denaturation (10 s), 72 °C annealing (30 s), and 72 °C extension (30 s) were performed. All DNA fragments prior to assembly were purified using agarose gel electrophoresis [0.7% (w/v)] followed by gel extraction (GeneJET).

The vectors and inserts were then ligated using Gibson Assembly Master Mix (NEB) at 50 °C for 1 h. The reaction mixtures were then used directly to transform chemically competent *E. coli* DH5α cells, which were then placed on Luria-Bertani-Miller (LB-Miller, 10 g/L Tryptone, 5 g/L Yeast, 10 g/L NaCl) agar containing 50 µg/mL kanamycin (for pRSF-based constructs) or 50 µg/mL trimethoprim (for pSCrhaB2-based constructs). All antibiotics were purchased from Gold Biotechnology Inc. (GoldBio) (St. Louis, MO). The cells were then grown at 37 °C, and random colonies were picked for an overnight culture (12-16 h, 37 °C, 220 rpm) in LB broth. Plasmids were then isolated using the GeneJET Plasmid Miniprep Kit and subjected to Sanger sequencing (UIUC Roy J. Carver Biotechnology Center) or whole plasmid sequencing (Plasmidsaurus).

Expression in *E. coli*. The pRSF constructs coexpressing BthA with modifying enzymes were used to transform chemically competent *E. coli* BL21(DE3) and placed on an LB-Miller agar plate containing 50 µg/mL kanamycin. Constructs containing *bthD* were used for cotransformation with a pCDF-based plasmid overexpressing the B12-uptake operon btu (*btuB*, *btuC*, *btuD*, *btuE*, *btuF*),[1] and placed on LB-Miller agar plate containing 50 µg/mL kanamycin and 50 µg/mL streptomycin. The plates after transformation were incubated at 37 °C for 12-15 h. For each expression, a few colonies were picked for an overnight culture (12 h, 37 °C, 220 rpm) in LB-Miller broth containing the same amount of antibiotics used in the LB-MIller plate. The cultures were then diluted at a 1:100 (v/v) ratio in Terrific Broth (24 g/L yeast extract, 12 g/L tryptone, 0.4% glycerol (v/v), 17 mM KH_2PO_4 and 72 mM K_2HPO_4) containing the same amount of antibiotics and grown at 37 °C and 200 rpm shaking.

For constructs containing *bthD*, the cultures were grown to an optical density at 600 nm (OD_{600}) of 0.6-1. At this point, 0.2 % (w/v) L-arabinose (GoldBio) and 2 µM of vitamin B12a were added (hydroxocobalamin, Sigma) to induce btu expression. The cultures then continued growing at 30 °C and 200 rpm shaking until OD 1.8-3. IPTG (0.5 mM final concentration) was then added to induce the expression of genes in the pRSF construct. Iron (II) citrate [final concetration 255 µM; stock made by mixing 100 mg/mL iron (II) ammonium sulfate heptahydrate (Sigma) and 1 M sodium citrate (Fisher Scientific)] and 200 µM L-cysteine (Sigma) were also added during IPTG

induction to support the function of the B12_rSAM BthD. The cultures then continued to grow at 18 °C and 200 rpm for 18 – 20 h before collection.

For constructs containing BthO, the cultures were grown to an OD of 1.8 - 3. IPTG (0.5 mM) was then added to induce gene expression. 5-Aminolevulinic acid (1 mM; GoldBio) was added to support the biosynthesis of heme[2] along with 255 µM iron (II) citrate. The cultures then continued to grow at 18 °C and 200 rpm for 18 – 20 h before collection.

For constructs encoding both BthD and BthO, the expression followed the same protocol as constructs containing BthD, except that 1 mM 5-aminolevulinic acid was also added during IPTG induction.

The cells were collected by centrifuging at 4500 × g for 15 min. The supernatant was then discarded, and the cell pellet was frozen and stored at -80 °C before use.

Expression in *B.* sp FERM BP-3421. A glycerol stock [20% (v/v)] of *B.* sp FERM BP-3421 fr9DEF– was revived on a Luria-Bertani-Lennox (LB-Lennox 10 g/L tryptone, 5 g/L yeast extract, 5 g/L NaCl) agar plate at 30 °C for 24 h. A single colony was picked and grown overnight at 30 °C in LB-Lennox liquid media for 24 h. The cell solution was then split into 1.5 mL Eppendorf tubes and centrifuged at 16000 × g for 2 min. The supernatant was discarded, and each tube was washed with 1 mL of 300 mM sucrose (Sigma) twice. After washing, the cells were resuspended in 100 µL of 300 mM sucrose, and 100-500 ng of pSCrhaB2 plasmid[3] was added.

The resulting solution was then put into a 0.1 cm-gap Gene Pulser Electroporation Cuvette and electroporated using a Gene Pulser Xcell electroporator (Biorad) using the following condition: C = 25 µF; PC = 200 ohm; V = 1.8 kV. Then 1 mL of LB-Lennox was added, and the cells were rescued at 30 °C for 1 h. Afterwards, the cells were centrifuged at 16000 × g for 2 min, and the supernatant was discarded until there was 100 µL of solution remaining. The cells were then resuspended, plated on an LB-Lennox Agar plate containing 120 µg/ml trimethoprim, and grown at 30 °C and 200 rpm shaking for 22-26 h.

A single colony was then grown in LB-Lennox liquid media containing 120 µg/mL trimethoprim at 30 °C and 200 rpm shaking for 22-26 h. The overnight culture was diluted at a 1:100 (v/v) ratio to 2S4G media containing: 20 g/L phytone peptone (Gibco), 40 g/L glycerol (Fisher Scientific), 2 g/L ammonium Sulfate (Sigma), 2 g/L $CaCO_3$ (Sigma), and 405 µM $MgSO_4$. The culture was then grown at 30 °C and 200 rpm shaking in a Thomson Ultra Yield 2.5 L flask (Thomson) enclosed with a vent cap containing a 0.2 µm filter. When the OD_{600} reached 0.6-1, 0.2% (w/v) of L-rhamnose (ChemImpex) was added to induce gene expression. Iron (II) citrate (255 µM), vitamin B12a (2 µM), and L-cysteine (200 µM) were also supplemented. The cells continued to grow at 30 °C and 200 rpm shaking for 24 or 42 h, with 42 h being the best option for BthD-catalyzed methylation.

The cell collection involved two steps, starting with centrifugation at 1000 × g for 2 min. The supernatant, which still contained the cells, was then carefully decanted to not carry over $CaCO_3$. After the removal of the majority of $CaCO_3$, the cells were collected by centrifuging at 7000 × g

for 20 min. The supernatant was then discarded, and the cell pellet was frozen and stored at -80 °C before use.

Cell lysis and Ni-NTA affinity purification. The cells were resuspended in 50 mL lysis buffer (6 M guanidinium hydrochloride, 50 mM phosphate, 500 mM NaCl, pH 7.5) per 10 g of estimated weight of wet cells. After resuspension, the cell solution was lysed using a high-pressure homogenizer (Avestin, Inc) at an operating pressure between 10000 and 15000 psi. The homogenization was repeated 3-4 times. The supernatant was then clarified by centrifugation at 36000 × g for 1 h. The lysate was then stirred with 1 mL of Ni-NTA resin (HisPur, Thermo) per 75 mL of lysate using a magnetic stirrer for 2 h. For the product containing the α-keto acid moiety, 5 mM of sodium pyruvate (Fisher Scientific) was added to every purification buffer. The supernatant was then removed by centrifugation at 1000 × g for 2 h followed by decantation. The Ni-NTA resin was then loaded onto a column, washed with 18 column volumes (CV) of lysis buffer (with 1 mM TCEP), 8 CV of wash buffer 1 (4 M guanidinium hydrochloride, 50 mM phosphate, 300 mM NaCl, 30 mM imidazole, pH 7.5, 1 mM TCEP), and 8 CV of wash buffer 2 (50 mM Phosphate, 1M NaCl, 30 mM imidazole, pH 7.5, 1 mM TCEP). The peptide was then eluted using 6 CV of elution buffer (50 mM phosphate, 300 mM NaCl, 1 M imidazole, pH 7.5, 1 mM TCEP).

High-performance liquid chromatography (HPLC) of full-length peptides. The eluted peptide solutions were acidified using formic acid (FA, Optima) until the pH reached approximately 3. The solutions were then injected into a Macherey Nagel-C18 HTec, 250 x 10 mm, 5 µm column, or a Phenomenex Luna-C5, 250x10mm, 10 µm column. The flow rate of the run was 4 mL/min. Solvent A was H_2O with 0.1% trifluoroacetic acid (TFA), and solvent B was CH_3CN with 0.1% TFA. Gradient: 0 – 5 min: 2% B, 5 – 35 min: 2% B to 60% B, 35 – 40 min: 60% B to 90% B, 40 min to 42 min: 90% B to 2% B. After elution, the solution was then lyophilized to dryness. Lyophilization was performed using a Labconco (Kansas City, MO) freeze dryer.

Proteolysis with endoproteinase. The HPLC-purified full peptide was dissolved in H_2O after lyophilization. The concentration of the peptide was roughly estimated using dry weight or absorbance at 280 nm using a Nanodrop. Endoproteinase GluC (Worthington) or LysC (NEB) was added to the peptide in an estimated ratio of 1:20 (w/v) for GluC and 1:100 (w/v) for LysC, and the reaction was run in digestion buffer (50 mM Tris, 1 mM TCEP, pH 8) at 37 °C for 12 h. For products containing the α-keto acid moiety, 20 µg/ml of catalase (Sigma) and 4 mM sodium pyruvate were added to the digestion reaction.

High-performance liquid chromatography (HPLC) of GluC-digested peptides.
a) BthO-modified BthA, BthD/BthO-modified BthA and BthD/BthO/BthHI/BthS-modified BthA. After digestion, the solutions were injected into a Macherey Nagel-C18 HTec, 250 x 10 mm, 5 µm column. The flow rate of the run was 4 mL/min. Solvent A was 20 mM NH_4OAc, and solvent B was CH_3CN. Gradient: 0 – 5 min: 2% B, 5 – 35 min: 2% B to 60% B, 35 – 40 min: 60% B to 90% B, 40 min to 42 min: 90% B to 2% B. After elution, the solution was lyophilized to dryness prior to downstream application (e.g., NMR analysis).

For GluC-cleaved BthD/BthO-modified BthA, prior to NMR analysis, the lyophilized product was first dissolved in a small amount of water (1 mL for products purified from 16 L of expression). The supernatant was discarded after centrifugation at 4500 × g for 15 min. The remaining pellet

was dissolved in 40% CD_3CN/60% H_2O for NMR analysis and was found to have significantly improved purity.

b) BthD-modified BthA. After digestion, the solutions were injected into a Macherey Nagel-C18 HTec, 250 x 10 mm, 5 µm column. The flow rate was 4 mL/min, and solvent A was 20 mM NH_4OAc, and solvent B was CH_3CN. Gradient: 0 – 5 min: 2% B, 5 – 35 min: 2% B to 60% B, 35 – 40 min: 60% B to 90% B, 40 min to 42 min: 90% B to 2% B. After elution, the solution was lyophilized to dryness. The dried powder was dissolved in H_2O and subjected to another round of HPLC with the same column but with solvent A as H_2O with 0.1% TFA and solvent B as CH_3CN with 0.1% TFA. After elution, the solution was lyophilized to dryness prior to downstream application (e.g., NMR analysis).

c) BthA modified with BthO, BthHI, and BthS. After digestion, the pH of the solution was adjusted to 3 using FA. The solutions were injected into a Macherey Nagel-C18 HTec, 250 x 10 mm, 5 µm column. The flow rate of the run was 4 mL/min, and solvent A was H_2O with 0.1% TFA, and solvent B was CH_3CN with 0.1% TFA. Gradient: 0 – 5 min: 2% B, 5 – 35 min: 2% B to 60% B, 35 – 40 min: 60% B to 90% B, 40 min to 42 min: 90% B to 2% B. After elution, the solution was then lyophilized to dryness. The dried powder was dissolved in H_2O and subjected to another round of HPLC with the same gradient and solvent system but with an Xbridge Prep C18 5 µm, 10x 250 mm column. The compound was found to still be contaminated with a peptide with the sequence LAGGRMGE, which resulted from GluC digestion. Thus, the mixture was treated with 250 µg of trypsin (Worthington) at 37 °C for 2 h. The resulting mixture was then subjected to another round of HPLC with the Xbridge Prep C18 column. After elution, the solution was then lyophilized to dryness prior to downstream application (e.g., NMR analysis).

LC-HR-ESI-MS/MS analysis of peptides. Samples (2-20 µL) were injected into an Agilent AdvanceBio Peptide Plus column (2.1 x 150 mm, 2.7 µm) or a Kinetex C18 LC column (2.1 x 150 mm, 2.6 µm, 100 Å) on an Agilent 6545B Q-TOF interfaced with an Agilent 1290 Infinity II LC system. Mobile phase solvent A was H_2O with 0.1% FA and solvent B was CH_3CN with 0.1% FA. The flow rate was 0.4 mL/min. The gradient was as follows: first 2-5 min 5% B, next 8 min 5% B - 70% B, last 2 min 70% B – 90% B. The column was equilibrated with 3 min of 5% B before the next samples were injected. The samples were run to waste in the first 2-5 min (depending on the time equilibrated with 5% B) before applying to the mass spectrometer. MS parameters were as follows: Mass range 100 to 1700 *m/z* ; 325 °C at 10 L/min; nebulizer, 35 psig; nozzle voltage, 0 V; sheath gas, 375 °C at 11 L/min; capillary, 3500 V; fragmentor, 120 V; skimmer, 65 V; MS scan rate (10 spectra/s); MS-MS scan rate (5 spectra/s); and isolation width (MS/MS), 1.3 *m/z*. Positive ionization mode was utilized for all MS experiments except Marfey's stereochemical analysis. Fragmentation was performed using collision-induced dissociation (CID) at 25 eV. The data analysis was performed using Agilent MassHunter Qualitative Analysis 10.0. The exact mass lists are exported and analyzed using IPSA and mMass.[4,5]

Marfey's analysis of β-methyltyrosine. GluC-cleaved and HPLC-purified methylated BthA (0.4 mg) was equilibrated under nitrogen gas and subjected to one freeze-pump-thaw cycle in a Schlenk flask. The sample was then frozen, put under vacuum, reagents were added (1 ml of 6 M DCl in D_2O (Sigma), 1% (w/v) phenol and 3% (w/v) of 2-mercaptoethanol), the tube was sealed, and equilibrated back to room temperature. The peptide was then hydrolyzed at 100 °C for 2 h. The

solvents and volatile components were removed using a SpeedVac concentrator (ThermoFisher Scientific) at 35 °C overnight. The hydrolyzed peptides were then dissolved in 150 µL of 1 M $NaHCO_3$ followed by addition of 150 µL acetone containing 4 mg/mL of L-FDAA and D-FDAA. The standards *(2S,3R)*-2-amino-3-(4-hydroxy-phenyl)-butyric acid and *(2S,3S)*-2-amino-3-(4-hydroxy-phenyl)-butyric acid (JW Pharma) were subjected to analogous hydrolysis and derivatization conditions. The reactions proceeded for 2.5 h at 60 °C, then quenched using 1 mL of 150 mM ammonium formate. The solutions were concentrated to 100 µL for LC analysis.

Aliquots (1 µL) of the samples were injected onto an Accucore PFP column (ThermoFisher Scientific) 2.6 µm, 80 Å, 150 x 4.6 column attached to an Agilent 6545B Q-TOF interfaced with an Agilent 1290 Infinity II LC system. Mobile phase solvent A was H_2O with 0.1% FA and solvent B was CH_3CN with 0.1% FA. The flow rate was 1 mL/min. The gradient was as follows: 0 - 3 min 2% B, 3 – 7 min 2% B – 43% B, 7 – 16 min 30% B – 70% B, 16 – 17 min 70% B – 90% B, 17 – 18 min 90% B – 2% B. MS parameters were as follows: Mass range 100 to 1700 *m/z* ; 200 °C at 13 L/min; nebulizer, 35 psig; nozzle voltage, 1000 V; sheath gas, 350 °C at 11 L/min; capillary, 3500 V; fragmentor, 125 V; skimmer, 65 V; MS scan rate (5 spectra/s). Negative ionization mode was utilized.

Marfey's analysis of Alanine. After GluC cleavage (without using sodium pyruvate or catalase), BthO/BthHI/BthS-modified BthA product was subjected to 200 µM H_2O_2 for 2 h at room temperature. Then 125 µM TCEP was added, and the mixture was injected into a Macherey Nagel-C18 HTec, 250 x 10 mm, 5 µm column for HPLC (with 0.1% TFA as solvent A and CH_3CN 0.1% TFA as solvent B). The flow rate of the run was 4 mL/min. After elution, the solution was lyophilized to dryness. The dried powder was dissolved in H_2O and subjected to another round of HPLC with the same gradient and solvent system but with an Xbridge Prep C18 5 µm, 10x 250 mm column.

Marfey's analysis was performed following a protocol used for lanthipeptide stereochemical analysis.[6] Specifically, after lyophilization, approximately 0.05-0.1 mg was dissolved in 0.8 mL of 6 M DCl/D_2O having 1% (w/v) phenol transferred to a PYREX 15 mL screw cap glass tube. The sample was bubbled with N_2 for 2 min, the tube was sealed, and the mixture was heated at 110 °C for 2 h. The resulting mixture was then immersed in a water bath of 80 °C and dried using a rotary evaporator, 1 mL of water was then added, and this step was repeated. After lyophilization overnight, the dried solid was dissolved in 0.6 mL of 0.8 M $NaHCO_3$ and derivatized with 0.6 mL of 3 mg/mL L-FDLA (dissolved in CH_3CN). The reaction was then stirred in the dark at 65 °C for 1.5 h. Then, 0.1 mL of 6 M HCl was slowly added to quench the reaction, and the mixture was frozen and lyophilized. The dried sample was then redissolved in 1 mL of CH_3CN and subjected to LC-MS analysis. The standards L- and D-Alanine were subjected to analogous conditions.

Aliquots of samples (1 µL) were injected onto a Kinetex 1.7 µm F5 100 Å, 100 x 2.1 mm LC column attached to an Agilent 6545B Q-TOF interfaced with an Agilent 1290 Infinity II LC system. Mobile phase solvent A was H_2O with 0.1% FA, and solvent B was CH_3CN. The flow rate was 0.4 mL/min. The gradient was as follows: 0 - 1 min 2% B, 1 – 4 min 2% B – 40% B, 4 – 13 min 40% B – 70% B, 13– 16 min 70% B – 95% B. MS parameters were as follows: Mass range 100 to 1700 *m/z* ; 200 °C at 13 L/min; nebulizer, 35 psig; nozzle voltage, 1000 V; sheath gas, 350

°C at 11 L/min; capillary, 3500 V; fragmentor, 125 V; skimmer, 65 V; MS scan rate (5 spectra/s). Negative ionization mode was utilized.

Exciton-coupled circular dichroism. Spectra were measured on a J-1500 CD system (Jasco, Inc). (*R*)-(+)- and (*S*)-(-)-5,5′,6,6′,7,7′,8,8′-octahydro-1,1′-bi-2-naphthol were purchased from TCI America. (*R*)-(+)-5,5',6,6'-Tetramethyl-3,3'-di-t-butyl-1,1'-biphenyl-2,2'-diol was purchased from VWR Strem Chemical Inc. (*S*)-(−)-5,5′,6,6′-Tetramethyl-3,3′-di-tert-butyl-1,1′-biphenyl-2,2′-diol was purchased from Sigma. The standards were dissolved in spectroscopic grade MeOH (Fisher). The BthO-modified BthA pentapeptide was dried with a SpeedVac concentrator twice from a MeOH solution before being redissolved in MeOH as a sample for circular dichroism. The estimated concentration of standards (from dry weight) and BthO-modified BthA pentapeptide (by qNMR) was 250 µM. Then 200 µL of each sample was added to a 0553Micro Rectangular Quartz Micro Cell (10 mm pathlength) for data collection. The parameters of data collection were as follows: Temperature, 23 °C; measured range 400 – 240 nm; CD scale 200 mdeg/0.1 dOD; bandwidth 1.00 nm; scanning speed 50 nm/min; data pitch 0.2 nm; digital integration time: 1 s.

NMR data acquisition and analysis. Sample information: BthD-modified BthA pentapeptide (~ 5.5 mM) in 60% H_2O and 40% CD_3CN, BthO-modified BthA pentapeptide (~ 4.4 mM) in 90% H_2O and 10% D_2O, BthO/H/I/S-modified BthA pentapeptide (total ~ 2.5 mM, containing ~ 1 mM keto form, ~ 0.5 mM enol form, and remainder are rotamers and impurities) in 90%H_2O and 10% D_2O, and BthD/O-modified BthA pentapeptide (3.0 mM) in 60% H_2O and 40% CD_3CN were dissolved in 550 µL volume and transferred into a 5-mm Wilmad 535-pp NMR tube. NMR data were collected at 25 °C on an Agilent VNMRS 750 MHz NMR spectrometer equipped with an RT indirect detection 5-mm HCN probe or a Bruker Avance NEO 600 MHz spectrometer equipped with a 5-mm BBO prodigy probe. One-dimensional (1D) ^{1}H NMR, two-dimensional (2D) homonuclear ^{1}H-^{1}H COSY (correlation spectroscopy which reveals the correlation between two neighboring protons), ^{1}H-^{1}H TOCSY (total correlation spectroscopy which reveals the correlation of protons in the same spin system) at 80 ms mixing time, ^{1}H-^{1}H NOESY (Nuclear Overhauser Effect spectroscopy which reveals close proximity in space between two protons) at 400 ms mixing time, or ^{1}H-^{1}H ROESY at 300 ms mixing time (NOESY in rotating frame, revealing the close proximity in space between two protons), ^{1}H-^{13}C HSQC (heteronuclear single quantum coherence spectroscopy, revealing one-bond correlation between ^{1}H and ^{13}C), and ^{1}H-^{13}C HMBC (heteronuclear multiple bond correlation, revealing long range ^{1}H-^{13}C connectivity such as 2-bond, 3-bond, or 4 or more bonds) spectra were acquired for each sample using either the Biopack pulse sequences in the VNMRJ 4.2A software or pulse programs in Bruker Topspin 4.1.3. The spectra were processed and analyzed in Mnova (version 14.3.0. Mestrelab Research). The sample concentrations were determined by ^{1}H spectra collected under the qNMR condition on an Agilent VNMRS 750 MHz spectrometer with a HCN probe that was calibrated with a known standard. The calibrated parameters were saved to the probe file and used to calculate the sample concentration based on the integration values of the proton peaks.

Table S1: Sequence of the pRSF-based vectors used in this study for expression in *E. coli*. Each gene is highlighted as follows. *bthA*: bolded black; *bthO*: pink; *bthD*: green; *bthH*: orange; *bthI*: black underlined, *bthS*: blue. The N-terminus of BthA contains a hexa-His tag.

a) Backbone of pRSF vector

CTCGAGTCTGGTAAAGAAACCGCTGCTGCGAAATTTGAACGCCAGCACATGGACTCGTCTACTAGCGCAGCTTAATT
AACCTAGGCTGCTGCCACCGCTGAGCAATAACTAGCATAACCCCTTGGGGCCTCTAAACGGGTCTTGAGGGGTTTTT
TGCTGAAACCTCAGGCATTTGAGAAGCACACGGTCACACTGCTTCCGGTAGTCAATAAACCGGTAAACCAGCAATAG
ACATAAGCGGCTATTTAACGACCCTGCCCTGAACCGACGACAAGCTGACGACCGGGTCTCCGCAAGTGGCACTTTTC
GGGGAAATGTGCGCGGAACCCCTATTTGTTTATTTTTCTAAATACATTCAAATATGTATCCGCTCATGAATTAATTC
TTAGAAAAACTCATCGAGCATCAAATGAAACTGCAATTTATTCATATCAGGATTATCAATACCATATTTTTGAAAAA
GCCGTTTCTGTAATGAAGGAGAAAACTCACCGAGGCAGTTCCATAGGATGGCAAGATCCTGGTATCGGTCTGCGATT
CCGACTCGTCCAACATCAATACAACCTATTAATTTCCCCTCGTCAAAAATAAGGTTATCAAGTGAGAAATCACCATG
AGTGACGACTGAATCCGGTGAGAATGGCAAAAGTTTATGCATTTCTTTCCAGACTTGTTCAACAGGCCAGCCATTAC
GCTCGTCATCAAAATCACTCGCATCAACCAAACCGTTATTCATTCGTGATTGCGCCTGAGCGAGACGAAATACGCGG
TCGCTGTTAAAAGGACAATTACAAACAGGAATCGAATGCAACCGGCGCAGGAACACTGCCAGCGCATCAACAATATT
TTCACCTGAATCAGGATATTCTTCTAATACCTGGAATGCTGTTTTCCCGGGGATCGCAGTGGTGAGTAACCATGCAT
CATCAGGAGTACGGATAAAATGCTTGATGGTCGGAAGAGGCATAAATTCCGTCAGCCAGTTTAGTCTGACCATCTCA
TCTGTAACATCATTGGCAACGCTACCTTTGCCATGTTTCAGAAACAACTCTGGCGCATCGGGCTTCCCATACAATCG
ATAGATTGTCGCACCTGATTGCCCGACATTATCGCGAGCCCATTTATACCCATATAAATCAGCATCCATGTTGGAAT
TTAATCGCGGCCTAGAGCAAGACGTTTCCCGTTGAATATGGCTCATACTCTTCCTTTTTCAATATTATTGAAGCATT
TATCAGGGTTATTGTCTCATGAGCGGATACATATTTGAATGTATTTAGAAAAATAAACAAATAGGCATGCAGCGCTC
TTCCGCTTCCTCGCTCACTGACTCGCTACGCTCGGTCGTTCGACTGCGGCGAGCGGTGTCAGCTCACTCAAAAGCGG
TAATACGGTTATCCACAGAATCAGGGGATAAAGCCGGAAAGAACATGTGAGCAAAAAGCAAAGCACCGGAAGAAGCC
AACGCCGCAGGCGTTTTTCCATAGGCTCCGCCCCCCTGACGAGCATCACAAAAATCGACGCTCAAGCCAGAGGTGGC
GAAACCCGACAGGACTATAAAGATACCAGGCGTTTCCCCCTGGAAGCTCCCTCGTGCGCTCTCCTGTTCCGACCCTG
CCGCTTACCGGATACCTGTCCGCCTTTCTCCCTTCGGGAAGCGTGGCGCTTTCTCATAGCTCACGCTGTTGGTATCT
CAGTTCGGTGTAGGTCGTTCGCTCCAAGCTGGGCTGTGTGCACGAACCCCCCGTTCAGCCCGACCGCTGCGCCTTAT
CCGGTAACTATCGTCTTGAGTCCAACCCGGTAAGACACGACTTATCGCCACTGGCAGCAGCCATTGGTAACTGATTT
AGAGGACTTTGTCTTGAAGTTATGCACCTGTTAAGGCTAAACTGAAAGAACAGATTTTGGTGAGTGCGGTCCTCCAA
CCCACTTACCTTGGTTCAAAGAGTTGGTAGCTCAGCGAACCTTGAGAAAACCACCGTTGGTAGCGGTGGTTTTTCTT
TATTTATGAGATGATGAATCAATCGGTCTATCAAGTCAACGAACAGCTATTCCGTTACTCTAGATTTCAGTGCAATT
TATCTCTTCAAATGTAGCACCTGAAGTCAGCCCCATACGATATAAGTTGTAATTCTCATGTTAGTCATGCCCCGCGC
CCACCGGAAGGAGCTGACTGGGTTGAAGGCTCTCAAGGGCATCGGTCGAGATCCCGGTGCCTAATGAGTGAGCTAAC
TTACATTAATTGCGTTGCGCTCACTGCCCGCTTTCCAGTCGGGAAACCTGTCGTGCCAGCTGCATTAATGAATCGGC
CAACGCGCGGGGAGAGGCGGTTTGCGTATTGGGCGCCAGGGTGGTTTTTCTTTTCACCAGTGAGACGGGCAACAGCT
GATTGCCCTTCACCGCCTGGCCCTGAGAGAGTTGCAGCAAGCGGTCCACGCTGGTTTGCCCCAGCAGGCGAAAATCC
TGTTTGATGGTGGTTAACGGCGGGATATAACATGAGCTGTCTTCGGTATCGTCGTATCCCACTACCGAGATGTCCGC
ACCAACGCGCAGCCCGGACTCGGTAATGGCGCGCATTGCGCCCAGCGCCATCTGATCGTTGGCAACCAGCATCGCAG
TGGGAACGATGCCCTCATTCAGCATTTGCATGGTTTGTTGAAAACCGGACATGGCACTCCAGTCGCCTTCCCGTTCC
GCTATCGGCTGAATTTGATTGCGAGTGAGATATTTATGCCAGCCAGCCAGACGCAGACGCGCCGAGACAGAACTTAA
TGGGCCCGCTAACAGCGCGATTTGCTGGTGACCCAATGCGACCAGATGCTCCACGCCCAGTCGCGTACCGTCTTCAT
GGGAGAAAATAATACTGTTGATGGGTGTCTGGTCAGAGACATCAAGAAATAACGCCGGAACATTAGTGCAGGCAGCT
TCCACAGCAATGGCATCCTGGTCATCCAGCGGATAGTTAATGATCAGCCCACTGACGCGTTGCGCGAGAAGATTGTG
CACCGCCGCTTTACAGGCTTCGACGCCGCTTCGTTCTACCATCGACACCACCACGCTGGCACCCAGTTGATCGGCGC
GAGATTTAATCGCCGCGACAATTTGCGACGGCGCGTGCAGGGCCAGACTGGAGGTGGCAACGCCAATCAGCAACGAC
TGTTTGCCCGCCAGTTGTTGTGCCACGCGGTTGGGAATGTAATTCAGCTCCGCCATCGCCGCTTCCACTTTTTCCCG
CGTTTTCGCAGAAACGTGGCTGGCCTGGTTCACCACGCGGGAAACGGTCTGATAAGAGACACCGGCATACTCTGCGA
CATCGTATAACGTTACTGGTTTCACATTCACCACCCTGAATTGACTCTCTTCCGGGCGCTATCATGCCATACCGCGA
AAGGTTTTGCGCCATTCGATGGTGTCCGGGATCTCGACGCTCTCCCTTATGCGACTCCTGCATTAGGAAATTAATAC
GACTCACTATAGGGGAATTGTGAGCGGATAACAATTCCCCTGTAGAAATAATTTTGTTTAACTTTAATAAGGAGATA
TACC

b) Insert of BthA coexpressed with BthD, BthS, BthI, BthH, and BthO (see panel a for color coding).

ATGGGCAGCAGCCATCACCATCATCACCACAGCCAGATGGCGACCAAACCAAAAAAGACCAAGGGCGTGAGCGTATC
TATTGAAGGGAAATTACCGAAAATGACGTTAGATATGCCTGTCGATGCAAAAAAAATCAAAGCTATTCAGAAATGTC
TGGAGAACGGTAAACTCACCATTACCATGTCTAAAGTAGATCTGGCAGGCGGTCGTATGGGGAAGGTTATCTGTAC
GATTGAGATTCAAGGAGATATACCATGAAACTGTTGCTGGTTGATAACTTGGTAATGCCGGAGGAGGGCTCGCTGGC
ATATTTGGACGTGCATCCGCATCTGGGACTGCTGGCGCTGGCGGCCGTCGCAGAAGCAGACGGTCACCGCGTGCAAA
TTTACGATCCGAAACGCCTGATTCGTGATGGCACACTGGCTTATGATGCCACCCTTTATGAACGCGCATCTCAAGCG
ATTCTGGCAGCGCGTCCTGATGGCGTCGGTTTCACTGCACTGGGCTGTTCCTTCCTGTTCGCGTTGAATGTGGCGGC
GTTACTGAAACGTCGCGAACCCGATCTGCCGGTGTTGCTGGGCGGTCCGCACGCGACCATGCTGCATCGCCAGATTC
TCGAACGTTTTCCGCAGTTTGATATCATCGTTCGCTATGAAGCCGATGAAATTCTGCCGGCCGTCCTCGATTGCCTG
CCACACCGCACCTTCGACGTGATCCCTGGCCTGAGTTGGCGTGCAACCGGTCGCGGCAGCCCGCTTCGGTTCACGGA
TGGCAAGCCGAAAGTTGAGGATCTGGACCTGCTGCCCATCGCTAGTTACGACCATTATCCGGTTGAAGAACTGGGAC
TGTCGATGCTGCGTATCGAAGCCGGCCGCGGCTGCCCATTTGCGTGCACCTTTTGTTCCACGGCCGGTTTCTTTCAG
CGTTCCTTCCGCCTGAAGTCCGCTGAACGGCTGGTGCGTGAACTTGATATCTTACACCAACGTTACCGTGTCTCCGA
TTTCAAACTGGATCATGATATGTTTACTGTGAATCGTCGTAAAGTCATGGAATTCTGTGAAGCGGTTGCAGGTCGTG
ATTATCGCTGGCGTGCCTCGGCCCGCATCGACTGTGTGGATGAAGCCCTGCTGAAGAAAATGGCGGATGCGGGTTGC
GTTAACCTGTACTTTGGTGTTGAAACAGGTTCAGAACGCATGCAGAAACTGTGTAAAAAGCGTCTGGATCTTCAACG
TGTGGAGCCAATCCTGGCGGCCGCGGACAGCTTCGGCATTGAAACCACCGCCTCGTTCATCACCGGCTACCCGGAAG
AAACGGGTGAAGATCAAGACGATACGCTCGATATGATTGGCCGCTGCGCCCGTCGTGCTAGTTGTCTGACCCAGCTG
CATATGCTGGCGCCGGAACCGGGGACGCCGTTGTTCGACGAACGCGGCGCGGAAATCGCGTATGACGGTTGTGGCGG
TCCCTATAACACGCGTTTATTATCTAGCTCAGATGAACGTGCGGTACTCGGTCACCCTGATATTTTCCAGACTTACT
ACCACTATCCGGCAGCTTTACCGCGTGCACGTTATATCTTTGCTGTTGGGGCTGCCGATCTCCTGCGTCGTGTGGGT
CCAGTAATTTTTGCGTATGCGCTGCGTGGATTCGGCGGCCGTCTGTCCACTCTGATTAGTGCGTGGCGCCAGTTCGC
CGACGAGACCAGTCCGGGAGCCCCGGCGGACGCAGCGGGTCTGGAAGCGTTTATCGCTGCTCGTTTTGGCCGGGGCC
ACCACCTGCATAGTCTGTTCCGTTATGCGTTGCGCCTGTACGCCGCCCGTGCCCCTGCAGATGTGATCGTCGAAGGT
CCATATGAGCCAGATCGTCCATGCATGCTTGCTGAAAACGTCGGGTTGTTGGAAGACATTCACAATTGTGATGCCTT
GGTTGATCGTATTCGCCGTTCAACCGAAGGCGCCCCGATGCTGGGCGATGCCGATGTCGGGGGCTTGGGCACGTATA
TCGTGCAGGGTCACGGCGAAGCCGCCACAGGTTACTGGGTCGAATCCGGTATTGCGACTCTGCTGGATCTGTTTCGT
TCTCCTCGTAGTTGCCGGGCTGTGGCGCAATGGCTGGCAGACGCAACGCGTAACGATGGCATTGATGCCAGCATCTT
TGAACCTCTGCTCCGCCGCGGTATTCTCGTGAGCGCAGGGTAAGATTCAAGGAGATATACCATGCGTCGCACGCTCG
AGCGCTGGTCAGCATTAGACTTGGCTGAGGGTCTGCAATTAGCGCACGCGGTGGCTGCGCTTCAGGCGTTGGGTGTC
CTGGATGCGATGCGTGAGCCAGTTACGGCAGAGACATTGTCTGCCTCTCACGATCTCGACCCGGAATTGCTGCGTGG
AATTCTTGAATACGCCGCATCCCGCACTAATTTGGTGCGTAAGACGGACCGTGGATTCGCGATTTCTGAGCATTATA
CCGCCCAAGCCCGTTTTCTCCTGAATTTATATGCCGGTGCTTACGCAGACAACGCCAGCCGTTTGGCCACATTATTA
CGTCGTCCCGCACTGGCTGGGAGCATGATCGATCTTGTGGCGCACGCTCGTGCATTCGATGCGAGTGAGGCTGGTGG
TGGTGCTCTTGCGTCTATTGTCACCCAATTGCGTCTCAACCATGTGTTGGATCTTGGTTGCGGCGCGGGCGCACTGT
TACGTGAGTTGGCAGCGGATGATCGTGGCTTCGTCGGCTGGGGTCTCGATCGCAATCCTGCGATGTGTAAGGCCGCA
CGTGTCCGTGCACGCCAGGCTGGATTGGCCGCGCGCGTCAAGGTTTTCCACGGAGACGGTTGGAATCCTGGTGCCAG
CGTGCCACCGCGCGTTTTAGCGCGCGTACGTAACGTCGTTGCTAGTCAATTCGTGAACGAAATGTTCCGCGGCGGTA
CGTCCCGCGTTGAGACCTGGTTGCGCCGTATGCGTCGCCTCTTGCCCGGCCGTATGTTGGTTATTAGTGACTATTAC
GGTCGTCTCGGGCACGGCACCCACACCATGCGTCGCGAGACGCTTTTGCATGATTACGTGCAGTTGATCTCAGGTCA
AGGTGTGCCGCCACCTGACTCCGATGCGTGGTTGGCTATGTACCGTGCCGCAGGTTGCCGTCCGCTTCATGTGATCG
AGGACCGCGCGGCGACTACTTGCTTCGTTCATTTAGTTGGTTTATGAGTAGCGAAGCGGTCTAGCATAACCCCTTGG
GGCCTCTAAACGGGTCTTGAGGGGTTTTTTGCCCTTAATACGACTCACTATAGGGGAATTGTGAGCGGATAACAATT
CCAAGGAGATATACAATGAACGCAGGCAAAATTCACGCGGTTATTGCGGCTGGTGCTAAAGACCCACGGTTGATTGA
ACATTGGCGTCGCCGTCCAGATCTGCTGCGGCGCGCGGGTATCGAGCCCGATCATCTGGATCTTCGCGTCCTGGAGA
ATTTTGCGGGGCTGTCCCTTAAAGTTCGCCACAATGGATTGCGCGGTGATTTGCCGTTAAGCTTTCGGTTGCTCTCT
GTGGCGGGATTGGAAATTGAAGTTTTTGCAGCGTACGCATCGGATTGCGCCGAATCAGGGCACGTCTTTGCCGCCAC
ACCGCGTGAACGTGCTCGCGACCTCATGGCCTTCCTTGACCGCTGGCTGGATCCGGCCCAGCGCAACCACGTGCTGT
TATGGGACATCCTGCGCCATGAATACGCTCTCTCGTGCCTGCGTCAGGAATCTGCGGCCCCGCTGCCGGCAGCGCGC
CGTATTATTCGCCACCGTGATCGTGCAACTGGCGGTTGCATTCCAATGCTGAATGGAAGTGTCGCCCAGCACGTTAT
GCATTGTAATCCAGAAGATCTGGCGTCGGCCTTACGCCAAAAAGAACCGCCCCTGGATGCTGTTCCGGCCGCGGACA
CCTACTGTTGCTACTGGCGCAGTGCTGAGACGGGCGAGATCCGTTTTCTCCAGGCAGATACCCTGGGTTATTTTGCA
ATTGAGGCAATCGATGGCCGCCGCTCTGCGGCGGACATCTGTGATCGCCTGCTGGGCGGTCGTCGCCTCCTGCCCCA
GTTTCTGCGCAGCCTGGACCAGCTCGCGGGCATTGGTATTGTACGGTTCGAGCGTCCACTTGGAAGCGCGGCAGCAT

AAGATTCAAGGAGATATACCATGAAAGCTTTTGATCGCGATGCATCAGAACCACGTATCGGCCTGGCCTATGGTCCG
GGCATGGCGGAGTTTGCAGCCCGGAACGCGCATCTCGTGGACTACATTGAAGTTCCGTTTGAACAACTGCGTTTCAG
CCCAGCAGTAGCCGAGCTGCAGCAGACGATTCCGTTCGTGCTGCATTGCGCGAGCTTGAGCGTGGCTGGCTTTGTGC
CGCCGGATGACTCGACTGTAGATGCAATTGAACGCACCGCAGTGCAAACTGGTACCCCGTGGATCGGCGAGCACCTG
GCGTATATCTCGGCGGACCCCGTTGGTGAAGCATTAGGTGGTACCGGGGAGCCGACTTCGCTGTCATACACCCTCTG
TCCGCAGTTATCCGACGAGACCGTCCGTCGCGTGGTGGATAATTTAGCCGCACTGCGTCCTCACTTTCCGGTGCCCT
TGATCGTAGAAAACTCGCCGCAATATTTTCCAATCCCCGGCAGCACTATGGGCATGACCGATTTTATCCGTGCCATT
ACGGATCGCTGTGATGTTGGCTTATTACTTGATTTATCTCACTTTCTGATCACTGCCCACAATACGGGCGCAGAAGT
TCACCGCGAACTGGCGCGCCTGCCACTGGAACGCGTGGTGGAGGTACACTTGTCCGGCATGTCTGTGCAAAGCGGTA
CCGCCTGGGATGATCATTCATTACCGGCCTCACCTATTTTATTTGAACTGTTGGAACGGTTGCTGGACGTGGCTCGC
CCGCGCGCCCTGACATTTGAATACAATTGGAGTCCCTACTTTCCACTGAGCGTGTTGACTACCCACATCGAACGCGC
CCGTCAGCTGATGGGGCTGGCTTAAGATTCAAGGAGATATACCATGAACGGTCTCCCGCCTTCTCTGCCTCGTGTGG
ACTCAACAGAAAGCCTGTTCGCGGAGCCTTTAGCATTCTTAGCGCAAGCCCGTTCTCGTCACGGAGACGTGTTCGTT
ATGCGCGAGCACGGCCCCATCTTTTCCCGCGCATCGGATTGTTCGGGTGTCATTGCTGTCTTTGGAGAGCACCGTTT
ACGTCAAATCCTTACGGACATCGACAACTTCGCGTTACCGATGAGTGCCGCCGCCAAGATGGCATTACCTAAGAACC
TCGTCAATTTAAACCGTGGCCTGCATAGTATGCGCGAGCCGGAGCATGGTCGTCACAAGCGTTTGCTTACCGGTACA
ATTAACCGCGAGCTCTTTGACGCCCACCGTTTCGAAATTCGCGCAGCGCTCAACCGCTTTTGTGAAATGCTCAAGGT
AGATCGCCGTATCAGTGTGGTGAGCCGTATGCGCGAGCTGACGGTCGAGATGGCATCTCATATCTTTCTGGGTGCAC
AATGTCAGGAAGACGATGAGTTGGCGTTTCTTCTTTCAGCCTATTTCACCCTGCGCCGCGAGGCTAGCTCATTAAAC
GCTCGTGACCCGTTGCTTTATCGTGACGAGCTGATCGGTGTGGGACAACAGCTGGACCGCACATTACGCGAACGTAT
CCGCCGCTATCGTAAACGCCCCGTCGACGCCCGCGCCGGTCTGTTACAGCGTCTTGCTACGGCAGGTCCGCCGGGCT
CGCCTGCGTTGTCGGAAGATGAAATTGTGGGTCACGCCAACGTAATGTTCGTGTCTTCCACGGAGCCGGTTGCCATG
TCTCTTACGTGGTTGTTGCTGGTCTTAAGTCAACTGCCTGACTTACGTCGTGCGCTGCGTGCGGAGATCGCGGACCG
TGCGTCTATGCCGGCCAGTACTAATGGCGCGTCGTGGTTAGAGAACGTTGTCAACGAGACGTTGCGCTTATTGACCC
CAAATGCCCTGATGGTCCGCGCCACAACACGCGCAGTGTCACTGCAAGGTGTTGCACTCCCGGCCCGCTGTGAGATT
GTGGTATGTCCATTTCTGGCTCACCGCGAGGCCAAACCATTTCCCGACCCCCACGCATTCTCACCATCGCGCTGGGA
GACCGCGCGTCCCAGTCCTTATGAGTACTTTCCTTTCGGGGCGGGCGGACACTTCTGTGCGGGGCGTAATTTGGCCC
TCTCACTTATTCGCGAAGTGTTGTCGACGCTGTTAAGCCGTTTCGATTTCGTTCTTGACGGTGAGCAAAGCATCGAC
TGGCGCATTCATATTATGCTGATGCCCAAGGGAGACCCGGCTTTGATTGCTCACCCTGTGGATGAACGTGGCGATAC
CCCGAGTCCAAAGTGGCGTGGGCCCATTACCGATTTATTTCACTTCGCCCCTGGGCTTTCATGA

c) Insert of BthA coexpressed with BthD

ATGGGCAGCAGCCATCACCATCATCACCACAGCCAGATGGCGACCAAACCAAAAAAGACCAAGGGCGTGAGCGTATC
TATTGAAGGGAAATTACCGAAAATGACGTTAGATATGCCTGTCGATGCAAAAAAAATCAAAGCTATTCAGAAATGTC
TGGAGAACGGTAAACTCACCATTACCATGTCTAAAGTAGATCTGGCAGGCGGTCGTATGGGGAAGGTTATCTGTAC
GATTGAGATTCAAGGAGATATACCATGAAACTGTTGCTGGTTGATAACTTGGTAATGCCGGAGGAGGGCTCGCTGGC
ATATTTGGACGTGCATCCGCATCTGGGACTGCTGGCGCTGGCGGCCGTCGCAGAAGCAGACGGTCACCGCGTGCAAA
TTTACGATCCGAAACGCCTGATTCGTGATGGCACACTGGCTTATGATGCCACCCTTTATGAACGCGCATCTCAAGCG
ATTCTGGCAGCGCGTCCTGATGGCGTCGGTTTCACTGCACTGGGCTGTTCCTTCCTGTTCGCGTTGAATGTGGCGGC
GTTACTGAAACGTCGCGAACCCGATCTGCCGGTGTTGCTGGGCGGTCCGCACGCGACCATGCTGCATCGCCAGATTC
TCGAACGTTTTCCGCAGTTTGATATCATCGTTCGCTATGAAGCCGATGAAATTCTGCCGGCCGTCCTCGATTGCCTG
CCACACCGCACCTTCGACGTGATCCCTGGCCTGAGTTGGCGTGCAACCGGTCGCGGCAGCCCGCTTCGGTTCACGGA
TGGCAAGCCGAAAGTTGAGGATCTGGACCTGCTGCCCATCGCTAGTTACGACCATTATCCGGTTGAAGAACTGGGAC
TGTCGATGCTGCGTATCGAAGCCGGCCGCGGCTGCCCATTTGCGTGCACCTTTTGTTCCACGGCCGGTTTCTTTCAG
CGTTCCTTCCGCCTGAAGTCCGCTGAACGGCTGGTGCGTGAACTTGATATCTTACACCAACGTTACCGTGTCTCCGA
TTTCAAACTGGATCATGATATGTTTACTGTGAATCGTCGTAAAGTCATGGAATTCTGTGAAGCGGTTGCAGGTCGTG
ATTATCGCTGGCGTGCCTCGGCCCGCATCGACTGTGTGGATGAAGCCCTGCTGAAGAAAATGGCGGATGCGGGTTGC
GTTAACCTGTACTTTGGTGTTGAAACAGGTTCAGAACGCATGCAGAAACTGTGTAAAAAGCGTCTGGATCTTCAACG
TGTGGAGCCAATCCTGGCGGCCGCGGACAGCTTCGGCATTGAAACCACCGCCTCGTTCATCACCGGCTACCCGGAAG
AAACGGGTGAAGATCAAGACGATACGCTCGATATGATTGGCCGCTGCGCCCGTCGTGCTAGTTGTCTGACCCAGCTG
CATATGCTGGCGCCGGAACCGGGGACGCCGTTGTTCGACGAACGCGGCGCGGAAATCGCGTATGACGGTTGTGGCGG
TCCCTATAACACGCGTTTATTATCTAGCTCAGATGAACGTGCGGTACTCGGTCACCCTGATATTTTCCAGACTTACT
ACCACTATCCGGCAGCTTTACCGCGTGCACGTTATATCTTTGCTGTTGGGGCTGCCGATCTCCTGCGTCGTGTGGGT
CCAGTAATTTTTGCGTATGCGCTGCGTGGATTCGGCGGCCGTCTGTCCACTCTGATTAGTGCGTGGCGCCAGTTCGC
CGACGAGACCAGTCCGGGAGCCCCGGCGGACGCAGCGGGTCTGGAAGCGTTTATCGCTGCTCGTTTTGGCCGGGGCC
ACCACCTGCATAGTCTGTTCCGTTATGCGTTGCGCCTGTACGCCGCCCGTGCCCCTGCAGATGTGATCGTCGAAGGT

CCATATGAGCCAGATCGTCCATGCATGCTTGCTGAAAACGTCGGGTTGTTGGAAGACATTCACAATTGTGATGCCTT
GGTTGATCGTATTCGCCGTTCAACCGAAGGCGCCCCGATGCTGGGCGATGCCGATGTCGGGGGCTTGGGCACGTATA
TCGTGCAGGGTCACGGCGAAGCCGCCACAGGTTACTGGGTCGAATCCGGTATTGCGACTCTGCTGGATCTGTTTCGT
TCTCCTCGTAGTTGCCGGGCTGTGGCGCAATGGCTGGCAGACGCAACGCGTAACGATGGCATTGATGCCAGCATCTT
TGAACCTCTGCTCCGCCGCGGTATTCTCGTGAGCGCAGGGTAA

d) BthA coexpressed with BthO
**ATGGGCAGCAGCCATCACCATCATCACCACAGCCAGATGGCGACCAAACCAAAAAAGACCAAGGGCGTGAGCGTATC
TATTGAAGGGAAATTACCGAAAATGACGTTAGATATGCCTGTCGATGCAAAAAAAATCAAAGCTATTCAGAAATGTC
TGGAGAACGGTAAACTCACCATTACCATGTCTAAAGTAGATCTGGCAGGCGGTCGTATGGGGGAAGGTTATCTGTAC
GATTGA**GATTCAAGGAGATATACCATGAACGGTCTCCCGCCTTCTCTGCCTCGTGTGGACTCAACAGAAAGCCTGTT
CGCGGAGCCTTTAGCATTCTTAGCGCAAGCCCGTTCTCGTCACGGAGACGTGTTCGTTATGCGCGAGCACGGCCCCA
TCTTTTCCCGCGCATCGGATTGTTCGGGTGTCATTGCTGTCTTTGGAGAGCACCGTTTACGTCAAATCCTTACGGAC
ATCGACAACTTCGCGTTACCGATGAGTGCCGCCGCCAAGATGGCATTACCTAAGAACCTCGTCAATTTAAACCGTGG
CCTGCATAGTATGCGCGAGCCGGAGCATGGTCGTCACAAGCGTTTGCTTACCGGTACAATTAACCGCGAGCTCTTTG
ACGCCCACCGTTTCGAAATTCGCGCAGCGCTCAACCGCTTTTGTGAAATGCTCAAGGTAGATCGCCGTATCAGTGTG
GTGAGCCGTATGCGCGAGCTGACGGTCGAGATGGCATCTCATATCTTTCTGGGTGCACAATGTCAGGAAGACGATGA
GTTGGCGTTTCTTCTTTCAGCCTATTTCACCCTGCGCCGCGAGGCTAGCTCATTAAACGCTCGTGACCCGTTGCTTT
ATCGTGACGAGCTGATCGGTGTGGGACAACAGCTGGACCGCACATTACGCGAACGTATCCGCCGCTATCGTAAACGC
CCCGTCGACGCCCGCGCCGGTCTGTTACAGCGTCTTGCTACGGCAGGTCCGCCGGGCTCGCCTGCGTTGTCGGAAGA
TGAAATTGTGGGTCACGCCAACGTAATGTTCGTGTCTTCCACGGAGCCGGTTGCCATGTCTCTTACGTGGTTGTTGC
TGGTCTTAAGTCAACTGCCTGACTTACGTCGTGCGCTGCGTGCGGAGATCGCGGACCGTGCGTCTATGCCGGCCAGT
ACTAATGGCGCGTCGTGGTTAGAGAACGTTGTCAACGAGACGTTGCGCTTATTGACCCCAAATGCCCTGATGGTCCG
CGCCACAACACGCGCAGTGTCACTGCAAGGTGTTGCACTCCCGGCCCGCTGTGAGATTGTGGTATGTCCATTTCTGG
CTCACCGCGAGGCCAAACCATTTCCCGACCCCCACGCATTCTCACCATCGCGCTGGGAGACCGCGCGTCCCAGTCCT
TATGAGTACTTTCCTTTCGGGGCGGGCGGACACTTCTGTGCGGGGCGTAATTTGGCCCTCTCACTTATTCGCGAAGT
GTTGTCGACGCTGTTAAGCCGTTTCGATTTCGTTCTTGACGGTGAGCAAAGCATCGACTGGCGCATTCATATTATGC
TGATGCCCAAGGGAGACCCGGCTTTGATTGCTCACCCTGTGGATGAACGTGGCGATACCCCGAGTCCAAAGTGGCGT
GGGCCCATTACCGATTTATTTCACTTCGCCCCTGGGCTTTCATGA

e) BthA coexpressed with BthD and BthO
**ATGGGCAGCAGCCATCACCATCATCACCACAGCCAGATGGCGACCAAACCAAAAAAGACCAAGGGCGTGAGCGTATC
TATTGAAGGGAAATTACCGAAAATGACGTTAGATATGCCTGTCGATGCAAAAAAAATCAAAGCTATTCAGAAATGTC
TGGAGAACGGTAAACTCACCATTACCATGTCTAAAGTAGATCTGGCAGGCGGTCGTATGGGGGAAGGTTATCTGTAC
GATTGA**GATTCAAGGAGATATACCATGAAACTGTTGCTGGTTGATAACTTGGTAATGCCGGAGGAGGGCTCGCTGGC
ATATTTGGACGTGCATCCGCATCTGGGACTGCTGGCGCTGGCGGCCGTCGCAGAAGCAGACGGTCACCGCGTGCAAA
TTTACGATCCGAAACGCCTGATTCGTGATGGCACACTGGCTTATGATGCCACCCTTTATGAACGCGCATCTCAAGCG
ATTCTGGCAGCGCGTCCTGATGGCGTCGGTTTCACTGCACTGGGCTGTTCCTTCCTGTTCGCGTTGAATGTGGCGGC
GTTACTGAAACGTCGCGAACCCGATCTGCCGGTGTTGCTGGGCGGTCCGCACGCGACCATGCTGCATCGCCAGATTC
TCGAACGTTTTCCGCAGTTTGATATCATCGTTCGCTATGAAGCCGATGAAATTCTGCCGGCCGTCCTCGATTGCCTG
CCACACCGCACCTTCGACGTGATCCCTGGCCTGAGTTGGCGTGCAACCGGTCGCGGCAGCCCGCTTCGGTTCACGGA
TGGCAAGCCGAAAGTTGAGGATCTGGACCTGCTGCCCATCGCTAGTTACGACCATTATCCGGTTGAAGAACTGGGAC
TGTCGATGCTGCGTATCGAAGCCGGCCGCGGCTGCCCATTTGCGTGCACCTTTTGTTCCACGGCCGGTTTCTTTCAG
CGTTCCTTCCGCCTGAAGTCCGCTGAACGGCTGGTGCGTGAACTTGATATCTTACACCAACGTTACCGTGTCTCCGA
TTTCAAACTGGATCATGATATGTTTACTGTGAATCGTCGTAAAGTCATGGAATTCTGTGAAGCGGTTGCAGGTCGTG
ATTATCGCTGGCGTGCCTCGGCCCGCATCGACTGTGTGGATGAAGCCCTGCTGAAGAAAATGGCGGATGCGGGTTGC
GTTAACCTGTACTTTGGTGTTGAAACAGGTTCAGAACGCATGCAGAAACTGTGTAAAAAGCGTCTGGATCTTCAACG
TGTGGAGCCAATCCTGGCGGCCGCGGACAGCTTCGGCATTGAAACCACCGCCTCGTTCATCACCGGCTACCCGGAAG
AAACGGGTGAAGATCAAGACGATACGCTCGATATGATTGGCCGCTGCGCCCGTCGTGCTAGTTGTCTGACCCAGCTG
CATATGCTGGCGCCGGAACCGGGGACGCCGTTGTTCGACGAACGCGGCGCGGAAATCGCGTATGACGGTTGTGGCGG
TCCCTATAACACGCGTTTATTATCTAGCTCAGATGAACGTGCGGTACTCGGTCACCCTGATATTTTCCAGACTTACT
ACCACTATCCGGCAGCTTTACCGCGTGCACGTTATATCTTTGCTGTTGGGGCTGCCGATCTCCTGCGTCGTGTGGGT
CCAGTAATTTTTGCGTATGCGCTGCGTGGATTCGGCGGCCGTCTGTCCACTCTGATTAGTGCGTGGCGCCAGTTCGC
CGACGAGACCAGTCCGGGAGCCCCGGCGGACGCAGCGGGTCTGGAAGCGTTTATCGCTGCTCGTTTTGGCCGGGGCC
ACCACCTGCATAGTCTGTTCCGTTATGCGTTGCGCCTGTACGCCGCCCGTGCCCCTGCAGATGTGATCGTCGAAGGT
CCATATGAGCCAGATCGTCCATGCATGCTTGCTGAAAACGTCGGGTTGTTGGAAGACATTCACAATTGTGATGCCTT

GGTTGATCGTATTCGCCGTTCAACCGAAGGCGCCCCGATGCTGGGCGATGCCGATGTCGGGGGCTTGGGCACGTATA
TCGTGCAGGGTCACGGCGAAGCCGCCACAGGTTACTGGGTCGAATCCGGTATTGCGACTCTGCTGGATCTGTTTCGT
TCTCCTCGTAGTTGCCGGGCTGTGGCGCAATGGCTGGCAGACGCAACGCGTAACGATGGCATTGATGCCAGCATCTT
TGAACCTCTGCTCCGCCGCGGTATTCTCGTGAGCGCAGGGTAAGATTCAAGGAGATATACCATGAACGGTCTCCCGC
CTTCTCTGCCTCGTGTGGACTCAACAGAAAGCCTGTTCGCGGAGCCTTTAGCATTCTTAGCGCAAGCCCGTTCTCGT
CACGGAGACGTGTTCGTTATGCGCGAGCACGGCCCCATCTTTTCCCGCGCATCGGATTGTTCGGGTGTCATTGCTGT
CTTTGGAGAGCACCGTTTACGTCAAATCCTTACGGACATCGACAACTTCGCGTTACCGATGAGTGCCGCCGCCAAGA
TGGCATTACCTAAGAACCTCGTCAATTTAAACCGTGGCCTGCATAGTATGCGCGAGCCGGAGCATGGTCGTCACAAG
CGTTTGCTTACCGGTACAATTAACCGCGAGCTCTTTGACGCCCACCGTTTCGAAATTCGCGCAGCGCTCAACCGCTT
TTGTGAAATGCTCAAGGTAGATCGCCGTATCAGTGTGGTGAGCCGTATGCGCGAGCTGACGGTCGAGATGGCATCTC
ATATCTTTCTGGGTGCACAATGTCAGGAAGACGATGAGTTGGCGTTTCTTCTTTCAGCCTATTTCACCCTGCGCCGC
GAGGCTAGCTCATTAAACGCTCGTGACCCGTTGCTTTATCGTGACGAGCTGATCGGTGTGGGACAACAGCTGGACCG
CACATTACGCGAACGTATCCGCCGCTATCGTAAACGCCCCGTCGACGCCCGCGCCGGTCTGTTACAGCGTCTTGCTA
CGGCAGGTCCGCCGGGCTCGCCTGCGTTGTCGGAAGATGAAATTGTGGGTCACGCCAACGTAATGTTCGTGTCTTCC
ACGGAGCCGGTTGCCATGTCTCTTACGTGGTTGTTGCTGGTCTTAAGTCAACTGCCTGACTTACGTCGTGCGCTGCG
TGCGGAGATCGCGGACCGTGCGTCTATGCCGGCCAGTACTAATGGCGCGTCGTGGTTAGAGAACGTTGTCAACGAGA
CGTTGCGCTTATTGACCCCAAATGCCCTGATGGTCCGCGCCACAACACGCGCAGTGTCACTGCAAGGTGTTGCACTC
CCGGCCCGCTGTGAGATTGTGGTATGTCCATTTCTGGCTCACCGCGAGGCCAAACCATTTCCCGACCCCCACGCATT
CTCACCATCGCGCTGGGAGACCGCGCGTCCCAGTCCTTATGAGTACTTTCCTTTCGGGGCGGGCGGACACTTCTGTG
CGGGGCGTAATTTGGCCCTCTCACTTATTCGCGAAGTGTTGTCGACGCTGTTAAGCCGTTTCGATTTCGTTCTTGAC
GGTGAGCAAAGCATCGACTGGCGCATTCATATTATGCTGATGCCCAAGGGAGACCCGGCTTTGATTGCTCACCCTGT
GGATGAACGTGGCGATACCCCGAGTCCAAAGTGGCGTGGGCCCATTACCGATTTATTTCACTTCGCCCCTGGGCTTT
CATGA

f) BthA coexpressed with BthI and BthH

ATGGGCAGCAGCCATCACCATCATCACCACAGCCAGATGGCGACCAAACCAAAAAAGACCAAGGGCGTGAGCGTATC
TATTGAAGGGAAATTACCGAAAATGACGTTAGATATGCCTGTCGATGCAAAAAAAATCAAAGCTATTCAGAAATGTC
TGGAGAACGGTAAACTCACCATTACCATGTCTAAAGTAGATCTGGCAGGCGGTCGTATGGGGGAAGGTTATCTGTAC
GATTGAGTAGCGAAGCGGTCTAGCATAACCCCTTGGGGCCTCTAAACGGGTCTTGAGGGGTTTTTTGCCCTTAATAC
GACTCACTATAGGGGAATTGTGAGCGGATAACAATTCCAAGGAGATATACAATGAACGCAGGCAAAATTCACGCGGT
TATTGCGGCTGGTGCTAAAGACCCACGGTTGATTGAACATTGGCGTCGCCGTCCAGATCTGCTGCGGCGCGCGGGTA
TCGAGCCCGATCATCTGGATCTTCGCGTCCTGGAGAATTTTGCGGGGCTGTCCCTTAAAGTTCGCCACAATGGATTG
CGCGGTGATTTGCCGTTAAGCTTTCGGTTGCTCTCTGTGGCGGGATTGGAAATTGAAGTTTTTGCAGCGTACGCATC
GGATTGCGCCGAATCAGGGCACGTCTTTGCCGCCACACCGCGTGAACGTGCTCGCGACCTCATGGCCTTCCTTGACC
GCTGGCTGGATCCGGCCCAGCGCAACCACGTGCTGTTATGGGACATCCTGCGCCATGAATACGCTCTCTCGTGCCTG
CGTCAGGAATCTGCGGCCCCGCTGCCGGCAGCGCGCCGTATTATTCGCCACCGTGATCGTGCAACTGGCGGTTGCAT
TCCAATGCTGAATGGAAGTGTCGCCCAGCACGTTATGCATTGTAATCCAGAAGATCTGGCGTCGGCCTTACGCCAAA
AAGAACCGCCCCTGGATGCTGTTCCGGCCGCGGACACCTACTGTTGCTACTGGCGCAGTGCTGAGACGGGCGAGATC
CGTTTTCTCCAGGCAGATACCCTGGGTTATTTTGCAATTGAGGCAATCGATGGCCGCCGCTCTGCGGCGGACATCTG
TGATCGCCTGCTGGGCGGTCGTCGCCTCCTGCCCCAGTTTCTGCGCAGCCTGGACCAGCTCGCGGGCATTGGTATTG
TACGGTTCGAGCGTCCACTTGGAAGCGCGGCAGCATAAGATTCAAGGAGATATACCATGAAAGCTTTTGATCGCGAT
GCATCAGAACCACGTATCGGCCTGGCCTATGGTCCGGGCATGGCGGAGTTTGCAGCCCGGAACGCGCATCTCGTGGA
CTACATTGAAGTTCCGTTTGAACAACTGCGTTTCAGCCCAGCAGTAGCCGAGCTGCAGCAGACGATTCCGTTCGTGC
TGCATTGCGCGAGCTTGAGCGTGGCTGGCTTTGTGCCGCCGGATGACTCGACTGTAGATGCAATTGAACGCACCGCA
GTGCAAACTGGTACCCCGTGGATCGGCGAGCACCTGGCGTATATCTCGGCGGACCCCGTTGGTGAAGCATTAGGTGG
TACCGGGGAGCCGACTTCGCTGTCATACACCCTCTGTCCGCAGTTATCCGACGAGACCGTCCGTCGCGTGGTGGATA
ATTTAGCCGCACTGCGTCCTCACTTTCCGGTGCCCTTGATCGTAGAAAACTCGCCGCAATATTTTCCAATCCCCGGC
AGCACTATGGGCATGACCGATTTTATCCGTGCCATTACGGATCGCTGTGATGTTGGCTTATTACTTGATTTATCTCA
CTTTCTGATCACTGCCCACAATACGGGCGCAGAAGTTCACCGCGAACTGGCGCGCCTGCCACTGGAACGCGTGGTGG
AGGTACACTTGTCCGGCATGTCTGTGCAAAGCGGTACCGCCTGGGATGATCATTCATTACCGGCCTCACCTATTTA
TTTGAACTGTTGGAACGGTTGCTGGACGTGGCTCGCCCGCGCGCCCTGACATTTGAATACAATTGGAGTCCCTACTT
TCCACTGAGCGTGTTGACTACCCACATCGAACGCGCCCGTCAGCTGATGGGGCTGGCTTAA

Table S2: Sequence of the initial pSCrhaB2-based vectors used in this study for expression in *Burkholderia*. Each gene is highlighted as follows. *bthA*: bolded black; *bthO*: pink; *bthD*: green; *bthH*: orange; *bthI*: black underlined; *bthS*: blue. The N-terminus of BthA contains a hexa-His tag.

a) Backbone of the pSCrhaB2 vector.

```
TCTAGAGTCGACCTGCAGGCATGCAAGCTTGGCTGTTTTGGCGGATGAGAGAAGATTTTCAGCCTGATACAGATTAA
ATCAGAACGCAGAAGCGGTCTGATAAAACAGAATTTGCCTGGCGGCAGTAGCGCGGTGGTCCCACCTGACCCCATGC
CGAACTCAGAAGTGAAACGCCGTAGCGCCGATGGTAGTGTGGGGTCTCCCCATGCGAGAGTAGGGAACTGCCAGGCA
TCAAATAAAACGAAAGGCTCAGTCGAAAGACTGGGCCTTTCGTTTTATCTGTTGTTTGTCGGTGAACGCTCTCCTGA
GTAGGACAAATCCGCCGGGAGCGGATTTGAACGTTGCGAAGCAACGGCCCGGAGGGTGGCGGGCAGGACGCCCGCCA
TAAACTGCCAGGCATCAAATTAAGCAGAAGGCCATCCTGACGGATGGCCTTTTTGCGTTTCTACAAACTCTTGTTAT
TGGTCGACTTAAACGCCTGGTGCTACGCCTGAATAAGTGATAATAAGCGGATGAATGGCAGAAATTCGAAAGCAAAT
TCGACCCGGTCGTCGGTTCAGGGCAGGGTCGTTAAATAGCCGCTTATGTCTATTGCTGGTTTACCGGTTTATTGACT
ACCGGAAGCAGTGTGACCGTGTGCTTCTCAAATGCCTGAGGCCAGTTTGCTCAGGCTCTCCCCGTGGAGGTAATAAT
TGACGATATGATCATTTATTCTGCCTCCCAGAGCCTGATAAAAACGGTGAATCCGTTAGCGAGGTGCCGCCGGCTTC
CATTCAGGTCGAGGTGGCCCGGCTCCATGCACCGCGACGCAACGCGGGGAGGCAGACAAGGTATAGGGCGGCGAGGC
GGCTACAGCCGATAGTCTGGAACAGCGCACTTACGGGTTGCTGCGCAACCCAAGTGCTACCGGCGCGGCAGCGTGAC
CCGTGTCGGCGGCTCCAACGGCTCGCCATCGTCCAGAAAACACGGCTCATCGGGCATCGGCAGGCGCTGCTGCCCGC
GCCGTTCCCATTCCTCCGTTTCGGTCAAGGCTGGCAGGTCTGGTTCCATGCCCGGAATGCCGGGCTGGCTGGGCGGC
TCCTCGCCGGGGCCGGTCGGTAGTTGCTGCTCGCCCGGATACAGGGTCGGGATGCGGCGCAGGTCGCCATGCCCCAA
CAGCGATTCGTCCTGGTCGTCGTGATCAACCACCACGGCGGCACTGAACACCGACAGGCGCAACTGGTCGCGGGGCT
GGCCCCACGCCACGCGGTCATTGACCACGTAGGCCGACACGGTGCCGGGGCCGTTGAGCTTCACGACGGAGATCCAG
CGCTCGGCCACCAAGTCCTTGACTGCGTATTGGACCGTCCGCAAAGAACGTCCGATGAGCTTGGAAAGTGTCTTCTG
GCTGACCACCACGGCGTTCTGGTGGCCCATCTGCGCCACGAGGTGATGCAGCAGCATTGCCGCCGTGGGTTTCCTCG
CAATAAGCCCGGCCCACGCCTCATGCGCTTTGCGTTCCGTTTGCACCCAGTGACCGGGCTTGTTCTTGGCTTGAATG
CCGATTTCTCTGGACTGCGTGGCCATGCTTATCTCCATGCGGTAGGGGTGCCGCACGGTTGCGGCACCATGCGCAAT
CAGCTGCAACTTTTCGGCAGCGCGACAACAATTATGCGTTGCGTAAAAGTGGCAGTCAATTACAGATTTTCTTTAAC
CTACGCAATGAGCTATTGCGGGGGGTGCCGCAATGAGCTGTTGCGTACCCCCCTTTTTTAAGTTGTTGATTTTTAAG
TCTTTCGCATTTCGCCCTATATCTAGTTCTTTGGTGCCCAAAGAAGGGCACCCCTGCGGGGTTCCCCCACGCCTTCG
GCGCGGCTCCCCCTCCGGCAAAAAGTGGCCCCTCCGGGGCTTGTTGATCGACTGCGCGGCCTTCGGCCTTGCCCAAG
GTGGCGCTGCCCCCTTGGAACCCCCGCACTCGCCGCCGTGAGGCTCGGGGGGCAGGCGGGCGGGCTTCGCCCTTCGA
CTGCCCCCACTCGCATAGGCTTGGGTCGTTCCAGGCGCGTCAAGGCCAAGCCGCTGCGCGGTCGCTGCGCGAGCCTT
GACCCGCCTTCCACTTGGTGTCCAACCGGCAAGCGAAGCGCGCAGGCCGCAGGCCGGAGGCTTTTCCCCAGAGAAAA
TTAAAAAAATTGATGGGGCAAGGCCGCAGGCCGCGCAGTTGGAGCCGGTGGGTATGTGGTCGAAGGCTGGGTAGCCG
GTGGGCAATCCCTGTGGTCAAGCTCGTGGGCAGGCGCAGCCTGTCCATCAGCTTGTCCAGCAGGGTTGTCCACGGGC
CGAGCGAAGCGAGCCAGCCGGTGGCCGCTCGCGGCCATCGTCCACATATCCACGGGCTGGCAAGGGAGCGCAGCGAC
CGCGCAGGGCGAAGCCCGGAGAGCAAGCCCGTAGGGCGCCGCAGCCGCCGTAGGCGGTCACGACTTTGCGAAGCAAA
GTCTAGTGAGTATACTCAAGCATTGAGTGGCCCGCCGGAGGCACCGCCTTGCGCTGCCCCCGTCGAGCCGGTTGGAC
ACCAAAAGGGAGGGGCAGGCATGGCGGCATACGCGATCATGCGATGCAAGAAGCTGGCGAAAATGGGCAACGTGGCG
GCCAGTCTCAAGCACGCCTACCGCGAGCGCGAGACGCCCAACGCTGACGCCAGCAGGACGCCAGAGAACGAGCACTG
GGCGGCCAGCAGCACCGATGAAGCGATGGGCCGACTGCGCGAGTTGCTGCCAGAGAAGCGGCGCAAGGACGCTGTGT
TGGCGGTCGAGTACGTCATGACGGCCAGCCCGGAATGGTGGAAGTCGGCCAGCCAAGAACAGCAGGCGGCGTTCTTC
GAGAAGGCGCACAAGTGGCTGGCGGACAAGTACGGGGCGGATCGCATCGTGACGGCCAGCATCCACCGTGACGAAAC
CAGCCCGCACATGACCGCGTTCGTGGTGCCGCTGACGCAGGACGGCAGGCTGTCGGCCAAGGAGTTCATCGGCAACA
AAGCGCAGATGACCCGCGACCAGACCACGTTTGCGGCCGCTGTGGCCGATCTAGGGCTGCAACGGGGCATCGAGGGC
AGCAAGGCACGTCACACGCGCATTCAGGCGTTCTACGAGGCCCTGGAGCGGCCACCAGTGGGCCACGTCACCATCAG
CCCGCAAGCGGTCGAGCCACGCGCCTATGCACCGCAGGGATTGGCCGAAAAGCTGGGAATCTCAAAGCGCGTTGAGA
CGCCGGAAGCCGTGGCCGACCGGCTGACAAAAGCGGTTCGGCAGGGGTATGAGCCTGCCCTACAGGCCGCCGCAGGA
GCGCGTGAGATGCGCAAGAAGGCCGATCAAGCCCAAGAGACGGCCCGAGACCTTCGGGAGCGCCTGAAGCCCGTTCT
GGACGCCCTGGGGCCGTTGAATCGGGATATGCAGGCCAAGGCCGCCGCGATCATCAAGGCCGTGGGCGAAAAGCTGC
TGACGGAACAGCGGGAAGTCCAGCGCCAGAAACAGGCCCAGCGCCAGCAGGAACGCGGGCGCGCACATTTCCCCGAA
AAGTGCCACCTGACGTCTAAGAAACCATTATTATCATGACATTAACCTATAAAAATAGGCGTATCACGAGGCCCTTT
CGTCTTCGAATAAATACCTGTGACGGAAGATCACTTCGCAGAATAAATAAATCCTGGTGTCCCTGTTGATACCGGGA
AGCCCTGGGCCAACTTTTGGCGAAAATGAGACGTTGATCGGCACGTAAGAGGTTCCAACTTTCACCATAATGAAATA
AGATCACTACCGGGCGTATTTTTTGAGTTATCGAGATTTTCAGGAGCTAAGGAAGCTAAAATGGAGAAAAAAATCAC
```

TGGATATACCACCGTTGATATATCCCAATGGCATCGTAAAGAACATTTTGAGGCATTTCAGTCAGTTGCTCAATGTA
CCTATAACCAGACCGTTCAGCTGGATATTACGGCCTTTTTAGCTTTTGCCATTCTCACCGGATTCAGTCGTCACTCA
TGGTGATTTCTCACTTGATAACCTTATTTTTGACGAGGGGAAATTAATAGGTTGTATTGATGTTGGACGAGTCGGAA
TCGCAGACCGATACCAGGATCTTGCCATCCTATGGAACTGCCTCGGTGAGTTTTCTCCTTCATTACAGAAACGGCTT
TTTCAAAAATATGGTATTGATAATCCTGATATGAATAAATTGCAGTTTCATTTGATGCTCGATGAGTTTTTCTAATC
AGAATTGGTTAATTGGTTGTAACACTGGCAGAGCATTACGCTGACTTGACGGGACGGCGGCTTTGTTGAATAAATCG
AACTTTTGCTGAGTTGAAGGATCAGATCACGCATCTTCCCGACAACGCAGACCGTTCCGTGGCAAAGCAAAAGTTCA
AAATCACCAACTGGTCCACCTACAACAAAGCTCTCATCAACCGTGGCTCCCTCACTTTCTGGCTGGATGATGGGGCG
ATTCAGGCCTGGTATGAGTCAGCAACACCTTCTTCACGAGGCAGACCTCAGCGCCAGAAGGCCGCCAGAGAGGCCGA
GCGCGGCCGTGAGGCTTGGACGCTAGGGCAGGGCATGAAAAAGCCCGTAGCGGGCTGCTACGGGCGTCTGACGCGGT
GGAAAGGGGGAGGGGATGTTGTTTAAATGGCTCTGCTGTAGTGAGTGGTCTACGGGGTCTGACGCTCAGTGGAACGA
AATCGATGATAAGCTGTCAAACATGAGCAGATCCTCTACGCCGGACGCATCGTGGCCGGCATCACCGGCGCCACAGG
TGCGGTTGCTGGCGCCTATATCGCCGACATCACCGATGGGAGATCCTAAGATATCGCTTAGGCCACACGTTCAAGTG
CAGCCACAGGATAAATTTGCACTGAGCCTGGGTGGGATTCGGACTCGACCGCATAGCCTTCAGGAGTGAGTTTTGTG
CAATACCAACCGACGACTTGACCCTGCCAAGCGGCACCAGATTTCTTGCGTACGCGATCCCCTAAGCCAAAGGTGGC
ACTCAGGGGAAGCGCAAACTGCCCTGCAACGGGAGCGTTGGCTTCATCGCTACTTTGACCCATGTCGAATCCTTCTT
GTGAATCTATTATGGCGACAAGCAAATCGAGCTCTGACTGCCTACCCCACAACAACTATCAGAAAGCACCAGCACAA
CGGCTGCCTAACTTTGTTTTAGGGCGACTGCCCTGCTGCGTAACATCGTTGCTGCTCCATAACATCAAACATCGACC
CACGGCGTAACGCGCTTGCTGCTTGGATGCCCGAGGCATAGACTGTACAAAAAAACAGTCATAACAAGCCATGAAAA
CCGCCACTGCGCCGTTACCACCGCTGCGTTCGGTCAAGGTTCTGGACCAGTTGCGTGAGCGCATACGCTACTTGCAT
TACAGTTTACGAACCGAACAGGCTTATGTCAACTGGGTTCGTGCGAGCTCATCGATGCATTTAATCTTTCTGCGAAT
TGAGATGACGCCACTGGCTGGGCGTCATCCCGGTTTCCCGGGTAAACACCACCGAAAAATAGTTACTATCTTCAAAG
CCACATTCGGTCGAAATATCACTGATTAACAGGCGGCTATGCTGGAGAAGATATTGCGCATGACACACTCTGACCTG
TCGCAGATATTGATTGATGGTCATTCCAGTCTGCTGGCGAAATTGCTGACGCAAAACGCGCTCACTGCACGATGCCT
CATCACAAAATTTATCCAGCGCAAAGGACTTTTCAGGCTAGCCGCCAGCCGGGTAATCAGCTTATCCAGCAACGTT
TCGCTGGATGTTGGCGGCAACGAATCACTGGTGTAACGATGGCGATTCAGCAACATCACCAACTGCCCGAACAGCAA
CTCAGCCATTTCGTTAGCAAACGGCACATGCTGACTACTTTCATGCTCAAGCTGACCGATAACCTGCCGCGCCTGCG
CCATCCCCATGCTACCTAAGCGCCAGTGTGGTTGCCCTGCGCTGGCGTTAAATCCCGGAATCGCCCCCTGCCAGTCA
AGATTCAGCTTCAGACGCTCCGGGCAATAAATAATATTCTGCAAAACCAGATCGTTAACGGAAGCGTAGGAGTGTTT
ATCGTCAGCATGAATGTAAAAGAGATCGCCACGGGTAATGCGATAAGGGCGATCGTTGAGTACATGCAGGCCATTAC
CGCGCCAGACAATCACCAGCTCACAAAAATCATGTGTATGTTCAGCAAAGACATCTTGCGGATAACGGTCAGCCACA
GCGACTGCCTGCTGGTCGCTGGCAAAAAAATCATCTTTGAGAAGTTTTAACTGATGCGCCACCGTGGCTACCTCGGC
CAGAGAACGAAGTTGATTATTCGCAATATGGCGTACAAATACGTTGAGAAGATTCGCGTTATTGCAGAAAGCCATCC
CGTCCCTGGCGAATATCACGCGGTGACCAGTTAAACTCTCGGCGAAAAAGCGTCGAAAAGTGGTTACTGTCGCTGAA
TCCACAGCGATAGGCGATGTCAGTAACGCTGGCCTCGCTGTGGCGTAGCAGATGTCGGGCTTTCATCAGTCGCAGGC
GGTTCAGGTATCGCTGAGGCGTCAGTCCCGTTTGCTGCTTAAGCTGCCGATGTAGCGTACGCAGTGAAAGAGAAAAT
TGATCCGCCACGGCATCCCAATTCACCTCATCGGCAAAATGGTCCTCCAGCCAGGCCAGAAGCAAGTTGAGACGTGA
TGCGCTGTTTTCCAGGTTCTCCTGCAAACTGCTTTTACGCAGCAAGAGCAGTAATTGCATAAACAAGATCTCGCGAC
TGGCGGTCGAGGGTAAATCATTTTCCCCTTCCTGCTGTTCCATCTGTGCAACCAGCTGTCGCACCTGCTGCAATACG
CTGTGGTTAACGCGCCAGTGAGACGGATACTGCCCATCCAGCTCTTGTGGCAGCAACTGATTCAGCCCGGCGAGAAA
CTGAAATCGATCCGGCGAGCGATACAGCACATTGGTCAGACACAGATTATCGGTATGTTCATACAGATGCCGATCAT
GATCGCGTACGAAACAGACCGTGCCACCGGTGATGGTATAGGGCTGCCCATTAAACACATGAATACCCGTGCCATGT
TCGACAATCACAATTTCATGAAAATCATGATGATGTTCAGGAAAATCCGCCTGCGGGAGCCGGGGTTCTATCGCCAC
GGACGCGTTACCAGACGGAAAAAAATCCACACTATGTAATACGGTCATACTGGCCTCCTGATGTCGTCAACACGGCG
AAATAGTAATCACGAGGTCAGGTTCTTACCTTAAATTTTCGACGGAAAACCACGTAAAAAACGTCGATTTTTCAAGA
TACAGCGTGAATTTTCAGGAAATGCGGTGAGCATCACATCACCACAATTCAGCAAATTGTGAACATCATCACGTTCA
TCTTTCCCTGGTTGCCAATGGCCCATTTTCCTGTCAGTAACGAGAAGGTCGCGAATTCAGGCGCTTTTTAGACTGGT
CGTAATGAAATTCAGCAGGATCACATA

b) Insert from genomic DNA of *B. thailandensis* E264 encoding all proteins. Removal of the first 833 bp from this insert (black) did not change the mass of BthA after coexpression.

ATGCAAACAAGTGGTTTCCTCGACCTCGGACGGTGGCTGGGTCCACCGGTGGATGGGATTTCTGCCGAATCGAATGT
CGCCGACAGCGACAGGTTTTCGGATGTGGCGGTCTGGCTCGAGTCGTCTGCGAAGACATTTCTTTCCGCCGCAGCGC
GCCGTGCCGTGCGTGTCCGCCACGAGCCAGGGCAATTGCTGGTGCGGCCGACAGGCCGCGCCCGGGCGCGGCGAGGG
TTCGGGAATGCGGCGTGTCCGCTCACGCATGAGATCCGCGCGCATCGGGTATGGCTCGGGCGCTCGCGCCGGGGCCT

TCGTCCTCGACAAGCAGGAGGACGCTCGCGCGCCGGACGTCGCGGAACCCCCGGGGCGGAGCATGAAGGCCTGCTGT
CGTCTCCGATCGAGGGCGCGCTCATGAAGGCCGCACATGCCCGGGCATTTTGCGCGGCGCCGGACGTTTGGATGATT
TCAGGTGCGGCCGCTGATTATCGACTTCGCATTCGTTTGGCGTTTCCATCGGATGCCGACACCTCCGAGCCGGGAAC
GCTGTCGAACGAATTCGACGGGCCGCGCATATTTTCCGCGCCGTCTTGCCGGCGTATTGGCGCGAGCGGCGCTGCGC
GCGAATTTCCGATCGAGAAGCCGTATCGCGATGTCCACCCGGCGCCGATCGAGGACGATGCGACGAAGCTCGAACGT
CATGCGCGAGCGCGCGGGCGGCGTGGCGCGCGCGGTAACGTAGGCGCCGTCAGACGTGATACTGTGACGACGCCATG
CGCCATGGCGATCAGCCGGCGTAGCCAGGATCGGCACGAGTCGTTCACGGATACCGCTTGCAAATGAACGGTCTACC
TCCGTCGCTTCCGCGGGTCGACAGCACCGAGTCGCTTTTTGCGGAGCCGCTCGCGTTTCTCGCACAAGCGCGCTCGC
GGCATGGTGATGTCTTCGTCATGCGGGAGCATGGTCCGATTTTTTCCCGCGCGAGCGATTGCAGCGGCGTGATAGCG
GTATTCGGCGAGCATCGGCTTCGACAGATATTGACCGATATAGACAATTTCGCGTTGCCGATGTCCGCGGCGGCGAA
AATGGCATTGCCGAAAAACCTCGTCAACCTCAATCGCGGCTTGCACAGCATGCGCGAGCCGGAGCATGGCCGGCACA
AGCGCCTCTTGACGGGAACGATCAATCGCGAGTTGTTCGACGCGCACCGCTTCGAAATACGAGCGGCTTTGAACCGC
TTTTGCGAAATGCTGAAAGTGGACCGCCGGATTTCAGTGGTCAGCCGGATGCGCGAGCTGACCGTGGAGATGGCGTC
TCATATCTTTCTTGGGGCGCAGTGCCAAGAGGATGACGAACTGGCGTTTCTTTTAAGCGCCTATTTTACTTTGCGAC
GCGAAGCGTCTTCGCTGAACGCGCGCGATCCCCTGCTGTACCGGGACGAACTGATCGGCGTCGGGCAGCAACTCGAC
CGCACATTGCGGGAGCGGATCAGGCGGTATCGAAAGCGTCCCGTCGACGCTCGTGCGGGGCTGCTGCAACGTCTGGC
AACGGCCGGACCGCCGGGCTCGCCCGCGCTTTCCGAGGACGAGATAGTCGGCCATGCCAACGTGATGTTCGTGTCGA
GCACCGAGCCCGTCGCGATGTCGCTGACGTGGCTGCTGCTCGTTCTGTCGCAGCTGCCCGATCTGAGGCGCGCGCTT
CGCGCGGAAATTGCCGATCGCGCGTCGATGCCGGCATCGACGAACGGCGCGTCGTGGCTCGAGAACGTCGTCAACGA
GACGCTCCGGCTCCTGACGCCCAACGCGCTGATGGTTCGCGCGACGACGCGCGCGGTCTCGCTGCAAGGCGTCGCGC
TTCCGGCGCGTTGCGAGATCGTCGTGTGTCCGTTCCTCGCGCATCGCGAAGCCAAGCCGTTTCCGGACCCGCATGCA
TTCTCGCCGTCCCGGTGGGAGACGGCCAGGCCGTCTCCGTACGAGTATTTTCCGTTCGGCGCCGGCGGTCATTTTTG
CGCGGGGCGAAATCTCGCGCTCTCGCTGATTCGCGAAGTACTGTCAACGCTGCTATCGCGCTTCGATTTCGTTTTGG
ATGGCGAACAGTCTATCGACTGGCGCATTCATATCATGTTGATGCCGAAAGGCGACCCGGCACTCATCGCGCATCCC
GTCGATGAGCGCGGTGACACGCCGTCGCCGAAATGGCGCGGGCCGATAACCGATCTATTCCATTTCGCGCCGGGGCT
TTCTTGACATTTACCAGGGCGCATCCTTTAATCAGAAGCGAATCGGCATGCATGCCCATCGGGATTTTTTCCAGTCA
TTGATCCGCATGGTTCTTTTCACGGGTTATTCCGCGCTCACACGCTAAGCGGCGCGTCCTGCCTACAACAAAGGAAC
AATC**ATGCATCACCATCACCATCACATGGCTACGAAACCCAAAAAAACAAAGGGCGTTTCTGTTTCCATCGAAGGGA**
AGCTGCCCAAGATGACCCTGGATATGCCGGTGGACGCGAAAAAGATCAAGGCCATCCAGAAATGCCTGGAAAACGGC
AAGCTGACCATCACGATGAGCAAGGTGGATCTTGCCGGCGGCCGCATGGGCGAGGGCTATCTCTACGACTGATGTCC
GGCGGCGCTCTGGTTTTCGCCGGCGGCGTCGCGATCTCGCCGCCTCGATGCTCGGGCCTCGGAGGGAACGTGCCGCC
GAACGCGTGCGTTTGGCGCGCGGTCGCGCGATAACGGGCGATGAATTCCGGGCTTCGGCAGTTCGACCTTAAGGGCG
CGGGAAGCCTATGAAAGCCTTCGATCGCGATGCATCGGAGCCTCGGATCGGTCTCGCGTATGGGCCCGGGATGGCGG
AATTCGCCGCACGGAACGCGCACCTCGTCGATTACATCGAAGTGCCGTTCGAGCAATTGCGATTCTCGCCGGCGGTC
GCCGAGCTTCAGCAAACCATTCCGTTCGTTCTGCACTGCGCCAGCTTGTCCGTCGCGGGATTCGTGCCGCCCGACGA
TTCGACGGTCGACGCAATCGAGCGCACGGCCGTGCAGACCGGCACGCCATGGATAGGCGAACATCTCGCGTACATTT
CGGCCGATCCCGTTGGCGAAGCGCTGGGCGGGACCGGTGAGCCCACCTCGTTGTCGTACACGTTGTGCCCTCAGTTG
AGCGACGAGACGGTACGCCGCGTCGTCGACAATCTCGCCGCGCTTCGTCCGCATTTTCCGGTTCCACTGATCGTCGA
AAACTCCCCGCAGTACTTTCCGATACCGGGCAGCACGATGGGAATGACGGACTTCATTCGCGCGATCACGGACCGAT
GCGACGTCGGTTTGCTCCTGGACCTGAGTCACTTCCTGATCACGGCGCACAACACGGGCGCAGAGGTGCATCGGGAA
CTCGCCCGGCTTCCGCTGGAGCGCGTGGTCGAAGTTCATCTGTCCGGAATGAGCGTGCAGTCCGGCACGGCCTGGGA
CGACCATTCGCTGCCGGCATCGCCGATCCTGTTCGAACTGCTCGAGCGCCTGCTCGACGTGGCGCGGCCTCGCGCGC
TGACCTTCGAATACAACTGGTCGCCATACTTCCCGCTGTCGGTCCTGACGACGCATATCGAGCGCGCACGCCAGTTG
ATGGGGCTCGCATGAACGCAGGGAAAATACACGCCGTGATCGCCGCCGGCGCCAAGGACCCGCGGCTCATCGAGCAT
TGGCGGCGGCGTCCGGACCTGCTGCGCCGCGCAGGAATCGAGCCGGATCATCTCGATTTGCGCGTATTGGAGAATTT
CGCCGGGTTGAGCCTGAAGGTGCGTCACAACGGCCTGCGCGGGGATCTGCCGCTGAGTTTCAGGTTGTTGAGCGTGG
CCGGCCTCGAGATCGAGGTCTTCGCCGCGTATGCGTCGGACTGCGCGGAAAGCGGCCATGTATTCGCCGCCACGCCG
CGGGAACGCGCGCGCGATCTGATGGCGTTCCTCGACAGATGGCTGGACCCCGCGCAACGAAATCACGTTCTGCTGTG
GGACATCCTTCGTCATGAATATGCGCTTTCGTGCCTGCGGCAAGAAAGCGCCGCGCCGCTACCCGCCGCTCGCCGCA
TCATCCGCCATCGGGATCGCGCGACAGGCGGCTGCATTCCGATGCTCAACGGCTCCGTCGCTCAGCACGTGATGCAT
TGCAATCCGGAAGACCTGGCGTCGGCGCTGCGTCAGAAGGAGCCGCCGCTCGATGCGGTTCCCGCCGCCGACACTTA
CTGCTGCTATTGGCGCTCCGCCGAAACGGGCGAAATCCGGTTTCTGCAGGCCGATACGCTCGGCTATTTCGCGATCG
AAGCGATCGACGGCCGGCGATCCGCCGCCGACATCTGCGATCGGCTGCTCGGCGGCCGCCGTCTGCTGCCGCAGTTC
CTCCGGTCGCTCGACCAACTGGCCGGAATCGGGATCGTGCGGTTCGAGAGGCCGCTCGGAAGCGCTGCGGCATGAAA
CTGCTGCTCGTCGACAACCTCGTCATGCCGGAAGAGGGCTCGCTCGCGTACCTCGACGTGCATCCGCATCTCGGGCT
GCTCGCGCTGGCCGCGGTCGCCGAAGCCGACGGCCATCGCGTGCAGATCTACGATCCGAAACGACTGATCAGGGACG
GCACGCTGGCTTACGATGCGACGCTTTACGAGCGGGCGAGCCAAGCGATTCTCGCGGCGCGTCCGGACGGGGTCGGC

TTCACGGCGCTCGGCTGCAGCTTTCTGTTTGCGCTCAATGTCGCGGCGCTGTTGAAGCGGCGCGAGCCCGACCTGCC
CGTCTTGCTCGGCGGGCCGCACGCGACGATGCTGCACCGGCAGATCCTCGAGCGGTTCCCGCAGTTCGACATCATCG
TCCGGTACGAGGCGGATGAAATCCTGCCCGCCGTGCTCGACTGCCTCCCGCACCGGACATTCGACGTGATTCCCGGC
TTGAGCTGGCGCGCGACGGGCCGCGGCTCGCCGTTGCGCTTCACCGACGGCAAGCCCAAGGTCGAGGATCTGGACCT
GCTGCCGATCGCCTCGTACGACCATTATCCGGTTGAGGAACTGGGGCTCTCGATGCTGCGCATCGAGGCCGGCCGAG
GGTGTCCGTTCGCATGCACATTCTGCTCGACGGCCGGTTTCTTTCAACGCAGCTTCCGCCTCAAATCGGCCGAGCGC
CTGGTTCGCGAACTGGATATCCTTCACCAGCGTTATCGCGTGTCGGACTTCAAGCTCGACCACGACATGTTCACCGT
GAATCGCCGCAAGGTAATGGAGTTTTGCGAGGCGGTCGCCGGGCGCGACTATCGGTGGCGCGCGTCGGCGCGGATCG
ACTGCGTCGACGAAGCATTGTTGAAGAAGATGGCCGACGCGGGCTGCGTGAATCTTTATTTCGGCGTGGAGACGGGC
TCCGAGCGCATGCAAAAGCTCTGCAAGAAGCGGCTGGATCTGCAACGGGTCGAGCCCATTCTGGCGGCGGCGGATTC
ATTCGGAATCGAGACGACCGCGTCGTTCATCACCGGCTATCCCGAGGAAACCGGGGAGGATCAGGACGACACGCTGG
ACATGATCGGGCGCTGCGCTCGGCGAGCGTCTTGCCTCACGCAACTGCACATGCTCGCGCCGGAGCCCGGCACACCG
CTGTTCGACGAACGCGGCGCGGAAATCGCCTACGACGGCTGTGGCGGGCCTTACAACACGCGCCTGCTGAGTTCGTC
GGACGAGCGTGCGGTGCTCGGCCATCCCGACATATTCCAAACCTACTATCACTACCCCGCCGCGCTGCCGCGTGCGC
GATACATCTTTGCCGTCGGGGCGGCGGATCTGCTGCGCCGGGTCGGGCCGGTCATTTTCGCGTATGCGTTGCGGGGG
TTCGGAGGGCGGCTGAGCACGCTGATATCGGCGTGGCGACAATTCGCCGACGAAACGTCGCCCGGCGCGCCCGCGGA
CGCGGCGGGGCTCGAGGCCTTCATTGCCGCGCGGTTCGGCCGCGGCCATCACCTGCATTCTCTTTTTCGGTACGCGT
TGCGGCTCTATGCGGCCCGCGCTCCGGCGGACGTAATCGTCGAGGGGCCCTATGAGCCGGACCGGCCTTGCATGCTG
GCCGAAAACGTCGGTCTGCTGGAGGACATTCACAATTGCGACGCATTGGTCGACCGGATCAGGCGATCGACCGAAGG
CGCGCCGATGCTCGGCGACGCGGATGTCGGCGGGCTCGGAACATATATCGTTCAGGGGCACGGCGAGGCCGCCACGG
GCTATTGGGTGGAGTCCGGCATCGCTACGCTTCTCGATCTGTTCAGGTCGCCCCGTTCGTGCCGCGCCGTTGCGCAA
TGGCTGGCGGACGCCACCCGCAATGACGGCATAGACGCTTCGATCTTCGAGCCGCTGCTGCGCAGAGGCATCCTGGT
GTCGGCCGGTTAAATCCATTTCGTCCATGGTGCGTCGCACACTCGAACGTTGGTCCGCGCTGGATCTCGCGGAAGGG
CTTCAACTGGCGCATGCCGTCGCCGCGCTGCAGGCGCTCGGCGTTCTGGACGCGATGAGGGAGCCGGTCACGGCGGA
AACGTTGAGCGCCAGCCATGATCTCGATCCCGAGTTGCTGCGCGGCATCCTCGAATATGCGGCGTCGAGAACGAATC
TGGTTCGCAAGACGGACCGCGGATTCGCGATTTCCGAGCATTACACCGCGCAAGCGCGCTTTCTGTTGAACCTGTAC
GCCGGCGCTTATGCCGATAACGCTTCACGGCTGGCGACGCTGCTCCGGCGTCCGGCATTGGCGGGCTCCATGATCGA
CCTCGTCGCGCACGCGCGCGCCTTCGACGCGAGCGAGGCCGGCGGTGGAGCGCTGGCGTCGATCGTCACGCAATTGC
GTCTCAATCATGTGCTGGATCTGGGATGCGGCGCCGGCGCGCTGCTGCGCGAGTTGGCGGCGGACGATCGCGGATTC
GTCGGCTGGGGACTGGATCGCAATCCCGCGATGTGCAAGGCGGCGCGGGTTCGAGCGCGGCAAGCGGGGCTCGCCGC
GCGCGTGAAGGTGTTCCACGGCGACGGCTGGAATCCCGGGGCGTCGGTCCCGCCGCGCGTGCTGGCGCGCGTGCGCA
ACGTCGTCGCGAGCCAGTTCGTCAACGAGATGTTTCGCGGCGGCACATCGCGAGTGGAAACGTGGCTTCGGCGCATG
CGGCGCTTGCTGCCCGGCCGCATGCTCGTGATATCGGACTATTACGGCCGCCTCGGCCACGGCACTCACACGATGCG
CCGCGAGACGCTGCTGCATGACTATGTGCAGCTCATATCGGGGCAGGGCGTGCCGCCGCCCGATTCCGACGCGTGGT
TGGCCATGTATCGAGCAGCCGGTTGCCGGCCTCTGCACGTGATCGAAGACCGGGCGGCCACGACATGCTTCGTCCAT
CTGGTGGGGCTATAG

c) Insert from genomic DNA of *B. thailandensis* E264 encoding all proteins, with the gene encoding BthO removed.

ATGAACGGTCTACCTCCGTCGCTTCCGCGGGTCGACAGCACCGAGTCGCTTTTTGCGGAGCCGCTCGCGTTTCTCGC
ACAAGCGCGCTCGCGGCATGGTGATCCGGCACTCATCGCGCATCCCGTCGATGAGCGCGGTGACACGCCGTCGCCGA
AATGGCGCGGGCCGATAACCGATCTATTCCATTTCGCGCCGGGGCTTTCTTGACATTTACCAGGGCGCATCCTTTAA
TCAGAAGCGAATCGGCATGCATGCCCATCGGGATTTTTTCCAGTCATTGATCCGCATGGTTCTTTTCACGGGTTATT
CCGCGCTCACACGCTAAGCGGCGCGTCCTGCCTACAACAAAGGAACAATC**ATGCATCACCATCACCATCACATGGCT**
ACGAAACCCAAAAAAACAAAGGGCGTTTCTGTTTCCATCGAAGGGAAGCTGCCCAAGATGACCCTGGATATGCCGGT
GGACGCGAAAAAGATCAAGGCCATCCAGAAATGCCTGGAAAACGGCAAGCTGACCATCACGATGAGCAAGGTGGATC
TTGCCGGCGGCCGCATGGGCGAGGGCTATCTCTACGACTGATGTCCGGCGGCGCTCTGGTTTTCGCCGGCGGCGTCG
CGATCTCGCCGCCTCGATGCTCGGGCCTCGGAGGGAACGTGCCGCCGAACGCGTGCGTTTGGCGCGCGGTCGCGCGA
TAACGGGCGATGAATTCCGGGCTTCGGCAGTTCGACCTTAAGGGCGCGGGAAGCCTATGAAAGCCTTCGATCGCGAT
GCATCGGAGCCTCGGATCGGTCTCGCGTATGGGCCCGGGATGGCGGAATTCGCCGCACGGAACGCGCACCTCGTCGA
TTACATCGAAGTGCCGTTCGAGCAATTGCGATTCTCGCCGGCGGTCGCCGAGCTTCAGCAAACCATTCCGTTCGTTC
TGCACTGCGCCAGCTTGTCCGTCGCGGGATTCGTGCCGCCCGACGATTCGACGGTCGACGCAATCGAGCGCACGGCC
GTGCAGACCGGCACGCCATGGATAGGCGAACATCTCGCGTACATTTCGGCCGATCCCGTTGGCGAAGCGCTGGGCGG
GACCGGTGAGCCCACCTCGTTGTCGTACACGTTGTGCCCTCAGTTGAGCGACGAGACGGTACGCCGCGTCGTCGACA
ATCTCGCCGCGCTTCGTCCGCATTTTCCGGTTCCACTGATCGTCGAAAACTCCCCGCAGTACTTTCCGATACCGGGC

AGCACGATGGGAATGACGGACTTCATTCGCGCGATCACGGACCGATGCGACGTCGGTTTGCTCCTGGACCTGAGTCA
CTTCCTGATCACGGCGCACAACACGGGCGCAGAGGTGCATCGGGAACTCGCCCGGCTTCCGCTGGAGCGCGTGGTCG
AAGTTCATCTGTCCGGAATGAGCGTGCAGTCCGGCACGGCCTGGGACGACCATTCGCTGCCGGCATCGCCGATCCTG
TTCGAACTGCTCGAGCGCCTGCTCGACGTGGCGCGGCCTCGCGCGCTGACCTTCGAATACAACTGGTCGCCATACTT
CCCGCTGTCGGTCCTGACGACGCATATCGAGCGCGCACGCCAGTTGATGGGGCTCGCATGAACGCAGGGAAAATACA
CGCCGTGATCGCCGCCGGCGCCAAGGACCCGCGGCTCATCGAGCATTGGCGGCGGCGTCCGGACCTGCTGCGCCGCG
CAGGAATCGAGCCGGATCATCTCGATTTGCGCGTATTGGAGAATTTCGCCGGGTTGAGCCTGAAGGTGCGTCACAAC
GGCCTGCGCGGGGATCTGCCGCTGAGTTTCAGGTTGTTGAGCGTGGCCGGCCTCGAGATCGAGGTCTTCGCCGCGTA
TGCGTCGGACTGCGCGGAAAGCGGCCATGTATTCGCCGCCACGCCGCGGGAACGCGCGCGCGATCTGATGGCGTTCC
TCGACAGATGGCTGGACCCCGCGCAACGAAATCACGTTCTGCTGTGGGACATCCTTCGTCATGAATATGCGCTTTCG
TGCCTGCGGCAAGAAAGCGCCGCGCCGCTACCCGCCGCTCGCCGCATCATCCGCCATCGGGATCGCGCGACAGGCGG
CTGCATTCCGATGCTCAACGGCTCCGTCGCTCAGCACGTGATGCATTGCAATCCGGAAGACCTGGCGTCGGCGCTGC
GTCAGAAGGAGCCGCCGCTCGATGCGGTTCCCGCCGCCGACACTTACTGCTGCTATTGGCGCTCCGCCGAAACGGGC
GAAATCCGGTTTCTGCAGGCCGATACGCTCGGCTATTTCGCGATCGAAGCGATCGACGGCCGGCGATCCGCCGCCGA
CATCTGCGATCGGCTGCTCGGCGGCCGCCGTCTGCTGCCGCAGTTCCTCCGGTCGCTCGACCAACTGGCCGGAATCG
GGATCGTGCGGTTCGAGAGGCCGCTCGGAAGCGCTGCGGCATGAAACTGCTGCTCGTCGACAACCTCGTCATGCCGG
AAGAGGGCTCGCTCGCGTACCTCGACGTGCATCCGCATCTCGGGCTGCTCGCGCTGGCCGCGGTCGCCGAAGCCGAC
GGCCATCGCGTGCAGATCTACGATCCGAAACGACTGATCAGGGACGGCACGCTGGCTTACGATGCGACGCTTTACGA
GCGGGCGAGCCAAGCGATTCTCGCGGCGCGTCCGGACGGGGTCGGCTTCACGGCGCTCGGCTGCAGCTTTCTGTTTG
CGCTCAATGTCGCGGCGCTGTTGAAGCGGCGCGAGCCCGACCTGCCCGTCTTGCTCGGCGGGCCGCACGCGACGATG
CTGCACCGGCAGATCCTCGAGCGGTTCCCGCAGTTCGACATCATCGTCCGGTACGAGGCGGATGAAATCCTGCCCGC
CGTGCTCGACTGCCTCCCGCACCGGACATTCGACGTGATTCCCGGCTTGAGCTGGCGCGCGACGGGCCGCGGCTCGC
CGTTGCGCTTCACCGACGGCAAGCCCAAGGTCGAGGATCTGGACCTGCTGCCGATCGCCTCGTACGACCATTATCCG
GTTGAGGAACTGGGGCTCTCGATGCTGCGCATCGAGGCCGGCCGAGGGTGTCCGTTCGCATGCACATTCTGCTCGAC
GGCCGGTTTCTTTCAACGCAGCTTCCGCCTCAAATCGGCCGAGCGCCTGGTTCGCGAACTGGATATCCTTCACCAGC
GTTATCGCGTGTCGGACTTCAAGCTCGACCACGACATGTTCACCGTGAATCGCCGCAAGGTAATGGAGTTTTGCGAG
GCGGTCGCCGGGCGCGACTATCGGTGGCGCGCGTCGGCGCGGATCGACTGCGTCGACGAAGCATTGTTGAAGAAGAT
GGCCGACGCGGGCTGCGTGAATCTTTATTTCGGCGTGGAGACGGGCTCCGAGCGCATGCAAAAGCTCTGCAAGAAGC
GGCTGGATCTGCAACGGGTCGAGCCCATTCTGGCGGCGGCGGATTCATTCGGAATCGAGACGACCGCGTCGTTCATC
ACCGGCTATCCCGAGGAAACCGGGGAGGATCAGGACGACACGCTGGACATGATCGGGCGCTGCGCTCGGCGAGCGTC
TTGCCTCACGCAACTGCACATGCTCGCGCCGGAGCCCGGCACACCGCTGTTCGACGAACGCGGCGCGGAAATCGCCT
ACGACGGCTGTGGCGGGCCTTACAACACGCGCCTGCTGAGTTCGTCGGACGAGCGTGCGGTGCTCGGCCATCCCGAC
ATATTCCAAACCTACTATCACTACCCCGCCGCGCTGCCGCGTGCGCGATACATCTTTGCCGTCGGGGCGGCGGATCT
GCTGCGCCGGGTCGGCCGGTCATTTTCGCGTATGCGTTGCGGGGGTTCGGAGGGCGGCTGAGCACGCTGATATCGG
CGTGGCGACAATTCGCCGACGAAACGTCGCCCGGCGCGCCCGCGGACGCGGCGGGGCTCGAGGCCTTCATTGCCGCG
CGGTTCGGCCGCGGCCATCACCTGCATTCTCTTTTTCGGTACGCGTTGCGGCTCTATGCGGCCCGCGCTCCGGCGGA
CGTAATCGTCGAGGGGCCCTATGAGCCGGACCGGCCTTGCATGCTGGCCGAAAACGTCGGTCTGCTGGAGGACATTC
ACAATTGCGACGCATTGGTCGACCGGATCAGGCGATCGACCGAAGGCGCGCCGATGCTCGGCGACGCGGATGTCGGC
GGGCTCGGAACATATATCGTTCAGGGGCACGGCGAGGCCGCCACGGGCTATTGGGTGGAGTCCGGCATCGCTACGCT
TCTCGATCTGTTCAGGTCGCCCCGTTCGTGCCGCGCCGTTGCGCAATGGCTGGCGGACGCCACCCGCAATGACGGCA
TAGACGCTTCGATCTTCGAGCCGCTGCTGCGCAGAGGCATCCTGGTGTCGGCCGGTTAAATCCATTTCGTCCATGGT
GCGTCGCACACTCGAACGTTGGTCCGCGCTGGATCTCGCGGAAGGGCTTCAACTGGCGCATGCCGTCGCCGCGCTGC
AGGCGCTCGGCGTTCTGGACGCGATGAGGGAGCCGGTCACGGCGGAAACGTTGAGCGCCAGCCATGATCTCGATCCC
GAGTTGCTGCGCGGCATCCTCGAATATGCGGCGTCGAGAACGAATCTGGTTCGCAAGACGGACCGCGGATTCGCGAT
TTCCGAGCATTACACCGCGCAAGCGCGCTTTCTGTTGAACCTGTACGCCGGCGCTTATGCCGATAACGCTTCACGGC
TGGCGACGCTGCTCCGGCGTCCGGCATTGGCGGGCTCCATGATCGACCTCGTCGCGCACGCGCGCGCCTTCGACGCG
AGCGAGGCCGGCGGTGGAGCGCTGGCGTCGATCGTCACGCAATTGCGTCTCAATCATGTGCTGGATCTGGGATGCGG
CGCCGGCGCGCTGCTGCGCGAGTTGGCGGCGGACGATCGCGGATTCGTCGGCTGGGGACTGGATCGCAATCCCGCGA
TGTGCAAGGCGGCGCGGGTTCGAGCGCGGCAAGCGGGGCTCGCCGCGCGCGTGAAGGTGTTCCACGGCGACGGCTGG
AATCCCGGGGCGTCGGTCCCGCCGCGCGTGCTGGCGCGCGTGCGCAACGTCGTCGCGAGCCAGTTCGTCAACGAGAT
GTTTCGCGGCGGCACATCGCGAGTGGAAACGTGGCTTCGGCGCATGCGGCGCTTGCTGCCCGGCCGCATGCTCGTGA
TATCGGACTATTACGGCCGCCTCGGCCACGGCACTCACACGATGCGCCGCGAGACGCTGCTGCATGACTATGTGCAG
CTCATATCGGGGCAGGGCGTGCCGCCGCCCGATTCCGACGCGTGGTTGGCCATGTATCGAGCAGCCGGTTGCCGGCC
TCTGCACGTGATCGAAGACCGGGCGGCCACGACATGCTTCGTCCATCTGGTGGGGCTATAG

d) Insert from genomic DNA of *B. thailandensis* E264 encoding all proteins, with the gene encoding BthH removed.

ATGAACGGTCTACCTCCGTCGCTTCCGCGGGTCGACAGCACCGAGTCGCTTTTTGCGGAGCCGCTCGCGTTTCTCGC
ACAAGCGCGCTCGCGGCATGGTGATGTCTTCGTCATGCGGGAGCATGGTCCGATTTTTTCCCGCGCGAGCGATTGCA
GCGGCGTGATAGCGGTATTCGGCGAGCATCGGCTTCGACAGATATTGACCGATATAGACAATTTCGCGTTGCCGATG
TCCGCGGCGGCGAAAATGGCATTGCCGAAAAACCTCGTCAACCTCAATCGCGGCTTGCACAGCATGCGCGAGCCGGA
GCATGGCCGGCACAAGCGCCTCTTGACGGGAACGATCAATCGCGAGTTGTTCGACGCGCACCGCTTCGAAATACGAG
CGGCTTTGAACCGCTTTTGCGAAATGCTGAAAGTGGACCGCCGGATTTCAGTGGTCAGCCGGATGCGCGAGCTGACC
GTGGAGATGGCGTCTCATATCTTTCTTGGGGCGCAGTGCCAAGAGGATGACGAACTGGCGTTTCTTTTAAGCGCCTA
TTTTACTTTGCGACGCGAAGCGTCTTCGCTGAACGCGCGCGATCCCCTGCTGTACCGGGACGAACTGATCGGCGTCG
GGCAGCAACTCGACCGCACATTGCGGGAGCGGATCAGGCGGTATCGAAAGCGTCCCGTCGACGCTCGTGCGGGGCTG
CTGCAACGTCTGGCAACGGCCGGACCGCCGGGCTCGCCCGCGCTTTCCGAGGACGAGATAGTCGGCCATGCCAACGT
GATGTTCGTGTCGAGCACCGAGCCCGTCGCGATGTCGCTGACGTGGCTGCTGCTCGTTCTGTCGCAGCTGCCCGATC
TGAGGCGCGCGCTTCGCGCGGAAATTGCCGATCGCGCGTCGATGCCGGCATCGACGAACGGCGCGTCGTGGCTCGAG
AACGTCGTCAACGAGACGCTCCGGCTCCTGACGCCCAACGCGCTGATGGTTCGCGCGACGACGCGCGCGGTCTCGCT
GCAAGGCGTCGCGCTTCCGGCGCGTTGCGAGATCGTCGTGTGTCCGTTCCTCGCGCATCGCGAAGCCAAGCCGTTTC
CGGACCCGCATGCATTCTCGCCGTCCCGGTGGGAGACGGCCAGGCCGTCTCCGTACGAGTATTTTCCGTTCGGCGCC
GGCGGTCATTTTTGCGCGGGGCGAAATCTCGCGCTCTCGCTGATTCGCGAAGTACTGTCAACGCTGCTATCGCGCTT
CGATTTCGTTTTGGATGGCGAACAGTCTATCGACTGGCGCATTCATATCATGTTGATGCCGAAAGGCGACCCGGCAC
TCATCGCGCATCCCGTCGATGAGCGCGGTGACACGCCGTCGCCGAAATGGCGCGGGCCGATAACCGATCTATTCCAT
TTCGCGCCGGGGCTTTCTTGACATTTACCAGGGCGCATCCTTTAATCAGAAGCGAATCGGCATGCATGCCCATCGGG
ATTTTTTCCAGTCATTGATCCGCATGGTTCTTTTCACGGGTTATTCCGCGCTCACACGCTAAGCGGCGCGTCCTGCC
TACAACAAAGGAACAATC**ATGCATCACCATCACCATCACATGGCTACGAAACCCAAAAAACAAAGGGCGTTTCTGT**
TTCCATCGAAGGGAAGCTGCCCAAGATGACCCTGGATATGCCGGTGGACGCGAAAAAGATCAAGGCCATCCAGAAAT
GCCTGGAAAACGGCAAGCTGACCATCACGATGAGCAAGGTGGATCTTGCCGGCGGCCGCATGGGCGAGGGCTATCTC
TACGACTGATGTCCGGCGGCGCTCTGGTTTTCGCCGGCGGCGTCGCGATCTCGCCGCCTCGATGCTCGGGCCTCGGA
GGGAACGTGCCGCCGAACGCGTGCGTTTGGCGCGCGGTCGCGCGATAACGGGCGATGAATTCCGGGCTTCGGCAGTT
CGACCTTAAGGGCGCGGGAAGCCTATGAAAGCCTTCGATCGCGATGCATCGGAGCCTCGGATCGGTCTCGCGTATGG
GCCCGGGATGGCGGAATTCGCCGCACGGAACGCGCACCTCGTCGATTACCGCGCGCTGACCTTCGAATACAACTGGT
CGCCATACTTCCCGCTGTCGGTCCTGACGACGCATATCGAGCGCGCACGCCAGTTGATGGGGCTCGCATGAACGCAG
GGAAAATACACGCCGTGATCGCCGCCGGCGCCAAGGACCCGCGGCTCATCGAGCATTGGCGGCGGCGTCCGGACCTG
CTGCGCCGCGCAGGAATCGAGCCGGATCATCTCGATTTGCGCGTATTGGAGAATTTCGCCGGGTTGAGCCTGAAGGT
GCGTCACAACGGCCTGCGCGGGGATCTGCCGCTGAGTTTCAGGTTGTTGAGCGTGGCCGGCCTCGAGATCGAGGTCT
TCGCCGCGTATGCGTCGGACTGCGCGGAAAGCGGCCATGTATTCGCCGCCACGCCGCGGGAACGCGCGCGCGATCTG
ATGGCGTTCCTCGACAGATGGCTGGACCCCGCGCAACGAAATCACGTTCTGCTGTGGGACATCCTTCGTCATGAATA
TGCGCTTTCGTGCCTGCGGCAAGAAAGCGCCGCGCCGCTACCCGCCGCTCGCCGCATCATCCGCCATCGGGATCGCG
CGACAGGCGGCTGCATTCCGATGCTCAACGGCTCCGTCGCTCAGCACGTGATGCATTGCAATCCGGAAGACCTGGCG
TCGGCGCTGCGTCAGAAGGAGCCGCCGCTCGATGCGGTTCCCGCCGCCGACACTTACTGCTGCTATTGGCGCTCCGC
CGAAACGGGCGAAATCCGGTTTCTGCAGGCCGATACGCTCGGCTATTTCGCGATCGAAGCGATCGACGGCCGGCGAT
CCGCCGCCGACATCTGCGATCGGCTGCTCGGCGGCCGCCGTCTGCTGCCGCAGTTCCTCCGGTCGCTCGACCAACTG
GCCGGAATCGGGATCGTGCGGTTCGAGAGGCCGCTCGGAAGCGCTGCGGCATGAAACTGCTGCTCGTCGACAACCTC
GTCATGCCGGAAGAGGGCTCGCTCGCGTACCTCGACGTGCATCCGCATCTCGGGCTGCTCGCGCTGGCCGCGGTCGC
CGAAGCCGACGGCCATCGCGTGCAGATCTACGATCCGAAACGACTGATCAGGGACGGCACGCTGGCTTACGATGCGA
CGCTTTACGAGCGGGCGAGCCAAGCGATTCTCGCGGCGCGTCCGGACGGGGTCGGCTTCACGGCGCTCGGCTGCAGC
TTTCTGTTTGCGCTCAATGTCGCGGCGCTGTTGAAGCGGCGCGAGCCCGACCTGCCCGTCTTGCTCGGCGGGCCGCA
CGCGACGATGCTGCACCGGCAGATCCTCGAGCGGTTCCCGCAGTTCGACATCATCGTCCGGTACGAGGCGGATGAAA
TCCTGCCCGCCGTGCTCGACTGCCTCCCGCACCGGACATTCGACGTGATTCCCGGCTTGAGCTGGCGCGCGACGGGC
CGCGGCTCGCCGTTGCGCTTCACCGACGGCAAGCCCAAGGTCGAGGATCTGGACCTGCTGCCGATCGCCTCGTACGA
CCATTATCCGGTTGAGGAACTGGGGCTCTCGATGCTGCGCATCGAGGCCGGCCGAGGGTGTCCGTTCGCATGCACAT
TCTGCTCGACGGCCGGTTTCTTTCAACGCAGCTTCCGCCTCAAATCGGCCGAGCGCCTGGTTCGCGAACTGGATATC
CTTCACCAGCGTTATCGCGTGTCGGACTTCAAGCTCGACCACGACATGTTCACCGTGAATCGCCGCAAGGTAATGGA
GTTTTGCGAGGCGGTCGCCGGGCGCGACTATCGGTGGCGCGCGTCGGCGCGGATCGACTGCGTCGACGAAGCATTGT
TGAAGAAGATGGCCGACGCGGGCTGCGTGAATCTTTATTTCGGCGTGGAGACGGGCTCCGAGCGCATGCAAAAGCTC
TGCAAGAAGCGGCTGGATCTGCAACGGGTCGAGCCCATTCTGGCGGCGGCGGATTCATTCGGAATCGAGACGACCGC
GTCGTTCATCACCGGCTATCCCGAGGAAACCGGGGAGGATCAGGACGACACGCTGGACATGATCGGGCGCTGCGCTC

GGCGAGCGTCTTGCCTCACGCAACTGCACATGCTCGCGCCGGAGCCCGGCACACCGCTGTTCGACGAACGCGGCGCG
GAAATCGCCTACGACGGCTGTGGCGGGCCTTACAACACGCGCCTGCTGAGTTCGTCGGACGAGCGTGCGGTGCTCGG
CCATCCCGACATATTCCAAACCTACTATCACTACCCCGCCGCGCTGCCGCGTGCGCGATACATCTTTGCCGTCGGGG
CGGCGGATCTGCTGCGCCGGGTCGGGCCGGTCATTTTCGCGTATGCGTTGCGGGGGTTCGGAGGGCGGCTGAGCACG
CTGATATCGGCGTGGCGACAATTCGCCGACGAAACGTCGCCCGGCGCGCCCGCGGACGCGGCGGGGCTCGAGGCCTT
CATTGCCGCGCGGTTCGGCCGCGGCCATCACCTGCATTCTCTTTTTCGGTACGCGTTGCGGCTCTATGCGGCCCGCG
CTCCGGCGGACGTAATCGTCGAGGGGCCCTATGAGCCGGACCGGCCTTGCATGCTGGCCGAAAACGTCGGTCTGCTG
GAGGACATTCACAATTGCGACGCATTGGTCGACCGGATCAGGCGATCGACCGAAGGCGCGCCGATGCTCGGCGACGC
GGATGTCGGCGGGCTCGGAACATATATCGTTCAGGGGCACGGCGAGGCCGCCACGGGCTATTGGGTGGAGTCCGGCA
TCGCTACGCTTCTCGATCTGTTCAGGTCGCCCCGTTCGTGCCGCGCCGTTGCGCAATGGCTGGCGGACGCCACCCGC
AATGACGGCATAGACGCTTCGATCTTCGAGCCGCTGCTGCGCAGAGGCATCCTGGTGTCGGCCGGTTAAATCCATTT
CGTCCATGGTGCGTCGCACACTCGAACGTTGGTCCGCGCTGGATCTCGCGGAAGGGCTTCAACTGGCGCATGCCGTC
GCCGCGCTGCAGGCGCTCGGCGTTCTGGACGCGATGAGGGAGCCGGTCACGGCGGAAACGTTGAGCGCCAGCCATGA
TCTCGATCCCGAGTTGCTGCGCGGCATCCTCGAATATGCGGCGTCGAGAACGAATCTGGTTCGCAAGACGGACCGCG
GATTCGCGATTTCCGAGCATTACACCGCGCAAGCGCGCTTTCTGTTGAACCTGTACGCCGGCGCTTATGCCGATAAC
GCTTCACGGCTGGCGACGCTGCTCCGGCGTCCGGCATTGGCGGGCTCCATGATCGACCTCGTCGCGCACGCGCGCGC
CTTCGACGCGAGCGAGGCCGGCGGTGGAGCGCTGGCGTCGATCGTCACGCAATTGCGTCTCAATCATGTGCTGGATC
TGGGATGCGGCGCCGGCGCGCTGCTGCGCGAGTTGGCGGCGGACGATCGCGGATTCGTCGGCTGGGGACTGGATCGC
AATCCCGCGATGTGCAAGGCGGCGCGGGTTCGAGCGCGGCAAGCGGGGCTCGCCGCGCGCGTGAAGGTGTTCCACGG
CGACGGCTGGAATCCCGGGGCGTCGGTCCCGCCGCGCGTGCTGGCGCGCGTGCGCAACGTCGTCGCGAGCCAGTTCG
TCAACGAGATGTTTCGCGGCGGCACATCGCGAGTGGAAACGTGGCTTCGGCGCATGCGGCGCTTGCTGCCCGGCCGC
ATGCTCGTGATATCGGACTATTACGGCCGCCTCGGCCACGGCACTCACACGATGCGCCGCGAGACGCTGCTGCATGA
CTATGTGCAGCTCATATCGGGGCAGGGCGTGCCGCCGCCCGATTCCGACGCGTGGTTGGCCATGTATCGAGCAGCCG
GTTGCCGGCCTCTGCACGTGATCGAAGACCGGGCGGCCACGACATGCTTCGTCCATCTGGTGGGGCTATAG

e) Insert from genomic DNA of *B. thailandensis* E264 encoding all proteins, with the gene encoding BthI removed.

ATGAACGGTCTACCTCCGTCGCTTCCGCGGGTCGACAGCACCGAGTCGCTTTTTGCGGAGCCGCTCGCGTTTCTCGC
ACAAGCGCGCTCGCGGCATGGTGATGTCTTCGTCATGCGGGAGCATGGTCCGATTTTTTCCCGCGCGAGCGATTGCA
GCGGCGTGATAGCGGTATTCGGCGAGCATCGGCTTCGACAGATATTGACCGATATAGACAATTTCGCGTTGCCGATG
TCCGCGGCGGCGAAAATGGCATTGCCGAAAAACCTCGTCAACCTCAATCGCGGCTTGCACAGCATGCGCGAGCCGGA
GCATGGCCGGCACAAGCGCCTCTTGACGGGAACGATCAATCGCGAGTTGTTCGACGCGCACCGCTTCGAAATACGAG
CGGCTTTGAACCGCTTTTGCGAAATGCTGAAAGTGGACCGCCGGATTTCAGTGGTCAGCCGGATGCGCGAGCTGACC
GTGGAGATGGCGTCTCATATCTTTCTTGGGGCGCAGTGCCAAGAGGATGACGAACTGGCGTTTCTTTTAAGCGCCTA
TTTTACTTTGCGACGCGAAGCGTCTTCGCTGAACGCGCGCGATCCCCTGCTGTACCGGGACGAACTGATCGGCGTCG
GGCAGCAACTCGACCGCACATTGCGGGAGCGGATCAGGCGGTATCGAAAGCGTCCCGTCGACGCTCGTGCGGGGCTG
CTGCAACGTCTGGCAACGGCCGGACCGCCGGGCTCGCCCGCGCTTTCCGAGGACGAGATAGTCGGCCATGCCAACGT
GATGTTCGTGTCGAGCACCGAGCCCGTCGCGATGTCGCTGACGTGGCTGCTGCTCGTTCTGTCGCAGCTGCCCGATC
TGAGGCGCGCGCTTCGCGCGGAAATTGCCGATCGCGCGTCGATGCCGGCATCGACGAACGGCGCGTCGTGGCTCGAG
AACGTCGTCAACGAGACGCTCCGGCTCCTGACGCCCAACGCGCTGATGGTTCGCGCGACGACGCGCGCGGTCTCGCT
GCAAGGCGTCGCGCTTCCGGCGCGTTGCGAGATCGTCGTGTGTCCGTTCCTCGCGCATCGCGAAGCCAAGCCGTTTC
CGGACCCGCATGCATTCTCGCCGTCCCGGTGGGAGACGGCCAGGCCGTCTCCGTACGAGTATTTTCCGTTCGGCGCC
GGCGGTCATTTTTGCGCGGGGCGAAATCTCGCGCTCTCGCTGATTCGCGAAGTACTGTCAACGCTGCTATCGCGCTT
CGATTTCGTTTTGGATGGCGAACAGTCTATCGACTGGCGCATTCATATCATGTTGATGCCGAAAGGCGACCCGGCAC
TCATCGCGCATCCCGTCGATGAGCGCGGTGACACGCCGTCGCCGAAATGGCGCGGGCCGATAACCGATCTATTCCAT
TTCGCGCCGGGGCTTTCTTGACATTTACCAGGGCGCATCCTTTAATCAGAAGCGAATCGGCATGCATGCCCATCGGG
ATTTTTTCCAGTCATTGATCCGCATGGTTCTTTTCACGGGTTATTCCGCGCTCACACGCTAAGCGGCGCGTCCTGCC
TACAACAAAGGAACAATC**ATGCATCACCATCACCATCACATGGCTACGAAACCCAAAAAAACAAAGGGCGTTTCTGT**
TTCCATCGAAGGGAAGCTGCCCAAGATGACCCTGGATATGCCGGTGGACGCGAAAAAGATCAAGGCCATCCAGAAAT
GCCTGGAAAACGGCAAGCTGACCATCACGATGAGCAAGGTGGATCTTGCCGGCGGCCGCATGGGCGAGGGCTATCTC
TACGACTGATGTCCGGCGGCGCTCTGGTTTTCGCCGGCGGCGTCGCGATCTCGCCGCCTCGATGCTCGGGCCTCGGA
GGGAACGTGCCGCCGAACGCGTGCGTTTGGCGCGCGGTCGCGCGATAACGGGCGATGAATTCCGGGCTTCGGCAGTT
CGACCTTAAGGGCGCGGGAAGCCTATGAAAGCCTTCGATCGCGATGCATCGGAGCCTCGGATCGGTCTCGCGTATGG
GCCCGGGATGGCGGAATTCGCCGCACGGAACGCGCACCTCGTCGATTACATCGAAGTGCCGTTCGAGCAATTGCGAT
TCTCGCCGGCGGTCGCCGAGCTTCAGCAAACCATTCCGTTCGTTCTGCACTGCGCCAGCTTGTCCGTCGCGGGATTC

GTGCCGCCCGACGATTCGACGGTCGACGCAATCGAGCGCACGGCCGTGCAGACCGGCACGCCATGGATAGGCGAACA
TCTCGCGTACATTTCGGCCGATCCCGTTGGCGAAGCGCTGGGCGGACCGGTGAGCCCACCTCGTTGTCGTACACGT
TGTGCCCTCAGTTGAGCGACGAGACGGTACGCCGCGTCGTCGACAATCTCGCCGCGCTTCGTCCGCATTTTCCGGTT
CCACTGATCGTCGAAAACTCCCCGCAGTACTTTCCGATACCGGGCAGCACGATGGGAATGACGGACTTCATTCGCGC
GATCACGGACCGATGCGACGTCGGTTTGCTCCTGGACCTGAGTCACTTCCTGATCACGGCGCACAACACGGGCGCAG
AGGTGCATCGGGAACTCGCCCGGCTTCCGCTGGAGCGCGTGGTCGAAGTTCATCTGTCCGGAATGAGCGTGCAGTCC
GGCACGGCCTGGGACGACCATTCGCTGCCGGCATCGCCGATCCTGTTCGAACTGCTCGAGCGCCTGCTCGACGTGGC
GCGGCCTCGCGCGCTGACCTTCGAATACAACTGGTCGCCATACTTCCCGCTGTCGGTCCTGACGACGCATATCGAGC
GCGCACGCCAGTTGATGGGGCTCGCATGAAACGCAGGGAAAATACACGCCGTGATCGCCGCCGGCGCCAAGGACCCG
CGGCTCATCGAGCATTGGCGGCGGCGTCCGGACCTGCTGCGCCGCGCAGGAATCGAGCCGGATGATCGGCTGCTCGG
CGGCCGCCGTCTGCTGCCGCAGTTCCTCCGGTCGCTCGACCAACTGGCCGGAATCGGGATCGTGCGGTTCGAGAGGC
CGCTCGGAAGCGCTGCGGCATGAAACTGCTGCTCGTCGACAACCTCGTCATGCCGGAAGAGGGCTCGCTCGCGTACC
TCGACGTGCATCCGCATCTCGGGCTGCTCGCGCTGGCCGCGGTCGCCGAAGCCGACGGCCATCGCGTGCAGATCTAC
GATCCGAAACGACTGATCAGGGACGGCACGCTGGCTTACGATGCGACGCTTTACGAGCGGGCGAGCCAAGCGATTCT
CGCGGCGCGTCCGGACGGGGTCGGCTTCACGGCGCTCGGCTGCAGCTTTCTGTTTGCGCTCAATGTCGCGGCGCTGT
TGAAGCGGCGCGAGCCCGACCTGCCCGTCTTGCTCGGCGGGCCGCACGCGACGATGCTGCACCGGCAGATCCTCGAG
CGGTTCCCGCAGTTCGACATCATCGTCCGGTACGAGGCGGATGAAATCCTGCCCGCCGTGCTCGACTGCCTCCCGCA
CCGGACATTCGACGTGATTCCCGGCTTGAGCTGGCGCGCGACGGGCCGCGGCTCGCCGTTGCGCTTCACCGACGGCA
AGCCCAAGGTCGAGGATCTGGACCTGCTGCCGATCGCCTCGTACGACCATTATCCGGTTGAGGAACTGGGGCTCTCG
ATGCTGCGCATCGAGGCCGGCCGAGGGTGTCCGTTCGCATGCACATTCTGCTCGACGGCCGGTTTCTTTCAACGCAG
CTTCCGCCTCAAATCGGCCGAGCGCCTGGTTCGCGAACTGGATATCCTTCACCAGCGTTATCGCGTGTCGGACTTCA
AGCTCGACCACGACATGTTCACCGTGAATCGCCGCAAGGTAATGGAGTTTTGCGAGGCGGTCGCCGGGCGCGACTAT
CGGTGGCGCGCGTCGGCGCGGATCGACTGCGTCGACGAAGCATTGTTGAAGAAGATGGCCGACGCGGGCTGCGTGAA
TCTTTATTTCGGCGTGGAGACGGGCTCCGAGCGCATGCAAAAGCTCTGCAAGAAGCGGCTGGATCTGCAACGGGTCG
AGCCCATTCTGGCGGCGGCGGATTCATTCGGAATCGAGACGACCGCGTCGTTCATCACCGGCTATCCCGAGGAAACC
GGGGAGGATCAGGACGACACGCTGGACATGATCGGGCGCTGCGCTCGGCGAGCGTCTTGCCTCACGCAACTGCACAT
GCTCGCGCCGGAGCCCGGCACACCGCTGTTCGACGAACGCGGCGCGGAAATCGCCTACGACGGCTGTGGCGGGCCTT
ACAACACGCGCCTGCTGAGTTCGTCGGACGAGCGTGCGGTGCTCGGCCATCCCGACATATTCCAAACCTACTATCAC
TACCCCGCCGCGCTGCCGCGTGCGCGATACATCTTTGCCGTCGGGGCGGCGGATCTGCTGCGCCGGGTCGGGCCGGT
CATTTTCGCGTATGCGTTGCGGGGGTTCGGAGGGCGGCTGAGCACGCTGATATCGGCGTGGCGACAATTCGCCGACG
AAACGTCGCCCGGCGCGCCCGCGGACGCGGCGGGGCTCGAGGCCTTCATTGCCGCGCGGTTCGGCCGCGGCCATCAC
CTGCATTCTCTTTTTCGGTACGCGTTGCGGCTCTATGCGGCCCGCGCTCCGGCGGACGTAATCGTCGAGGGGCCCTA
TGAGCCGGACCGGCCTTGCATGCTGGCCGAAAACGTCGGTCTGCTGGAGGACATTCACAATTGCGACGCATTGGTCG
ACCGGATCAGGCGATCGACCGAAGGCGCGCCGATGCTCGGCGACGCGGATGTCGGCGGGCTCGGAACATATATCGTT
CAGGGGCACGGCGAGGCCGCCACGGGCTATTGGGTGGAGTCCGGCATCGCTACGCTTCTCGATCTGTTCAGGTCGCC
CCGTTCGTGCCGCGCCGTTGCGCAATGGCTGGCGGACGCCACCCGCAATGACGGCATAGACGCTTCGATCTTCGAGC
CGCTGCTGCGCAGAGGCATCCTGGTGTCGGCCGGTTAAATCCATTTCGTCCATGGTGCGTCGCACACTCGAACGTTG
GTCCGCGCTGGATCTCGCGGAAGGGCTTCAACTGGCGCATGCCGTCGCCGCGCTGCAGGCGCTCGGCGTTCTGGACG
CGATGAGGGAGCCGGTCACGGCGGAAACGTTGAGCGCCAGCCATGATCTCGATCCCGAGTTGCTGCGCGGCATCCTC
GAATATGCGGCGTCGAGAACGAATCTGGTTCGCAAGACGGACCGCGGATTCGCGATTTCCGAGCATTACACCGCGCA
AGCGCGCTTTCTGTTGAACCTGTACGCCGGCGCTTATGCCGATAACGCTTCACGGCTGGCGACGCTGCTCCGGCGTC
CGGCATTGGCGGGCTCCATGATCGACCTCGTCGCGCACGCGCGCGCCTTCGACGCGAGCGAGGCCGGCGGTGGAGCG
CTGGCGTCGATCGTCACGCAATTGCGTCTCAATCATGTGCTGGATCTGGGATGCGGCGCCGGCGCGCTGCTGCGCGA
GTTGGCGGCGGACGATCGCGGATTCGTCGGCTGGGGACTGGATCGCAATCCCGCGATGTGCAAGGCGGCGCGGGTTC
GAGCGCGGCAAGCGGGGCTCGCCGCGCGCGTGAAGGTGTTCCACGGCGACGGCTGGAATCCCGGGGCGTCGGTCCCG
CCGCGCGTGCTGGCGCGCGTGCGCAACGTCGTCGCGAGCCAGTTCGTCAACGAGATGTTTCGCGGCGGCACATCGCG
AGTGGAAACGTGGCTTCGGCGCATGCGGCGCTTGCTGCCCGGCCGCATGCTCGTGATATCGGACTATTACGGCCGCC
TCGGCCACGGCACTCACACGATGCGCCGCGAGACGCTGCTGCATGACTATGTGCAGCTCATATCGGGGCAGGGCGTG
CCGCCGCCCGATTCCGACGCGTGGTTGGCCATGTATCGAGCAGCCGGTTGCCGGCCTCTGCACGTGATCGAAGACCG
GGCGGCCACGACATGCTTCGTCCATCTGGTGGGGCTATAG

f) Insert from genomic DNA of *B. thailandensis* E264 encoding all proteins, with the gene encoding BthS removed.

ATGAACGGTCTACCTCCGTCGCTTCCGCGGGTCGACAGCACCGAGTCGCTTTTTGCGGAGCCGCTCGCGTTTCTCGC
ACAAGCGCGCTCGCGGCATGGTGATGTCTTCGTCATGCGGGAGCATGGTCCGATTTTTTCCCGCGCGAGCGATTGCA

GCGGCGTGATAGCGGTATTCGGCGAGCATCGGCTTCGACAGATATTGACCGATATAGACAATTTCGCGTTGCCGATG
TCCGCGGCGGCGAAAATGGCATTGCCGAAAAACCTCGTCAACCTCAATCGCGGCTTGCACAGCATGCGCGAGCCGGA
GCATGGCCGGCACAAGCGCCTCTTGACGGGAACGATCAATCGCGAGTTGTTCGACGCGCACCGCTTCGAAATACGAG
CGGCTTTGAACCGCTTTTGCGAAATGCTGAAAGTGGACCGCCGGATTTCAGTGGTCAGCCGGATGCGCGAGCTGACC
GTGGAGATGGCGTCTCATATCTTTCTTGGGGCGCAGTGCCAAGAGGATGACGAACTGGCGTTTCTTTTAAGCGCCTA
TTTTACTTTGCGACGCGAAGCGTCTTCGCTGAACGCGCGCGATCCCCTGCTGTACCGGGACGAACTGATCGGCGTCG
GGCAGCAACTCGACCGCACATTGCGGGAGCGGATCAGGCGGTATCGAAAGCGTCCCGTCGACGCTCGTGCGGGGCTG
CTGCAACGTCTGGCAACGGCCGGACCGCCGGGCTCGCCCGCGCTTTCCGAGGACGAGATAGTCGGCCATGCCAACGT
GATGTTCGTGTCGAGCACCGAGCCCGTCGCGATGTCGCTGACGTGGCTGCTGCTCGTTCTGTCGCAGCTGCCCGATC
TGAGGCGCGCGCTTCGCGCGGAAATTGCCGATCGCGCGTCGATGCCGGCATCGACGAACGGCGCGTCGTGGCTCGAG
AACGTCGTCAACGAGACGCTCCGGCTCCTGACGCCCAACGCGCTGATGGTTCGCGCGACGACGCGCGCGGTCTCGCT
GCAAGGCGTCGCGCTTCCGGCGCGTTGCGAGATCGTCGTGTGTCCGTTCCTCGCGCATCGCGAAGCCAAGCCGTTTC
CGGACCCGCATGCATTCTCGCCGTCCCGGTGGGAGACGGCCAGGCCGTCTCCGTACGAGTATTTTCCGTTCGGCGCC
GGCGGTCATTTTTGCGCGGGGCGAAATCTCGCGCTCTCGCTGATTCGCGAAGTACTGTCAACGCTGCTATCGCGCTT
CGATTTCGTTTTGGATGGCGAACAGTCTATCGACTGGCGCATTCATATCATGTTGATGCCGAAAGGCGACCCGGCAC
TCATCGCGCATCCCGTCGATGAGCGCGGTGACACGCCGTCGCCGAAATGGCGCGGGCCGATAACCGATCTATTCCAT
TTCGCGCCGGGGCTTTCTTGACATTTACCAGGGCGCATCCTTTAATCAGAAGCGAATCGGCATGCATGCCCATCGGG
ATTTTTTCCAGTCATTGATCCGCATGGTTCTTTTCACGGGTTATTCCGCGCTCACACGCTAAGCGGCGCGTCCTGCC
TACAACAAAGGAACAATC**ATGCATCACCATCACCATCACATGGCTACGAAACCCAAAAAAACAAAGGGCGTTTCTGT**
TTCCATCGAAGGGAAGCTGCCCAAGATGACCCTGGATATGCCGGTGGACGCGAAAAAGATCAAGGCCATCCAGAAAT
GCCTGGAAAACGGCAAGCTGACCATCACGATGAGCAAGGTGGATCTTGCCGGCGGCCGCATGGGCGAGGGCTATCTC
TACGACTGATGTCCGGCGGCGCTCTGGTTTTCGCCGGCGGCGTCGCGATCTCGCCGCCTCGATGCTCGGGCCTCGGA
GGGAACGTGCCGCCGAACGCGTGCGTTTGGCGCGCGGTCGCGCGATAACGGGCGATGAATTCCGGGCTTCGGCAGTT
CGACCTTAAGGGCGCGGGAAGCCTATGAAAGCCTTCGATCGCGATGCATCGGAGCCTCGGATCGGTCTCGCGTATGG
GCCCGGGATGGCGGAATTCGCCGCACGGAACGCGCACCTCGTCGATTACATCGAAGTGCCGTTCGAGCAATTGCGAT
TCTCGCCGGCGGTCGCCGAGCTTCAGCAAACCATTCCGTTCGTTCTGCACTGCGCCAGCTTGTCCGTCGCGGGATTC
GTGCCGCCCGACGATTCGACGGTCGACGCAATCGAGCGCACGGCCGTGCAGACCGGCACGCCATGGATAGGCGAACA
TCTCGCGTACATTTCGGCCGATCCCGTTGGCGAAGCGCTGGGCGGACCGGTGAGCCCACCTCGTTGTCGTACACGT
TGTGCCCTCAGTTGAGCGACGAGACGGTACGCCGCGTCGTCGACAATCTCGCCGCGCTTCGTCCGCATTTTCCGGTT
CCACTGATCGTCGAAAACTCCCCGCAGTACTTTCCGATACCGGGCAGCACGATGGGAATGACGGACTTCATTCGCGC
GATCACGGACCGATGCGACGTCGGTTTGCTCCTGGACCTGAGTCACTTCCTGATCACGGCGCACAACACGGGCGCAG
AGGTGCATCGGGAACTCGCCCGGCTTCCGCTGGAGCGCGTGGTCGAAGTTCATCTGTCCGGAATGAGCGTGCAGTCC
GGCACGGCCTGGGACGACCATTCGCTGCCGGCATCGCCGATCCTGTTCGAACTGCTCGAGCGCCTGCTCGACGTGGC
GCGGCCTCGCGCGCTGACCTTCGAATACAACTGGTCGCCATACTTCCCGCTGTCGGTCCTGACGACGCATATCGAGC
GCGCACGCCAGTTGATGGGGCTCGCATGAACGCAGGGAAAATACACGCCGTGATCGCCGCCGGCGCCAAGGACCCGC
GGCTCATCGAGCATTGGCGGCGGCGTCCGGACCTGCTGCGCCGCGCAGGAATCGAGCCGGATCATCTCGATTTGCGC
GTATTGGAGAATTTCGCCGGGTTGAGCCTGAAGGTGCGTCACAACGGCCTGCGCGGGGATCTGCCGCTGAGTTTCAG
GTTGTTGAGCGTGGCCGGCCTCGAGATCGAGGTCTTCGCCGCGTATGCGTCGGACTGCGCGGAAAGCGGCCATGTAT
TCGCCGCCACGCCGCGGGAACGCGCGCGCGATCTGATGGCGTTCCTCGACAGATGGCTGGACCCCGCGCAACGAAAT
CACGTTCTGCTGTGGGACATCCTTCGTCATGAATATGCGCTTTCGTGCCTGCGGCAAGAAAGCGCCGCGCCGCTACC
CGCCGCTCGCCGCATCATCCGCCATCGGGATCGCGCGACAGGCGGCTGCATTCCGATGCTCAACGGCTCCGTCGCTC
AGCACGTGATGCATTGCAATCCGGAAGACCTGGCGTCGGCGCTGCGTCAGAAGGAGCCGCCGCTCGATGCGGTTCCC
GCCGCCGACACTTACTGCTGCTATTGGCGCTCCGCCGAAACGGGCGAAATCCGGTTTCTGCAGGCCGATACGCTCGG
CTATTTCGCGATCGAAGCGATCGACGGCCGGCGATCCGCCGCCGACATCTGCGATCGGCTGCTCGGCGGCCGCCGTC
TGCTGCCGCAGTTCCTCCGGTCGCTCGACCAACTGGCCGGAATCGGGATCGTGCGGTTCGAGAGGCCGCTCGGAAGC
GCTGCGGCATGAAACTGCTGCTCGTCGACAACCTCGTCATGCCGGAAGAGGGCTCGCTCGCGTACCTCGACGTGCAT
CCGCATCTCGGGCTGCTCGCGCTGGCCGCGGTCGCCGAAGCCGACGGCCATCGCGTGCAGATCTACGATCCGAAACG
ACTGATCAGGGACGGCACGCTGGCTTACGATGCGACGCTTTACGAGCGGGCGAGCCAAGCGATTCTCGCGGCGCGTC
CGGACGGGGTCGGCTTCACGGCGCTCGGCTGCAGCTTTCTGTTTGCGCTCAATGTCGCGGCGCTGTTGAAGCGGCGC
GAGCCCGACCTGCCCGTCTTGCTCGGCGGGCCGCACGCGACGATGCTGCACCGGCAGATCCTCGAGCGGTTCCCGCA
GTTCGACATCATCGTCCGGTACGAGGCGGATGAAATCCTGCCCGCCGTGCTCGACTGCCTCCCGCACCGGACATTCG
ACGTGATTCCCGGCTTGAGCTGGCGCGCGACGGGCCGCGGCTCGCCGTTGCGCTTCACCGACGGCAAGCCCAAGGTC
GAGGATCTGGACCTGCTGCCGATCGCCTCGTACGACCATTATCCGGTTGAGGAACTGGGGCTCTCGATGCTGCGCAT
CGAGGCCGGCCGAGGGTGTCCGTTCGCATGCACATTCTGCTCGACGGCCGGTTTCTTTCAACGCAGCTTCCGCCTCA
AATCGGCCGAGCGCCTGGTTCGCGAACTGGATATCCTTCACCAGCGTTATCGCGTGTCGGACTTCAAGCTCGACCAC
GACATGTTCACCGTGAATCGCCGCAAGGTAATGGAGTTTTGCGAGGCGGTCGCCGGGCGCGACTATCGGTGGCGCGC
GTCGGCGCGGATCGACTGCGTCGACGAAGCATTGTTGAAGAAGATGGCCGACGCGGGCTGCGTGAATCTTTATTTCG

GCGTGGAGACGGGCTCCGAGCGCATGCAAAAGCTCTGCAAGAAGCGGCTGGATCTGCAACGGGTCGAGCCCATTCTG
GCGGCGGCGGATTCATTCGGAATCGAGACGACCGCGTCGTTCATCACCGGCTATCCCGAGGAAACCGGGGAGGATCA
GGACGACACGCTGGACATGATCGGGCGCTGCGCTCGGCGAGCGTCTTGCCTCACGCAACTGCACATGCTCGCGCCGG
AGCCCGGCACACCGCTGTTCGACGAACGCGGCGCGGAAATCGCCTACGACGGCTGTGGCGGGCCTTACAACACGCGC
CTGCTGAGTTCGTCGGACGAGCGTGCGGTGCTCGGCCATCCCGACATATTCCAAACCTACTATCACTACCCCGCCGC
GCTGCCGCGTGCGCGATACATCTTTGCCGTCGGGGCGGCGGATCTGCTGCGCCGGGTCGGGCCGGTCATTTTCGCGT
ATGCGTTGCGGGGGTTCGGAGGGCGGCTGAGCACGCTGATATCGGCGTGGCGACAATTCGCCGACGAAACGTCGCCC
GGCGCGCCCGCGGACGCGGCGGGGCTCGAGGCCTTCATTGCCGCGCGGTTCGGCCGCGGCCATCACCTGCATTCTCT
TTTTCGGTACGCGTTGCGGCTCTATGCGGCCCGCGCTCCGGCGGACGTAATCGTCGAGGGGCCCTATGAGCCGGACC
GGCCTTGCATGCTGGCCGAAAACGTCGGTCTGCTGGAGGACATTCACAATTGCGACGCATTGGTCGACCGGATCAGG
CGATCGACCGAAGGCGCGCCGATGCTCGGCGACGCGGATGTCGGCGGGCTCGGAACATATATCGTTCAGGGGCACGG
CGAGGCCGCCACGGGCTATTGGGTGGAGTCCGGCATCGCTACGCTTCTCGATCTGTTCAGGTCGCCCCGTTCGTGCC
GCGCCGTTGCGCAATGGCTGGCGGACGCCACCCGCAATGACGGCATAGACGCTTCGATCTTCGAGCCGCTGCTGCGC
AGAGGCATCCTGGTGTCGGCCGGTTAAATCCATTTCGTCCATGGTGCGTCGCACACTCGAACGTTGGTCCGCGCTGG
ATCTCGCGGAAGGGCTTCAACTGGCGCATGCCGTCGCCGCGCTGCAGGCGCTCGGCGTTCTGGACTAG

Table S3: Sequence of the reconstructed pSCrhaB2-based vectors used in this study. Each gene is highlighted as follows. *bthA*: bolded black; *bthO*: pink; *bthD*: green; *bthH*: orange; *bthI*: underlined; *bthS*: blue. The N-terminus of BthA contains a hexa-His tag. In this construct, BthD is placed right after the ribosome binding site downstream of the rhamnose-inducible promoter in the pSCrhaB2 backbone.

ATGAAACTGCTGCTCGTCGACAACCTCGTCATGCCGGAAGAGGGCTCGCTCGCGTACCTCGACGTGCATCCGCATCT
CGGGCTGCTCGCGCTGGCCGCGGTCGCCGAAGCCGACGGCCATCGCGTGCAGATCTACGATCCGAAACGACTGATCA
GGGACGGCACGCTGGCTTACGATGCGACGCTTTACGAGCGGGCGAGCCAAGCGATTCTCGCGGCGCGTCCGGACGGG
GTCGGCTTCACGGCGCTCGGCTGCAGCTTTCTGTTTGCGCTCAATGTCGCGGCGCTGTTGAAGCGGCGCGAGCCCGA
CCTGCCCGTCTTGCTCGGCGGGCCGCACGCGACGATGCTGCACCGGCAGATCCTCGAGCGGTTCCCGCAGTTCGACA
TCATCGTCCGGTACGAGGCGGATGAAATCCTGCCCGCCGTGCTCGACTGCCTCCCGCACCGGACATTCGACGTGATT
CCCGGCTTGAGCTGGCGCGCGACGGGCCGCGGCTCGCCGTTGCGCTTCACCGACGGCAAGCCCAAGGTCGAGGATCT
GGACCTGCTGCCGATCGCCTCGTACGACCATTATCCGGTTGAGGAACTGGGGCTCTCGATGCTGCGCATCGAGGCCG
GCCGAGGGTGTCCGTTCGCATGCACATTCTGCTCGACGGCCGGTTTCTTTCAACGCAGCTTCCGCCTCAAATCGGCC
GAGCGCCTGGTTCGCGAACTGGATATCCTTCACCAGCGTTATCGCGTGTCGGACTTCAAGCTCGACCACGACATGTT
CACCGTGAATCGCCGCAAGGTAATGGAGTTTTGCGAGGCGGTCGCCGGGCGCGACTATCGGTGGCGCGCGTCGGCGC
GGATCGACTGCGTCGACGAAGCATTGTTGAAGAAGATGGCCGACGCGGGCTGCGTGAATCTTTATTTCGGCGTGGAG
ACGGGCTCCGAGCGCATGCAAAAGCTCTGCAAGAAGCGGCTGGATCTGCAACGGGTCGAGCCCATTCTGGCGGCGGC
GGATTCATTCGGAATCGAGACGACCGCGTCGTTCATCACCGGCTATCCCGAGGAAACCGGGGAGGATCAGGACGACA
CGCTGGACATGATCGGGCGCTGCGCTCGGCGAGCGTCTTGCCTCACGCAACTGCACATGCTCGCGCCGGAGCCCGGC
ACACCGCTGTTCGACGAACGCGGCGCGGAAATCGCCTACGACGGCTGTGGCGGGCCTTACAACACGCGCCTGCTGAG
TTCGTCGGACGAGCGTGCGGTGCTCGGCCATCCCGACATATTCCAAACCTACTATCACTACCCCGCCGCGCTGCCGC
GTGCGCGATACATCTTTGCCGTCGGGGCGGCGGATCTGCTGCGCCGGGTCGGGCCGGTCATTTTCGCGTATGCGTTG
CGGGGGTTCGGAGGGCGGCTGAGCACGCTGATATCGGCGTGGCGACAATTCGCCGACGAAACGTCGCCCGGCGCGCC
CGCGGACGCGGCGGGGCTCGAGGCCTTCATTGCCGCGCGGTTCGGCCGCGGCCATCACCTGCATTCTCTTTTTCGGT
ACGCGTTGCGGCTCTATGCGGCCCGCGCTCCGGCGGACGTAATCGTCGAGGGGCCCTATGAGCCGGACCGGCCTTGC
ATGCTGGCCGAAAACGTCGGTCTGCTGGAGGACATTCACAATTGCGACGCATTGGTCGACCGGATCAGGCGATCGAC
CGAAGGCGCGCCGATGCTCGGCGACGCGGATGTCGGCGGGCTCGGAACATATATCGTTCAGGGGCACGGCGAGGCCG
CCACGGGCTATTGGGTGGAGTCCGGCATCGCTACGCTTCTCGATCTGTTCAGGTCGCCCCGTTCGTGCCGCGCCGTT
GCGCAATGGCTGGCGGACGCCACCCGCAATGACGGCATAGACGCTTCGATCTTCGAGCCGCTGCTGCGCAGAGGCAT
CCTGGTGTCGGCCGGTTAAATAGCAGGAGGAATTCACCATGAACGGTCTACCTCCGTCGCTTCCGCGGGTCGACAGC
ACCGAGTCGCTTTTTGCGGAGCCGCTCGCGTTTCTCGCACAAGCGCGCTCGCGGCATGGTGATGTCTTCGTCATGCG
GGAGCATGGTCCGATTTTTTCCCGCGCGAGCGATTGCAGCGGCGTGATAGCGGTATTCGGCGAGCATCGGCTTCGAC
AGATATTGACCGATATAGACAATTTCGCGTTGCCGATGTCCGCGGCGGCGAAAATGGCATTGCCGAAAAACCTCGTC
AACCTCAATCGCGGCTTGCACAGCATGCGCGAGCCGGAGCATGGCCGGCACAAGCGCCTCTTGACGGGAACGATCAA
TCGCGAGTTGTTCGACGCGCACCGCTTCGAAATACGAGCGGCTTTGAACCGCTTTTGCGAAATGCTGAAAGTGGACC
GCCGGATTTCAGTGGTCAGCCGGATGCGCGAGCTGACCGTGGAGATGGCGTCTCATATCTTTCTTGGGGCGCAGTGC
CAAGAGGATGACGAACTGGCGTTTCTTTTAAGCGCCTATTTTACTTTGCGACGCGAAGCGTCTTCGCTGAACGCGCG
CGATCCCCTGCTGTACCGGGACGAACTGATCGGCGTCGGGCAGCAACTCGACCGCACATTGCGGGAGCGGATCAGGC
GGTATCGAAAGCGTCCCGTCGACGCTCGTGCGGGGCTGCTGCAACGTCTGGCAACGGCCGGACCGCCGGGCTCGCCC
GCGCTTTCCGAGGACGAGATAGTCGGCCATGCCAACGTGATGTTCGTGTCGAGCACCGAGCCCGTCGCGATGTCGCT
GACGTGGCTGCTGCTCGTTCTGTCGCAGCTGCCCGATCTGAGGCGCGCGCTTCGCGCGGAAATTGCCGATCGCGCGT
CGATGCCGGCATCGACGAACGGCGCGTCGTGGCTCGAGAACGTCGTCAACGAGACGCTCCGGCTCCTGACGCCCAAC
GCGCTGATGGTTCGCGCGACGACGCGCGCGGTCTCGCTGCAAGGCGTCGCGCTTCCGGCGCGTTGCGAGATCGTCGT
GTGTCCGTTCCTCGCGCATCGCGAAGCCAAGCCGTTTCCGGACCCGCATGCATTCTCGCCGTCCCGGTGGGAGACGG
CCAGGCCGTCTCCGTACGAGTATTTTCCGTTCGGCGCCGGCGGTCATTTTTGCGCGGGGCGAAATCTCGCGCTCTCG
CTGATTCGCGAAGTACTGTCAACGCTGCTATCGCGCTTCGATTTCGTTTTGGATGGCGAACAGTCTATCGACTGGCG
CATTCATATCATGTTGATGCCGAAAGGCGACCCGGCACTCATCGCGCATCCCGTCGATGAGCGCGGTGACACGCCGT
CGCCGAAATGGCGCGGGCCGATAACCGATCTATTCCATTTCGCGCCGGGGCTTTCTTGACATTTACCAGGGCGCATC
CTTTAATCAGAAGCGAATCGGCATGCATGCCCATCGGGATTTTTTCCAGTCATTGATCCGCATGGTTCTTTTCACGG
GTTATTCCGCGCTCACACGCTAAGCGGCGCGTCCTGCCTACAACAAAGGAACAATC**ATGCATCACCATCACCATCAC**
ATGGCTACGAAACCCAAAAAAACAAAGGGCGTTTCTGTTTCCATCGAAGGGAAGCTGCCCAAGATGACCCTGGATAT
GCCGGTGGACGCGAAAAAGATCAAGGCCATCCAGAAATGCCTGGAAAACGGCAAGCTGACCATCACGATGAGCAAGG
TGGATCTTGCCGGCGGCCGCATGGGCGAGGGCTATCTCTACGACTGATGTCCGGCGGCGCTCTGGTTTTCGCCGGCG
GCGTCGCGATCTCGCCGCCTCGATGCTCGGGCCTCGGAGGGAACGTGCCGCCGAACGCGTGCGTTTGGCGCGCGGTC

GCGCGATAACGGGCGATGAATTCCGGGCTTCGGCAGTTCGACCTTAAGGGCGCGGGAAGCCTATGAAAGCCTTCGAT
CGCGATGCATCGGAGCCTCGGATCGGTCTCGCGTATGGGCCCGGGATGGCGGAATTCGCCGCACGGAACGCGCACCT
CGTCGATTACATCGAAGTGCCGTTCGAGCAATTGCGATTCTCGCCGGCGGTCGCCGAGCTTCAGCAAACCATTCCGT
TCGTTCTGCACTGCGCCAGCTTGTCCGTCGCGGGATTCGTGCCGCCCGACGATTCGACGGTCGACGCAATCGAGCGC
ACGGCCGTGCAGACCGGCACGCCATGGATAGGCGAACATCTCGCGTACATTTCGGCCGATCCCGTTGGCGAAGCGCT
GGGCGGGACCGGTGAGCCCACCTCGTTGTCGTACACGTTGTGCCCTCAGTTGAGCGACGAGACGGTACGCCGCGTCG
TCGACAATCTCGCCGCGCTTCGTCCGCATTTTCCGGTTCCACTGATCGTCGAAAACTCCCCGCAGTACTTTCCGATA
CCGGGCAGCACGATGGGAATGACGGACTTCATTCGCGCGATCACGGACCGATGCGACGTCGGTTTGCTCCTGGACCT
GAGTCACTTCCTGATCACGGCGCACAACACGGGCGCAGAGGTGCATCGGGAACTCGCCCGGCTTCCGCTGGAGCGCG
TGGTCGAAGTTCATCTGTCCGGAATGAGCGTGCAGTCCGGCACGGCCTGGGACGACCATTCGCTGCCGGCATCGCCG
ATCCTGTTCGAACTGCTCGAGCGCCTGCTCGACGTGGCGCGGCCTCGCGCGCTGACCTTCGAATACAACTGGTCGCC
ATACTTCCCGCTGTCGGTCCTGACGACGCATATCGAGCGCGCACGCCAGTTGATGGGGCTCGCATGAACGCAGGGAA
AATACACGCCGTGATCGCCGCCGGCGCCAAGGACCCGCGGCTCATCGAGCATTGGCGGCGGCGTCCGGACCTGCTGC
GCCGCGCAGGAATCGAGCCGGATCATCTCGATTTGCGCGTATTGGAGAATTTCGCCGGGTTGAGCCTGAAGGTGCGT
CACAACGGCCTGCGCGGGGATCTGCCGCTGAGTTTCAGGTTGTTGAGCGTGGCCGGCCTCGAGATCGAGGTCTTCGC
CGCGTATGCGTCGGACTGCGCGGAAAGCGGCCATGTATTCGCCGCCACGCCGCGGGAACGCGCGCGCGATCTGATGG
CGTTCCTCGACAGATGGCTGGACCCCGCGCAACGAAATCACGTTCTGCTGTGGGACATCCTTCGTCATGAATATGCG
CTTTCGTGCCTGCGGCAAGAAAGCGCCGCGCCGCTACCCGCCGCTCGCCGCATCATCCGCCATCGGGATCGCGCGAC
AGGCGGCTGCATTCCGATGCTCAACGGCTCCGTCGCTCAGCACGTGATGCATTGCAATCCGGAAGACCTGGCGTCGG
CGCTGCGTCAGAAGGAGCCGCCGCTCGATGCGGTTCCCGCCGCCGACACTTACTGCTGCTATTGGCGCTCCGCCGAA
ACGGGCGAAATCCGGTTTCTGCAGGCCGATACGCTCGGCTATTTCGCGATCGAAGCGATCGACGGCCGGCGATCCGC
CGCCGACATCTGCGATCGGCTGCTCGGCGGCCGCCGTCTGCTGCCGCAGTTCCTCCGGTCGCTCGACCAACTGGCCG
GAATCGGGATCGTGCGGTTCGAGAGGCCGCTCGGAAGCGCTGCGGCATGAAACTGCTGCTCGTCGACAACCTCGTCA
TGCCGGAAGAGGGCTCGCTCGCGTACCTCGACGTGCATGCCGTTGCGCAATGGCTGGCGGACGCCACCCGCAATGAC
GGCATAGACGCTTCGATCTTCGAGCCGCTGCTGCGCAGAGGCATCCTGGTGTCGGCCGGTTAAATCCATTTCGTCCA
TGGTGCGTCGCACACTCGAACGTTGGTCCGCGCTGGATCTCGCGGAAGGGCTTCAACTGGCGCATGCCGTCGCCGCG
CTGCAGGCGCTCGGCGTTCTGGACGCGATGAGGGAGCCGGTCACGGCGGAAACGTTGAGCGCCAGCCATGATCTCGA
TCCCGAGTTGCTGCGCGGCATCCTCGAATATGCGGCGTCGAGAACGAATCTGGTTCGCAAGACGGACCGCGGATTCG
CGATTTCCGAGCATTACACCGCGCAAGCGCGCTTTCTGTTGAACCTGTACGCCGGCGCTTATGCCGATAACGCTTCA
CGGCTGGCGACGCTGCTCCGGCGTCCGGCATTGGCGGGCTCCATGATCGACCTCGTCGCGCACGCGCGCGCCTTCGA
CGCGAGCGAGGCCGGCGGTGGAGCGCTGGCGTCGATCGTCACGCAATTGCGTCTCAATCATGTGCTGGATCTGGGAT
GCGGCGCCGGCGCGCTGCTGCGCGAGTTGGCGGCGGACGATCGCGGATTCGTCGGCTGGGGACTGGATCGCAATCCC
GCGATGTGCAAGGCGGCGCGGGTTCGAGCGCGGCAAGCGGGGCTCGCCGCGCGCGTGAAGGTGTTCCACGGCGACGG
CTGGAATCCCGGGGCGTCGGTCCCGCCGCGCGTGCTGGCGCGCGTGCGCAACGTCGTCGCGAGCCAGTTCGTCAACG
AGATGTTTCGCGGCGGCACATCGCGAGTGGAAACGTGGCTTCGGCGCATGCGGCGCTTGCTGCCCGGCCGCATGCTC
GTGATATCGGACTATTACGGCCGCCTCGGCCACGGCACTCACACGATGCGCCGCGAGACGCTGCTGCATGACTATGT
GCAGCTCATATCGGGGCAGGGCGTGCCGCCGCCCGATTCCGACGCGTGGTTGGCCATGTATCGAGCAGCCGGTTGCC
GGCCTCTGCACGTGATCGAAGACCGGGCGGCCACGACATGCTTCGTCCATCTGGTGGGGCTATAG

Figure S1: Deconvoluted HR-ESI mass spectra of BthA coexpressed with modifying enzymes in *E. coli*, cleaved with GluC or LysC. The sequence of the peptide is shown below with the cut site bolded. *: sodium adduct, **: potassium adduct, ***: serine phosphorylation in the GSS sequence on the N-terminus. ****: gluconylation on the N-terminus.

GSSHHHHHHMATKPKKTKGVSVSIEGKLPKMTLDMPVDAKKIKAIQKCLENGKLTITMS**K**VDLA
GGRMG**E**GYLYD

a) BthA (no modifying enzymes). Calculated exact mass for BthA: 8421.3619 Da. Calculated exact mass for GluC-cleaved BthA: 629.2696 Da

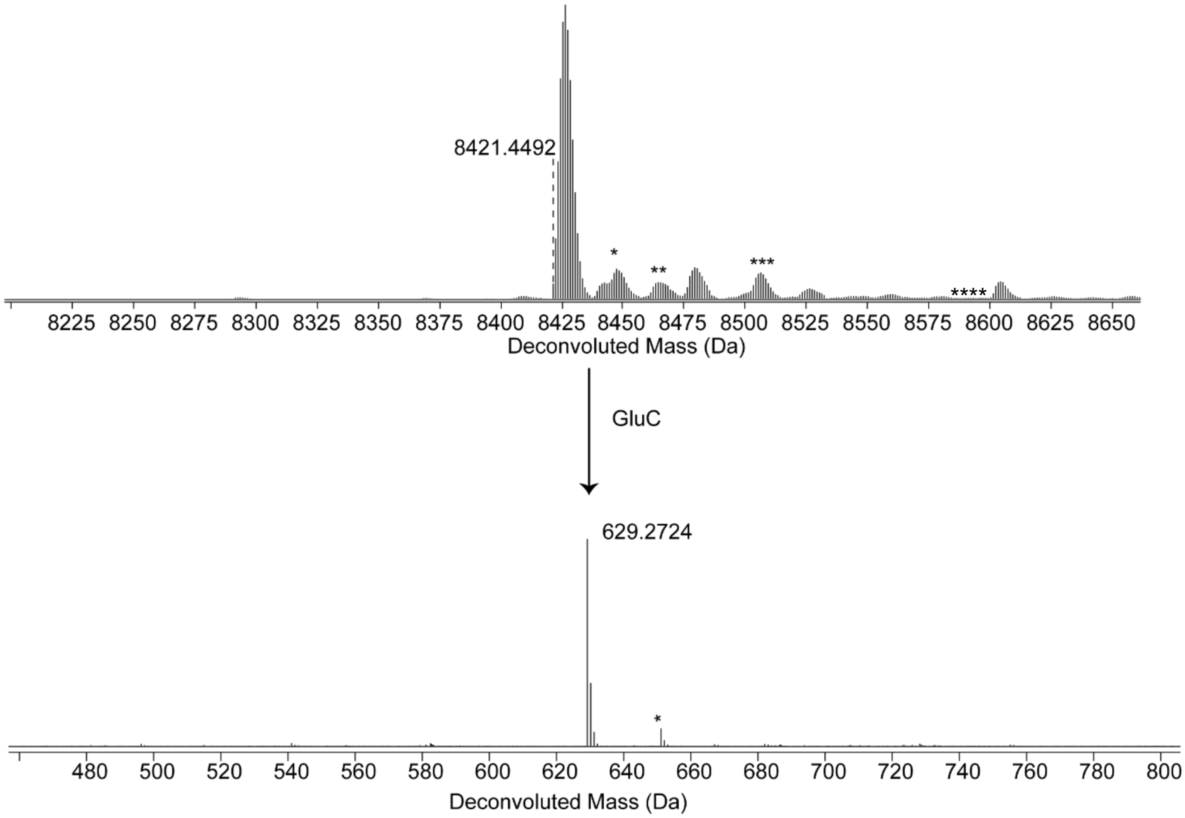

b) BthA coexpressed with BthD. Calculated exact mass for BthA: 8421.3619 Da. Calculated exact mass for GluC-cleaved BthA: 629.2696 Da. Calculated exact mass for BthD-modified BthA: 8435.3775 Da. Calculated exact mass for GluC-cleaved BthD-modified BthA: 643.2852 Da.

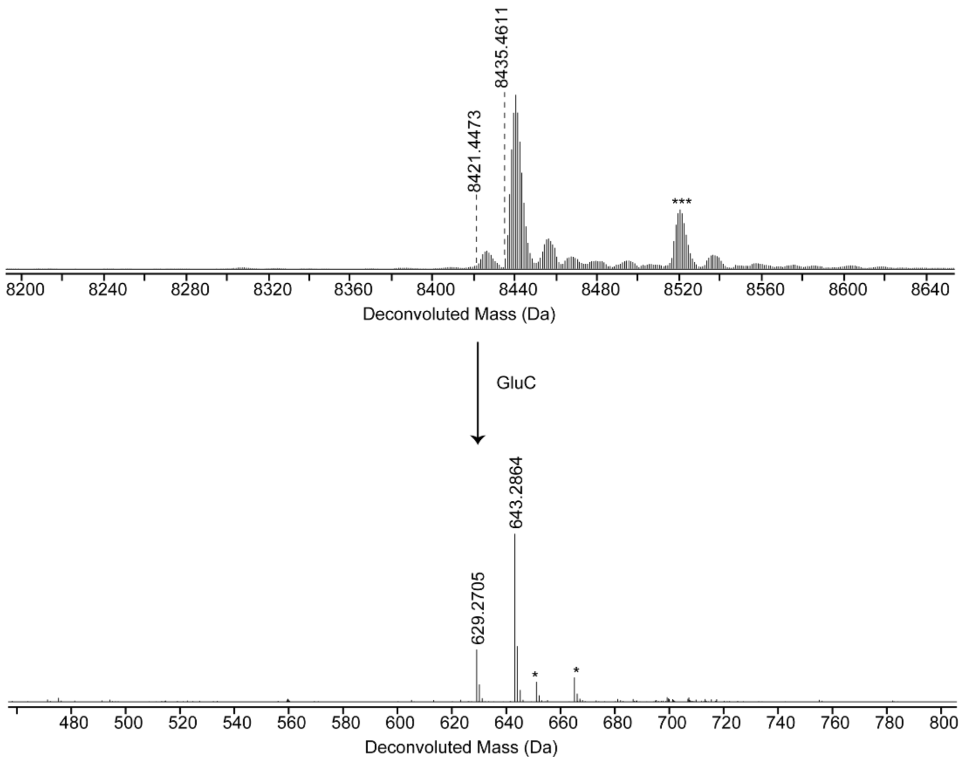

c) BthA coexpressed with BthO. Calculated exact mass for BthA: 8421.3619 Da. Calculated exact mass for GluC-cleaved BthA: 629.2696 Da. Calculated exact mass for BthO-modified BthA: 8419.3463 Da. Calculated exact mass for GluC-cleaved BthO-modified BthA: 627.2540 Da.

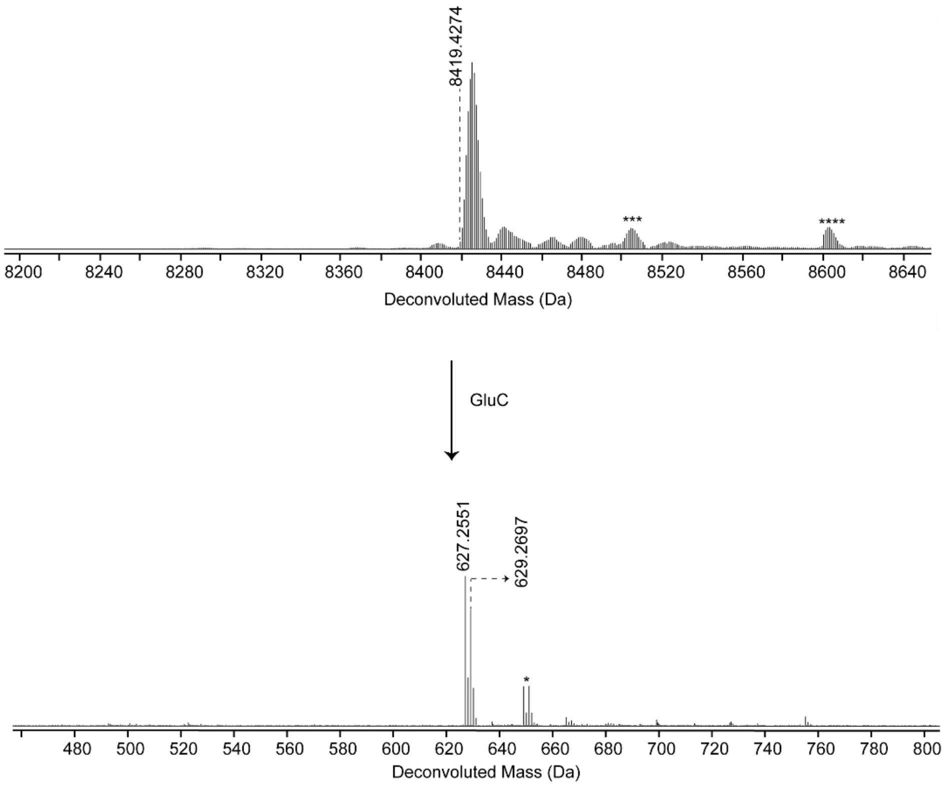

d) BthA coexpressed with BthD and BthO. Calculated exact mass for GluC-cleaved BthA: 629.2696 Da. Calculated exact mass for GluC-cleaved BthO-modified BthA: 627.2540 Da. Calculated exact mass for GluC-cleaved BthD-modified BthA: 643.2852 Da. Calculated exact mass for GluC-cleaved BthDO-modified BthA: 641.2696 Da.

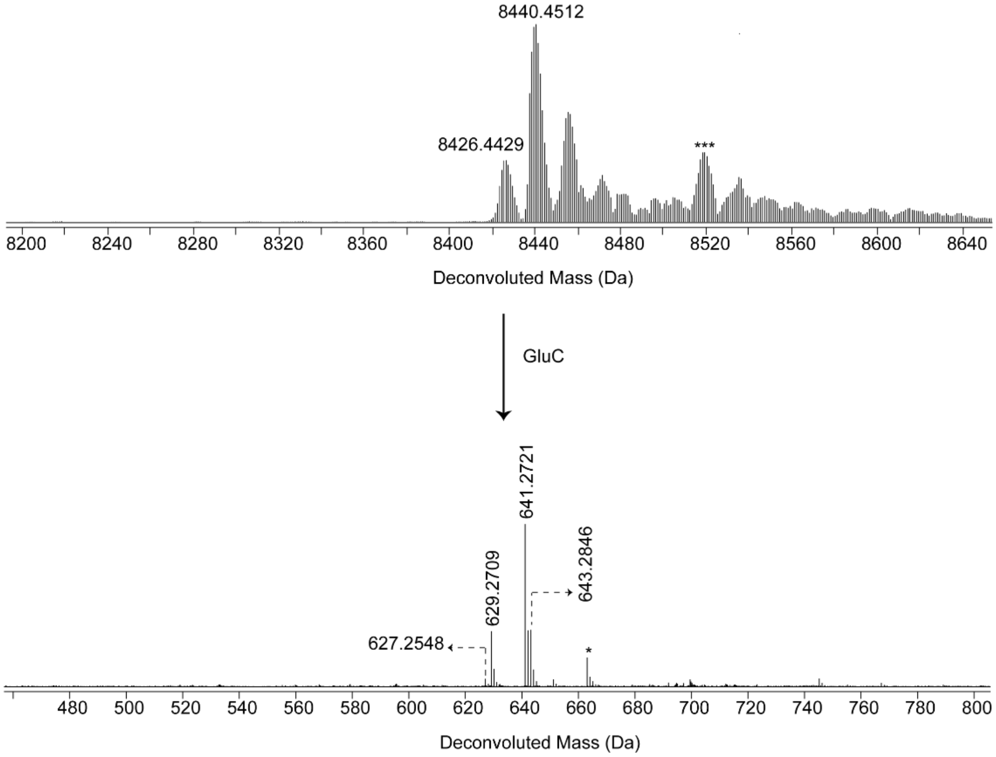

d) BthA coexpressed with BthD, BthO, BthH, BthI, and BthS. BthHI and BthS were not functional. Calculated exact mass for LysC-cleaved BthA: 1614.7347 Da. Calculated exact mass for LysC-cleaved BthO-modified BthA: 1612.7191 Da. Calculated exact mass for LysC-cleaved BthD-modified BthA: 1628.7503 Da. Calculated exact mass for LysC-cleaved BthDO-modified BthA: 1626.7347 Da

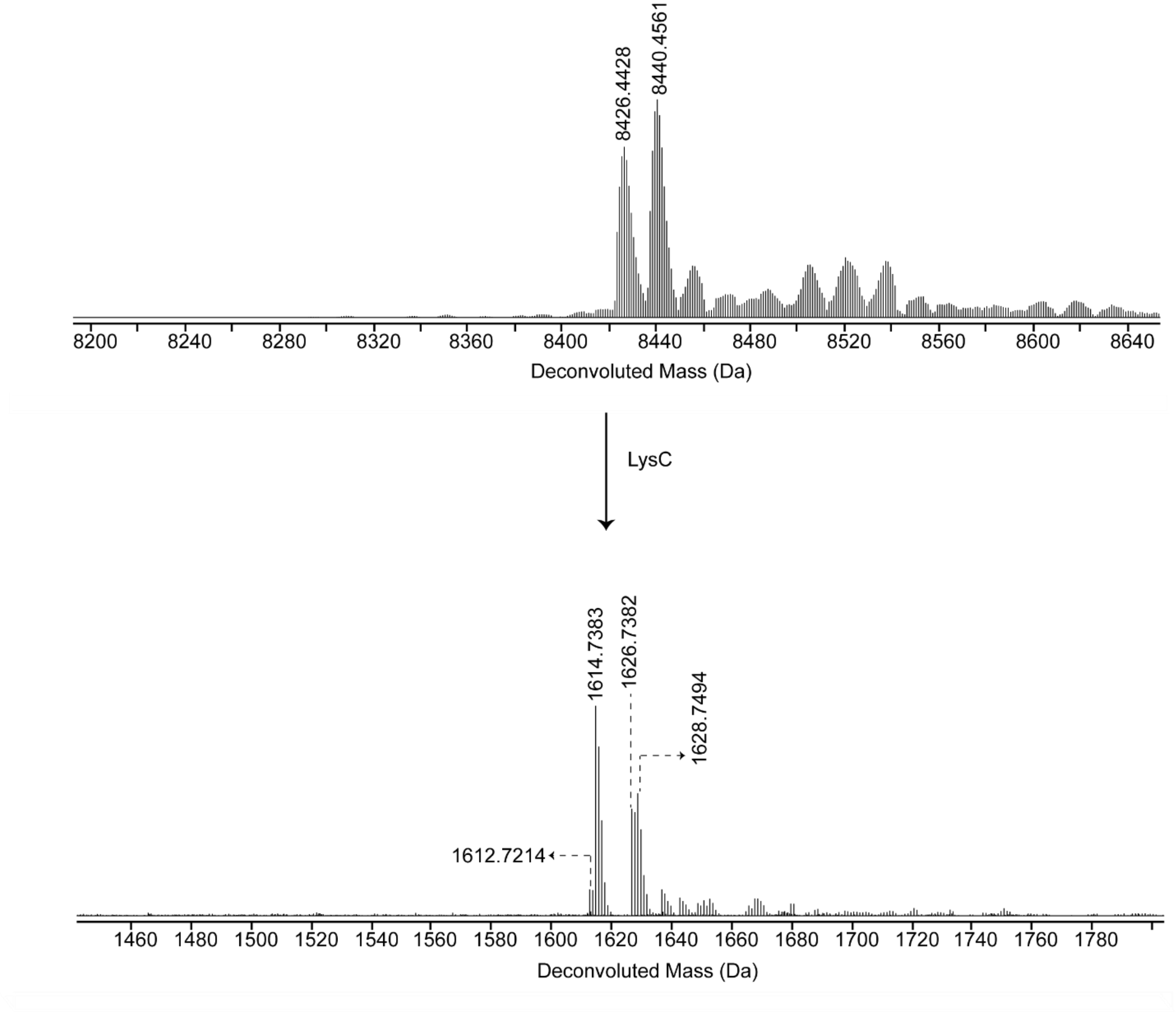

e) BthA coexpressed with BthH and BthI. BthHI were not functional. Calculated exact mass for LysC-cleaved BthA: 1614.7347 Da.

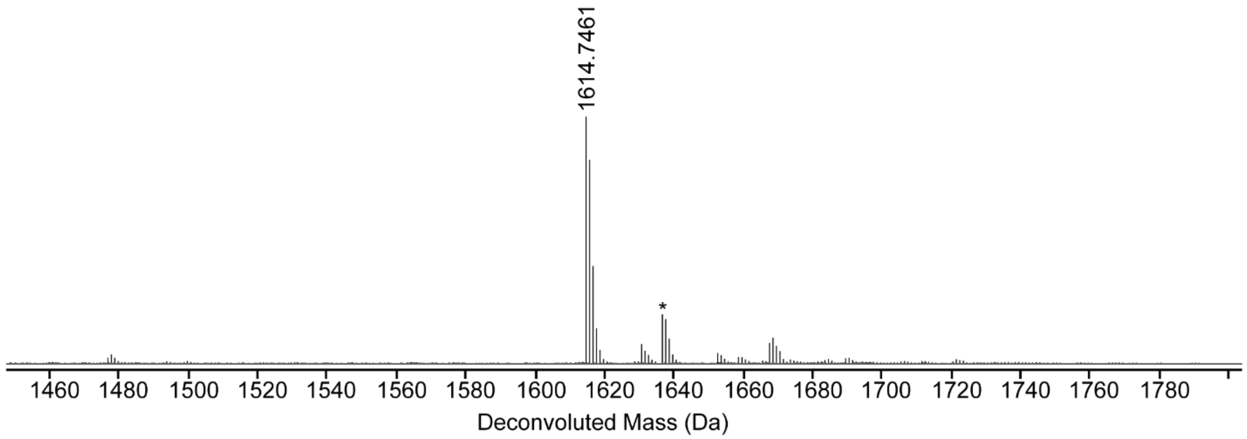

Figure S2: HR-ESI-MS/MS spectra of BthA coexpressed with modifying enzymes in *E. coli* followed by GluC cleavage. Int: internal fragment.

a) BthA coexpressed with BthD.

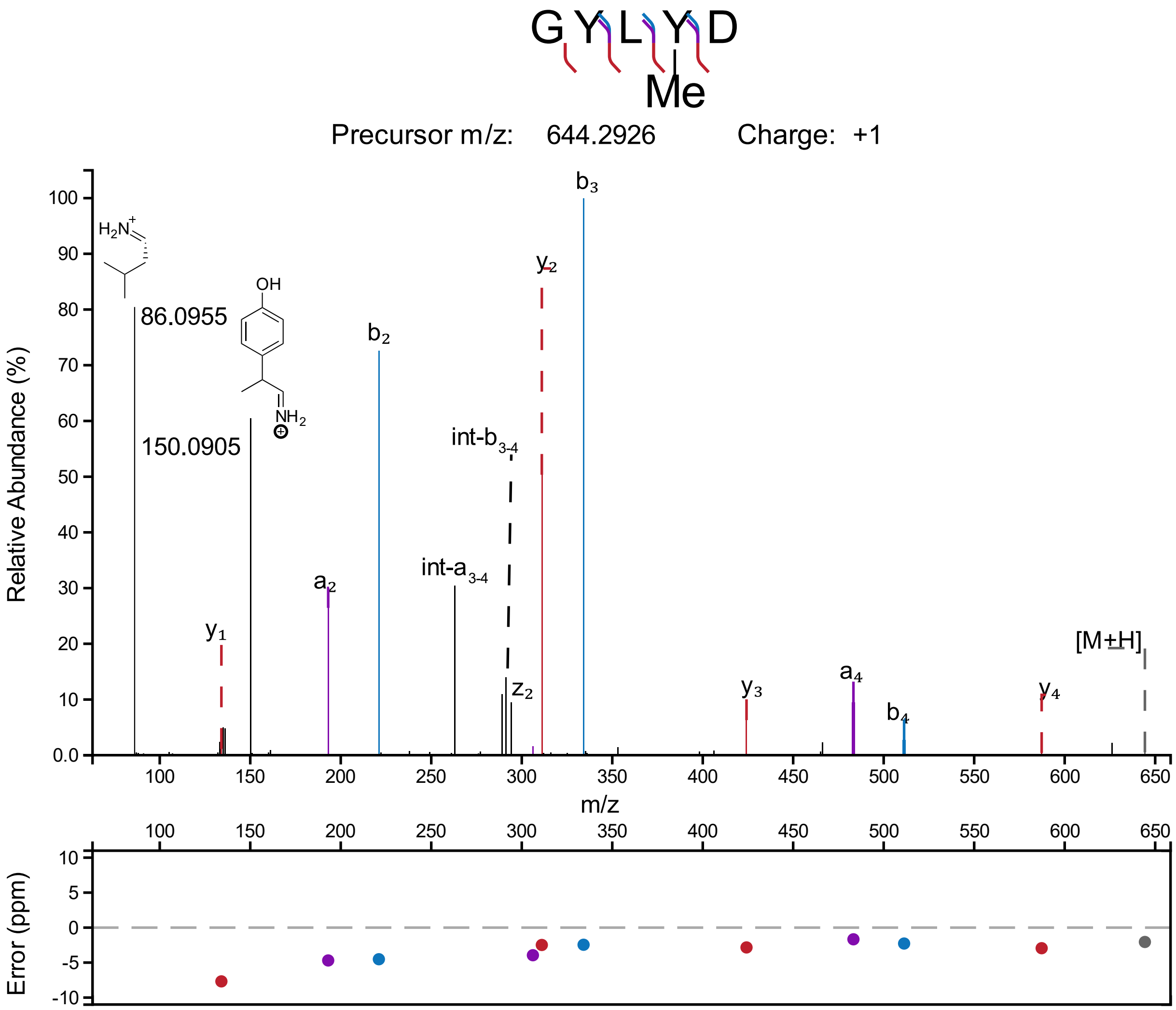

b) BthA coexpressed with BthO.

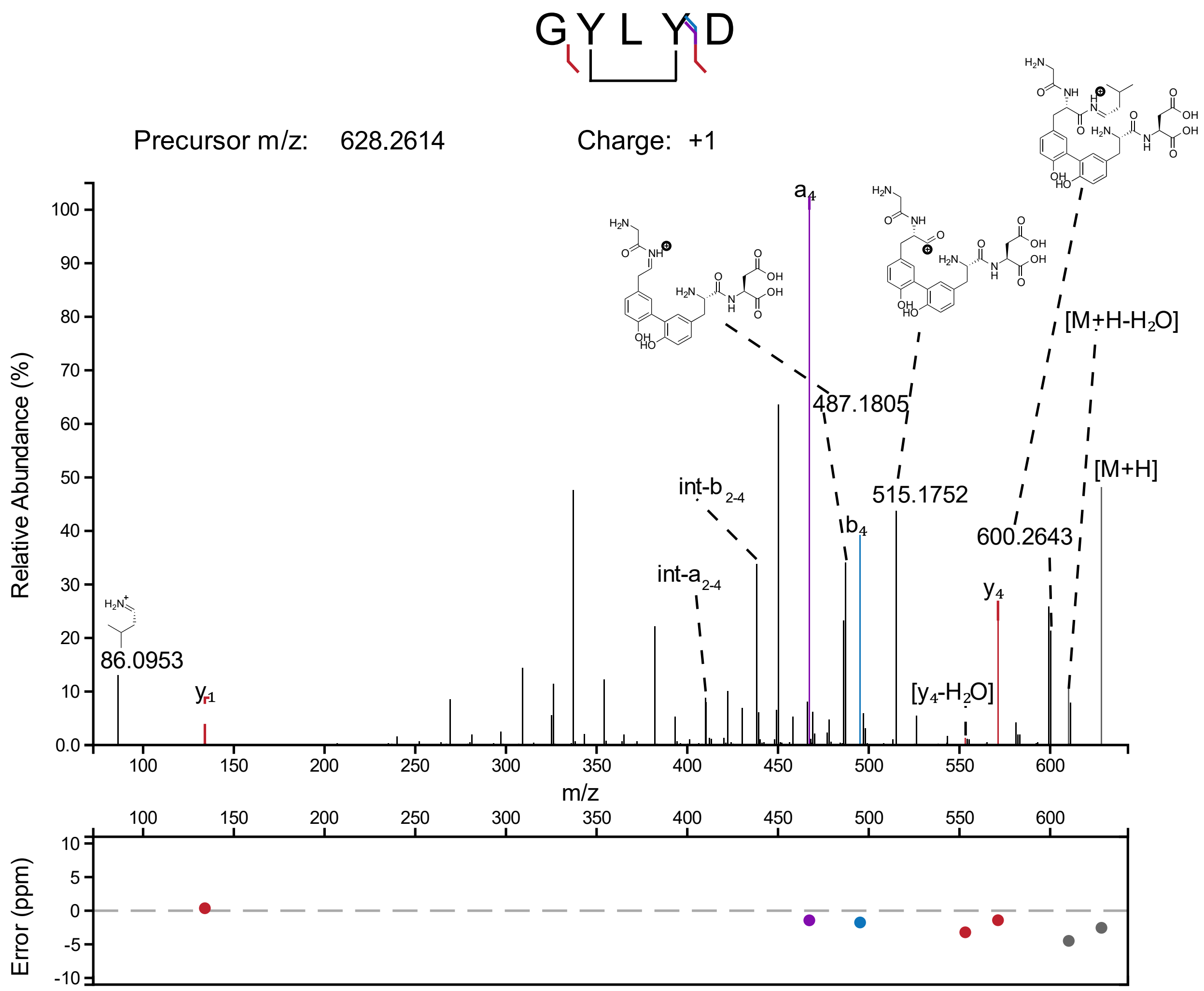

c) BthA coexpressed with BthD and BthO

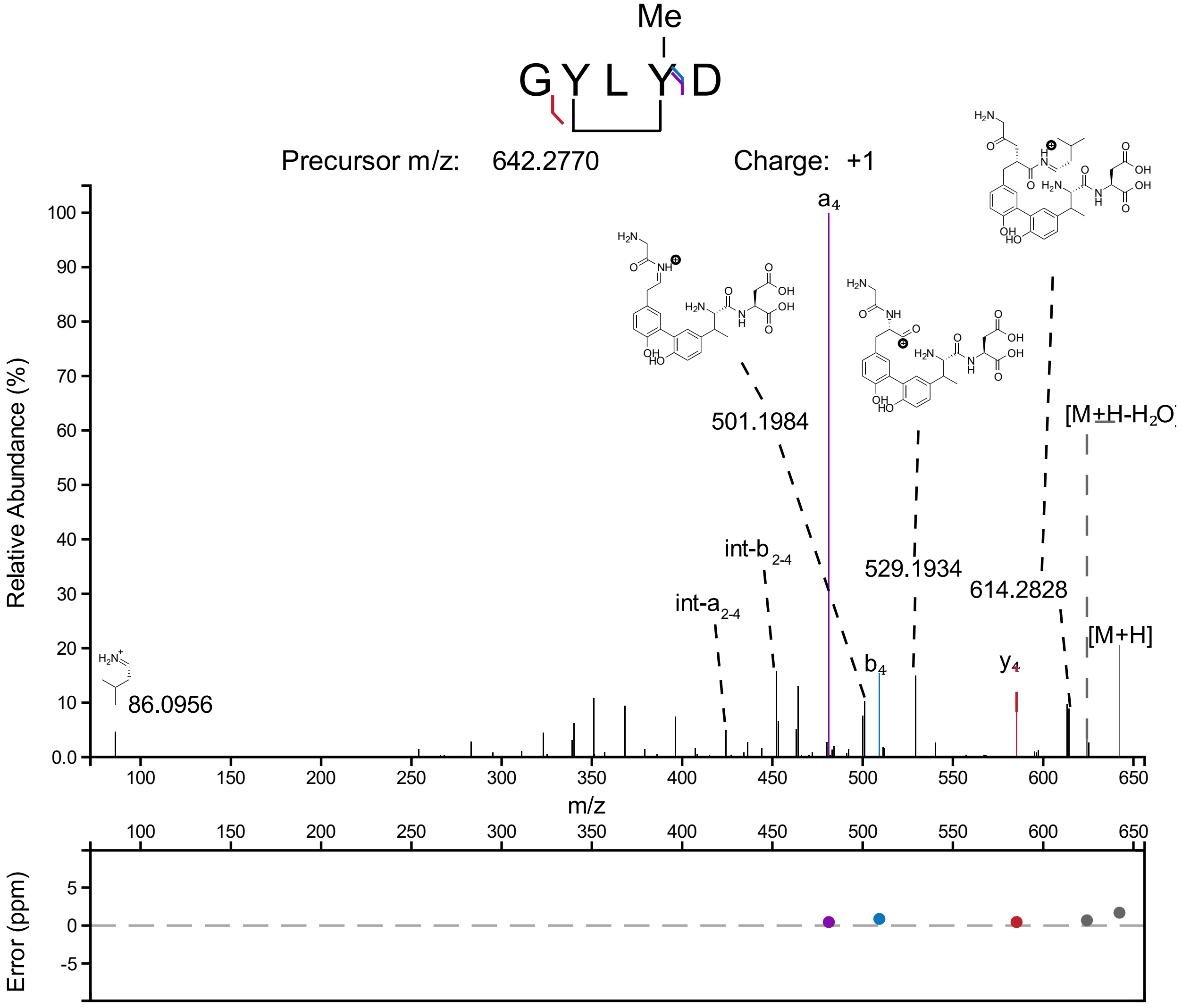

Figure S3: Deconvoluted HR-ESI mass spectra of BthA coexpressed with all modifying enzymes in *Burkholderia* sp. FERM BP-3421 using the initial pSCrhaB2 constructs (Table S2b). The two constructs (with or without the first 833 bp) yielded similar results (panels a and b). The sequence of the peptide is shown below with the cut site bolded (endoproteinase GluC was used). *: sodium adduct, #: potential methionine oxidation; absent in fragment after proteolysis. Calculated exact mass for BthO/H/I/S-modified BthA: 8088.2156 Da. Calculated exact mass for GluC-cleaved BthO/H/I/S-modified BthA: 611.2591 Da

MHHHHHHMATKPKKTKGVSVSIEGKLPKMTLDMPVDAKKIKAIQKCLENGKLTITMSKVDLAGG
RMG**E**GYLYD

a) Product resulting from construct in Table S2b

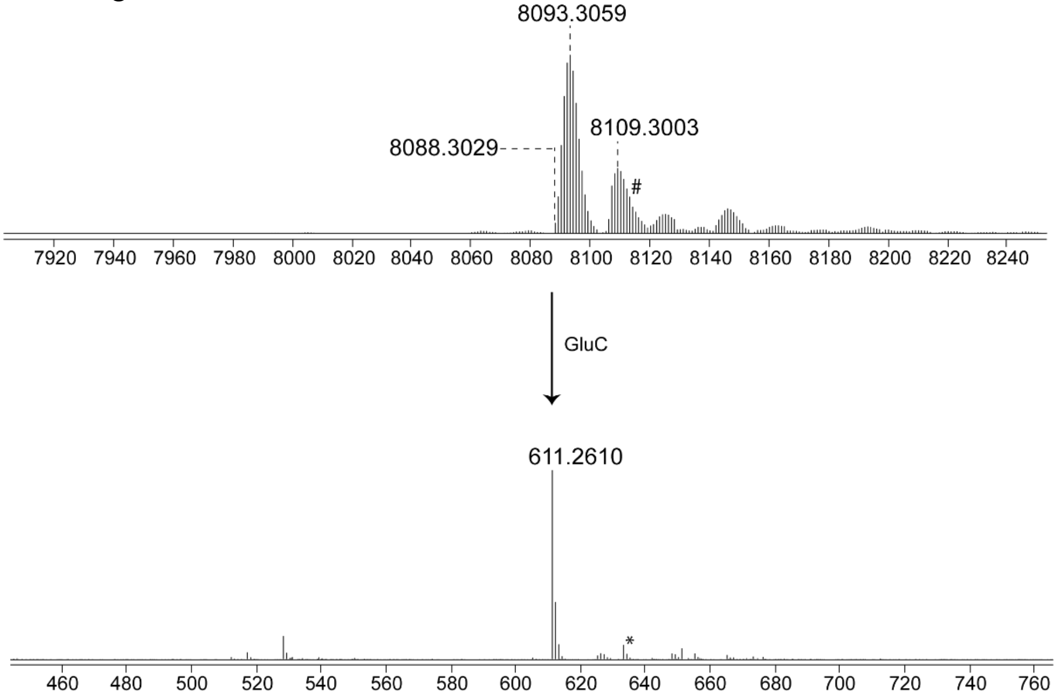

b) Construct in Table S2b but with first 833 bp of the insert removed.

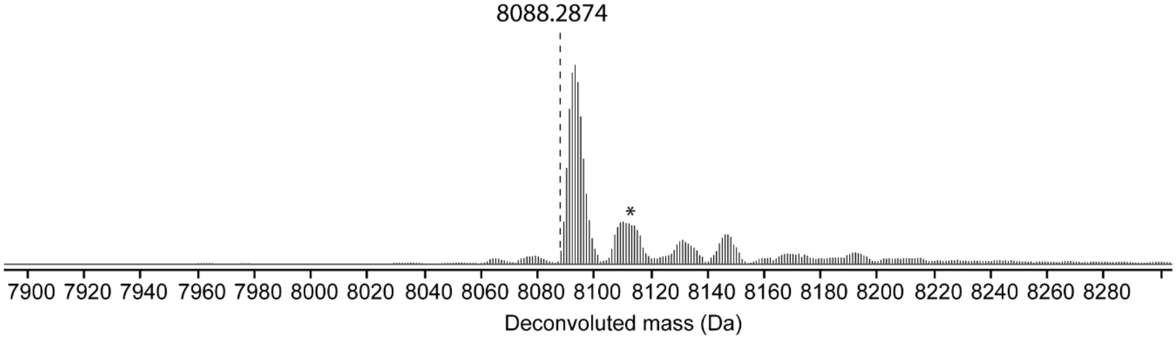

Figure S4: HR-ESI-MS/MS of BthA coexpressed with all modifying enzymes in *Burkholderia* sp. FERM BP-3421 (using the initial construct; Table S2b) followed by GluC cleavage. BthS, BthHI and BthO were active. D* here shows the modified aspartate (3-amino-2-oxobutanoic acid).

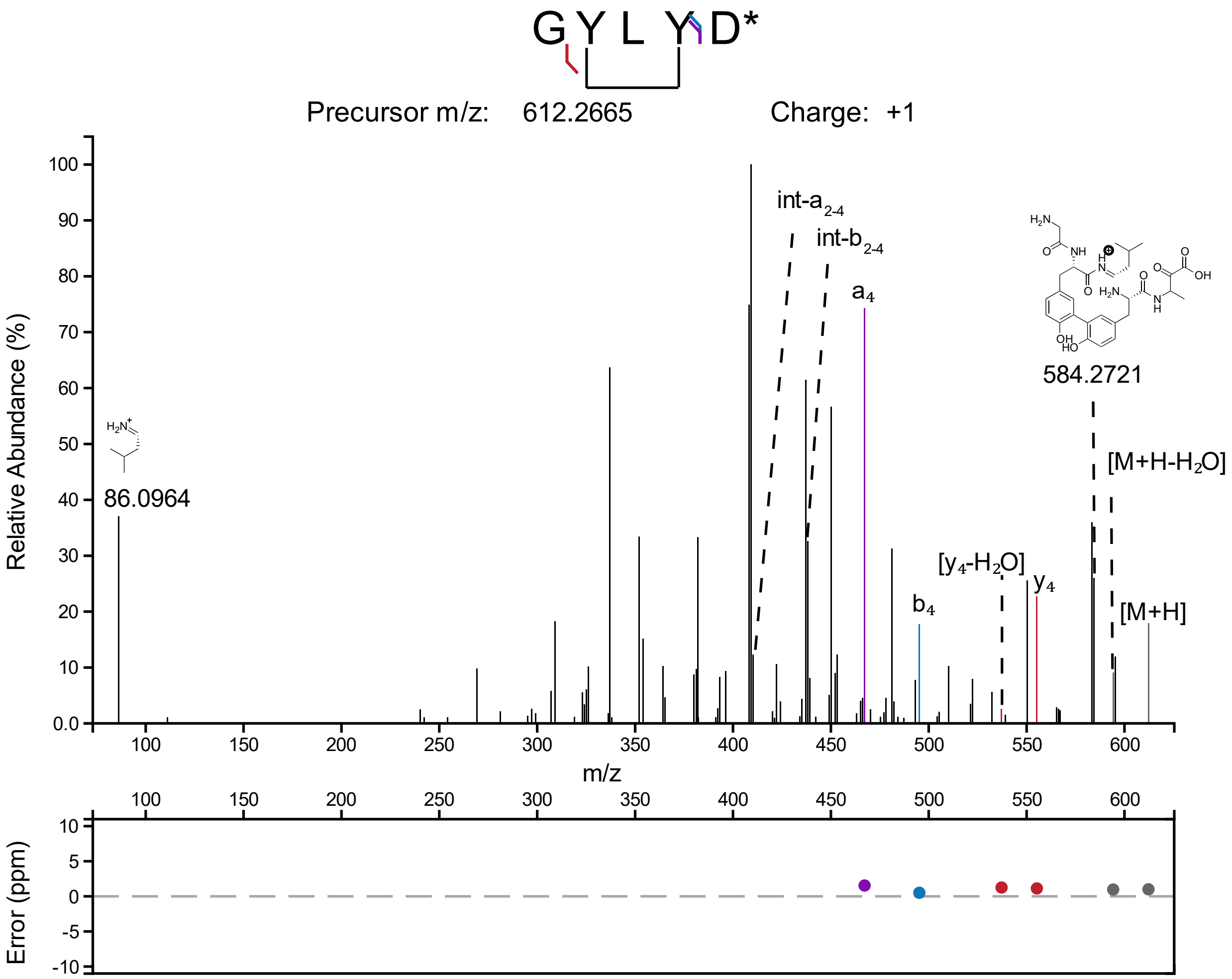

.

Figure S5: Deconvoluted HR-ESI mass spectrum of BthA coexpressed with the modifying enzymes in the initial pSCrhaB2 construct (Table S2) with BthH removed. The sequence of the peptide is shown below with the cut site bolded (endoproteinase GluC was used). *: sodium adduct. **: potassium adduct. Calculated exact mass for GluC-cleaved BthO-modified BthA: 627.2540 Da

MHHHHHHMATKPKKTKGVSVSIEGKLPKMTLDMPVDAKKIKAIQKCLENGKLTITMSKVDLAGG
RMG**E**GYLYD

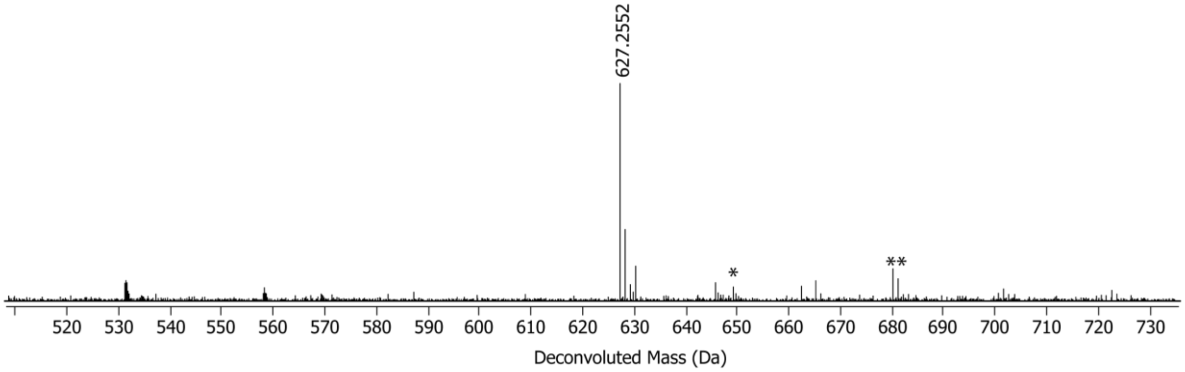

Figure S6: Deconvoluted HR-ESI mass spectrum of BthA coexpressed with the modifying enzymes in the initial pSCrhaB2 construct (Table S2) with BthI removed. The sequence of the peptide is shown below with the cut site bolded (endoproteinase GluC was used). *: sodium adduct. **: potassium adduct. Calculated exact mass for GluC-cleaved BthO-modified BthA: 627.2540 Da

MHHHHHHMATKPKKTKGVSVSIEGKLPKMTLDMPVDAKKIKAIQKCLENGKLTITMSKVDLAGG
RMG**E**GYLYD

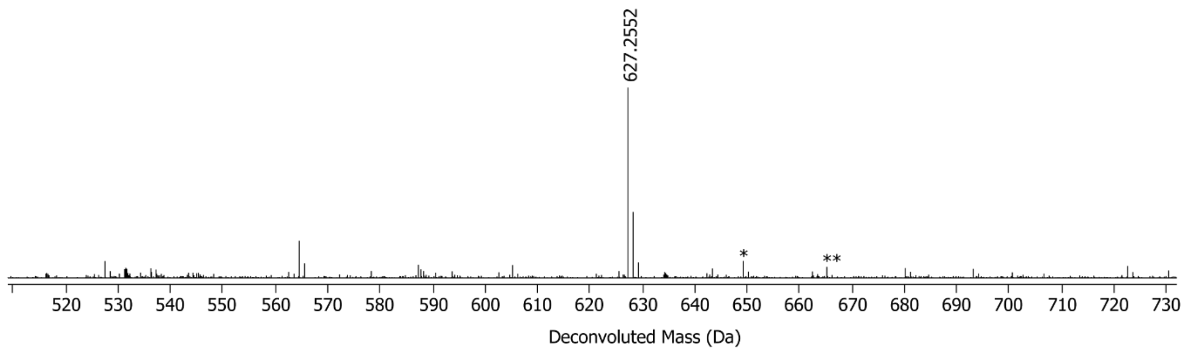

Figure S7: The HR-ESI-MS/MS spectrum of BthA coexpressed with the modifying enzymes in the initial pSCrhaB2 construct (Table S2) with BthH removed.

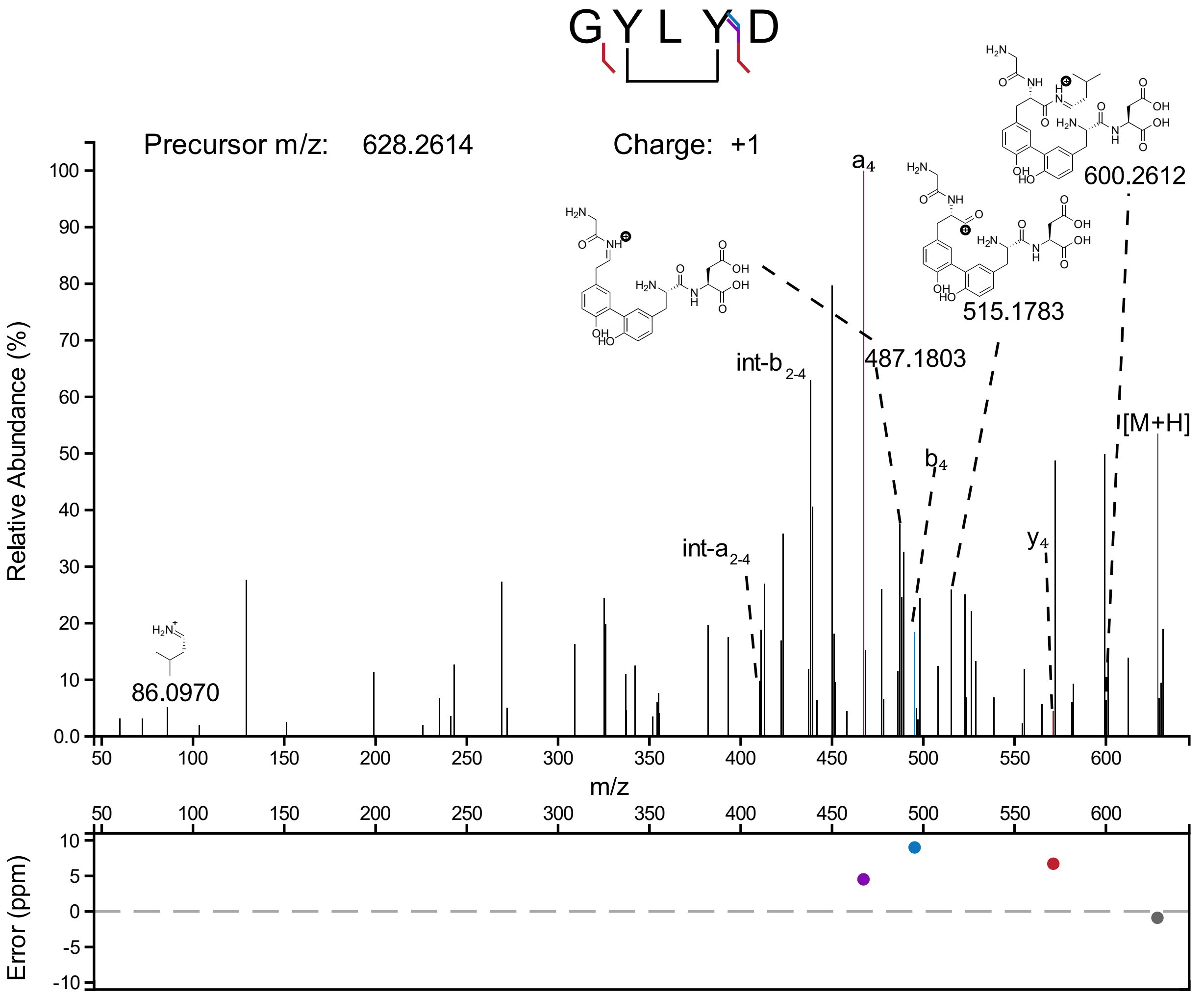

Figure S8: The HR-ESI-MS/MS spectrum of BthA coexpressed with the modifying enzymes in the initial pSCrhaB2 construct (Table S2) with BthI removed.

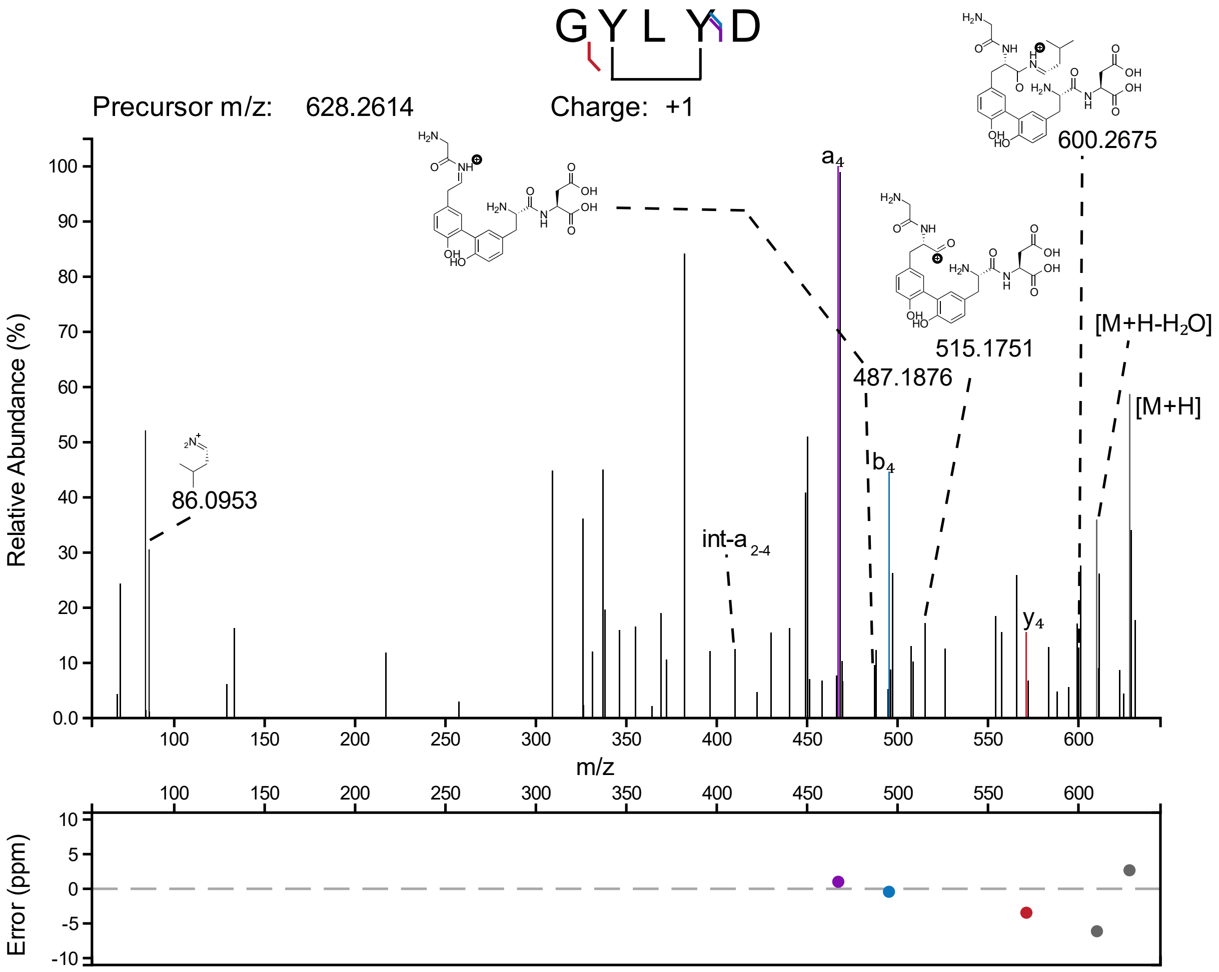

Figure S9: Deconvoluted HR-ESI mass spectra of BthA coexpressed with modifying enzymes (BthS removed) in *Burkholderia* sp. FERM BP-3421 using the initial pSCrhaB2 constructs (Table S2). The sequence of the peptide is shown below with the cut site bolded (endoproteinase GluC was used). *: sodium adduct, **: potassium adduct, #: potential methionine oxidation (consistent with the peak disappearing in the GluC-digested peptide. Calculated exact mass for BthO/H/I-modified BthA: 8074.2000 Da. Calculated exact mass for GluC-cleaved BthO/H/I-modified BthA: 597.2435 Da

MHHHHHHMATKPKKTKGVSVSIEGKLPKMTLDMPVDAKKIKAIQKCLENGKLTITMSKVDLAGG
RMG**E**GYLYD

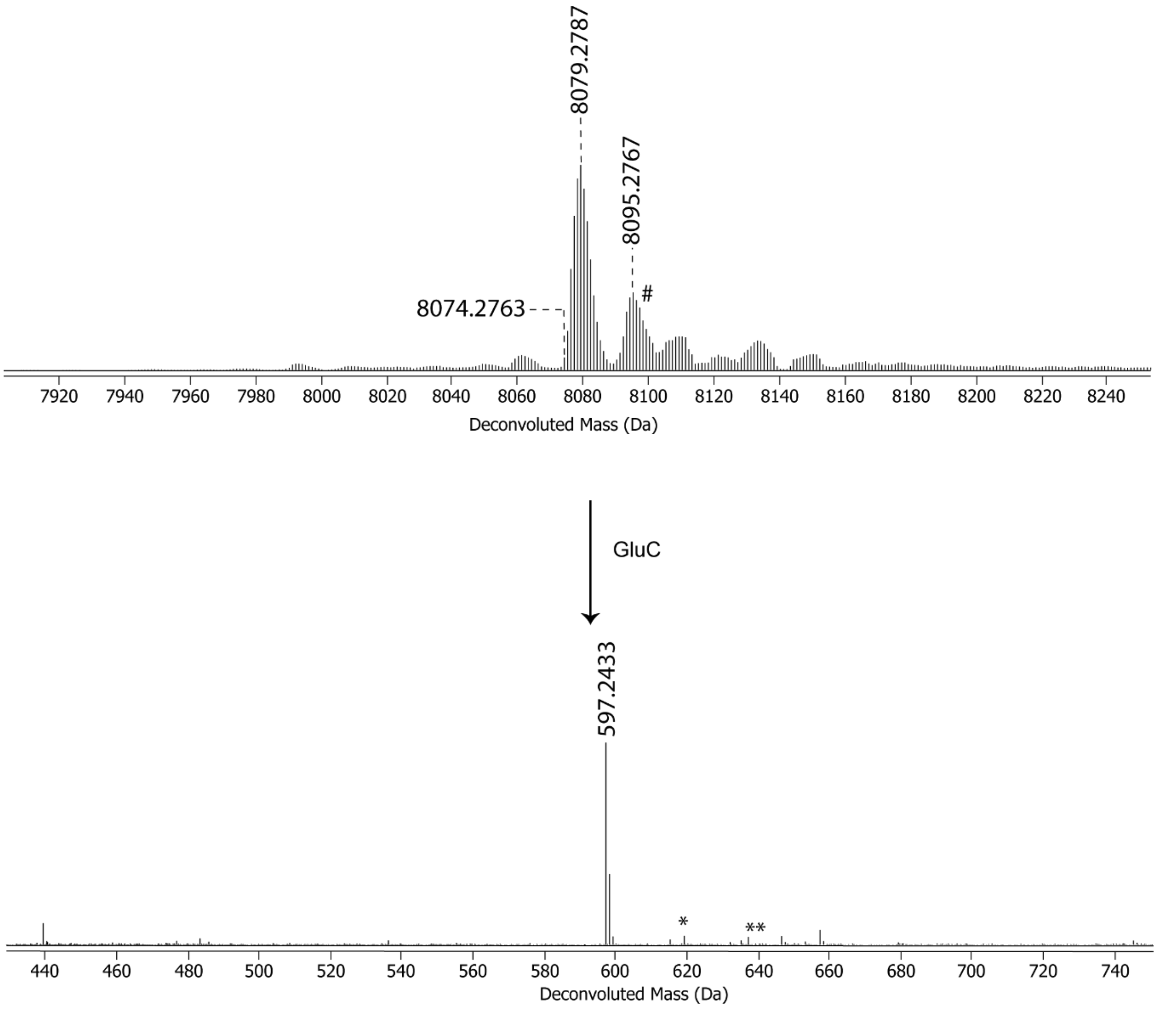

Figure S10: HR-ESI-MS/MS spectrum of BthA coexpressed with the modifying enzymes in the initial pSCrhaB2 construct (Table S2) with BthS removed. D** here shows the modified aspartate (aminopyruvic acid).

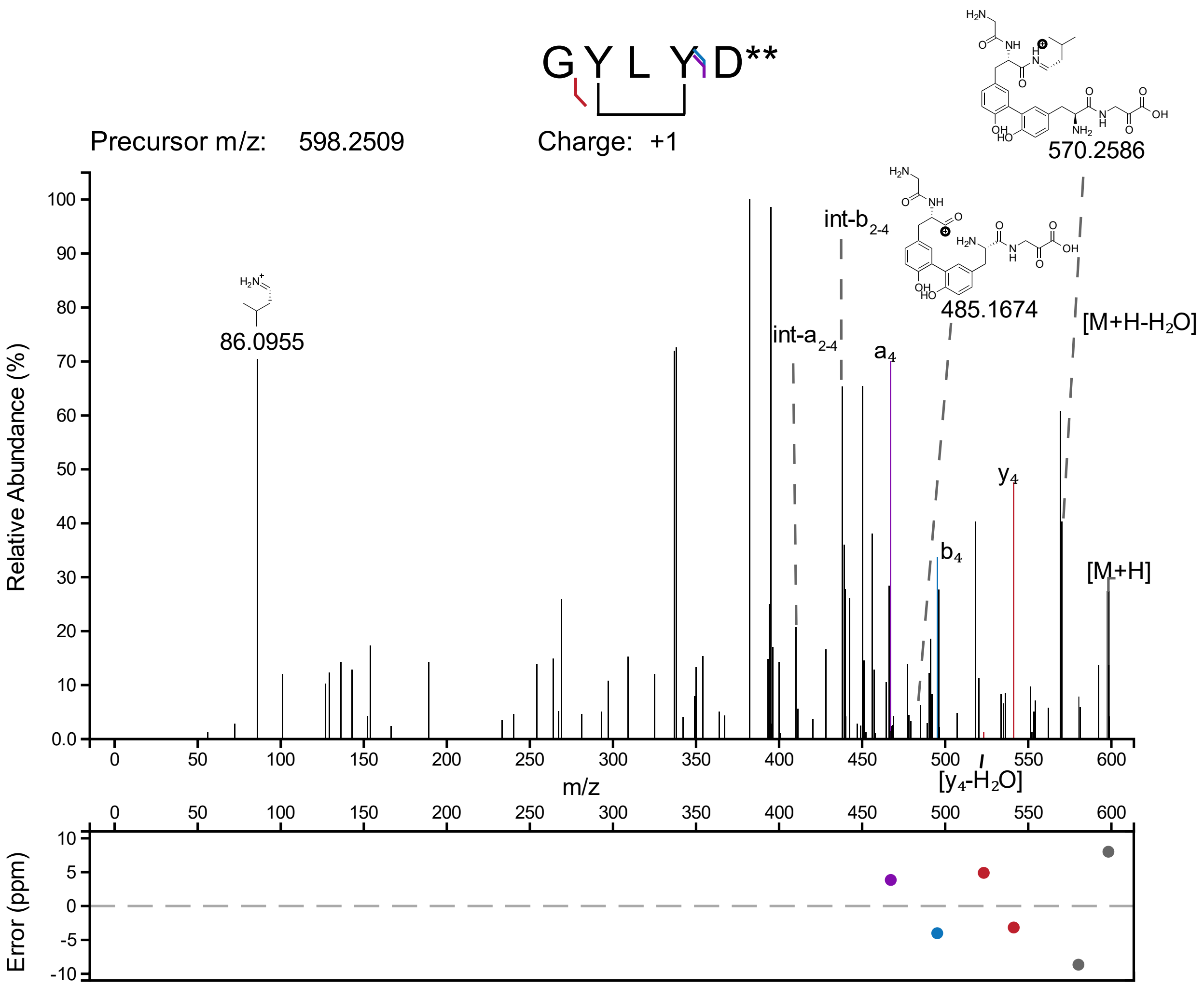

Figure S11: Deconvoluted HR-ESI mass spectrum of BthA coexpressed with modifying enzymes (BthS removed) in *E. coli* using the initial pSCrhaB2 construct (Table S2). The sequence of the peptide is shown below with the cut site bolded (endoproteinase LysC was used). *: sodium adduct, **: potassium adduct. Calculated exact mass for LysC-cleaved BthO-modified BthA: 1612.7191 Da

*MHHHHHHMATKPKKTKGVSVSIEGKLPKMTLDMPVDAKKIKAIQKCLENGKLTITMS**K**VDLAGG*
RMGEGYLYD

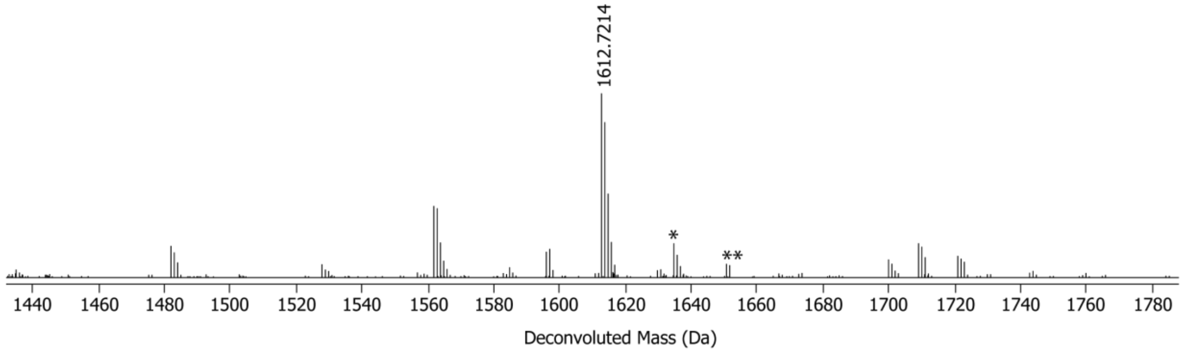

Figure S12: Deconvoluted HR-ESI mass spectra of BthA coexpressed with modifying enzymes (BthS removed) in *Burkholderia* sp. FERM BP-3421 using the redesigned pSCrhaB2 construct (Table S3). The sequence of the peptide is shown below with the cut site bolded (endoproteinase GluC was used). #: potential methionine oxidation (consistent with the oxidation peak not being present in the GluC-digested peptide). Calculated exact mass for BthO/H/I-modified BthA: 8102.2312 Da. Calculated exact mass for GluC-cleaved BthO/H/I-modified BthA: 625.2747 Da.

MHHHHHHMATKPKKTKGVSVSIEGKLPKMTLDMPVDAKKIKAIQKCLENGKLTITMSKVDLAGG
RMG**E**GYLYD

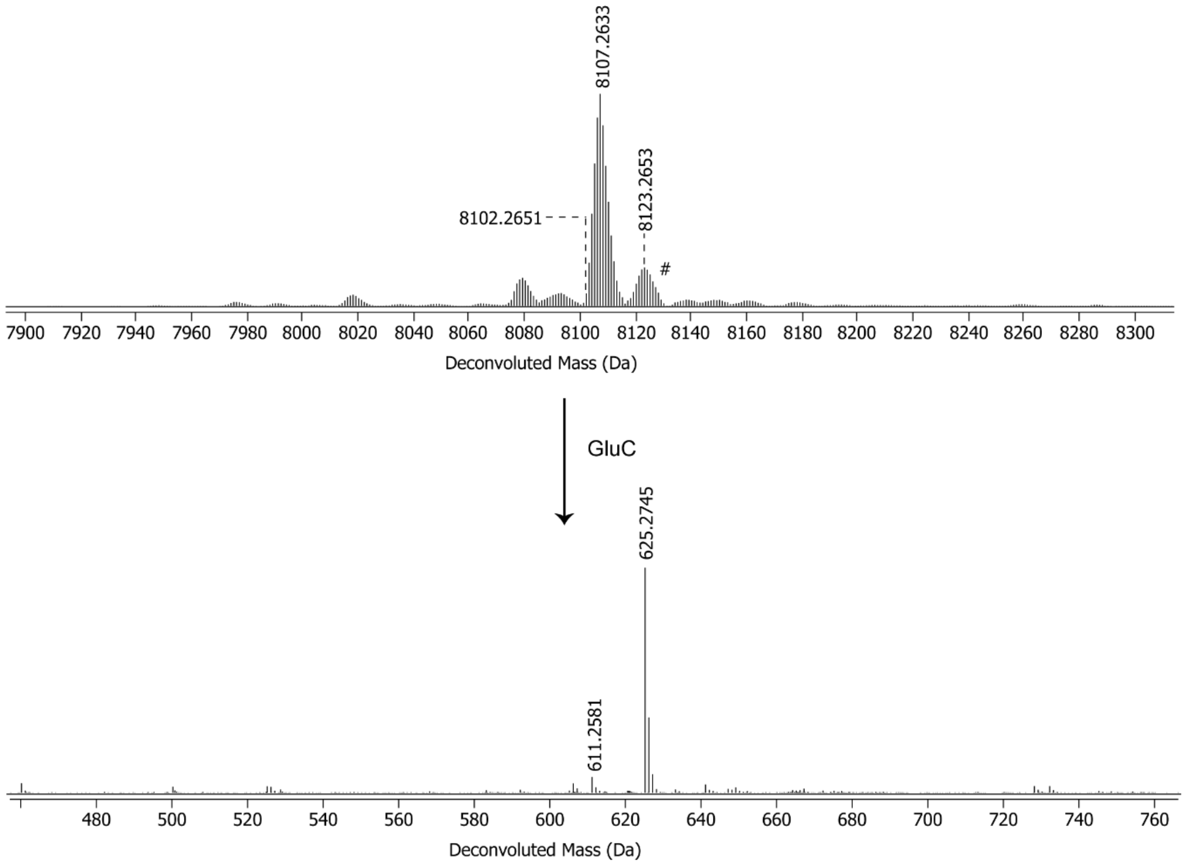

Figure S13: The HR-ESI-MS/MS spectrum of BthA coexpressed with the modifying enzymes in the redesigned pSCrhaB2 construct (Table S3). D* here shows the modified aspartate (3-amino-2-oxobutanoic acid).

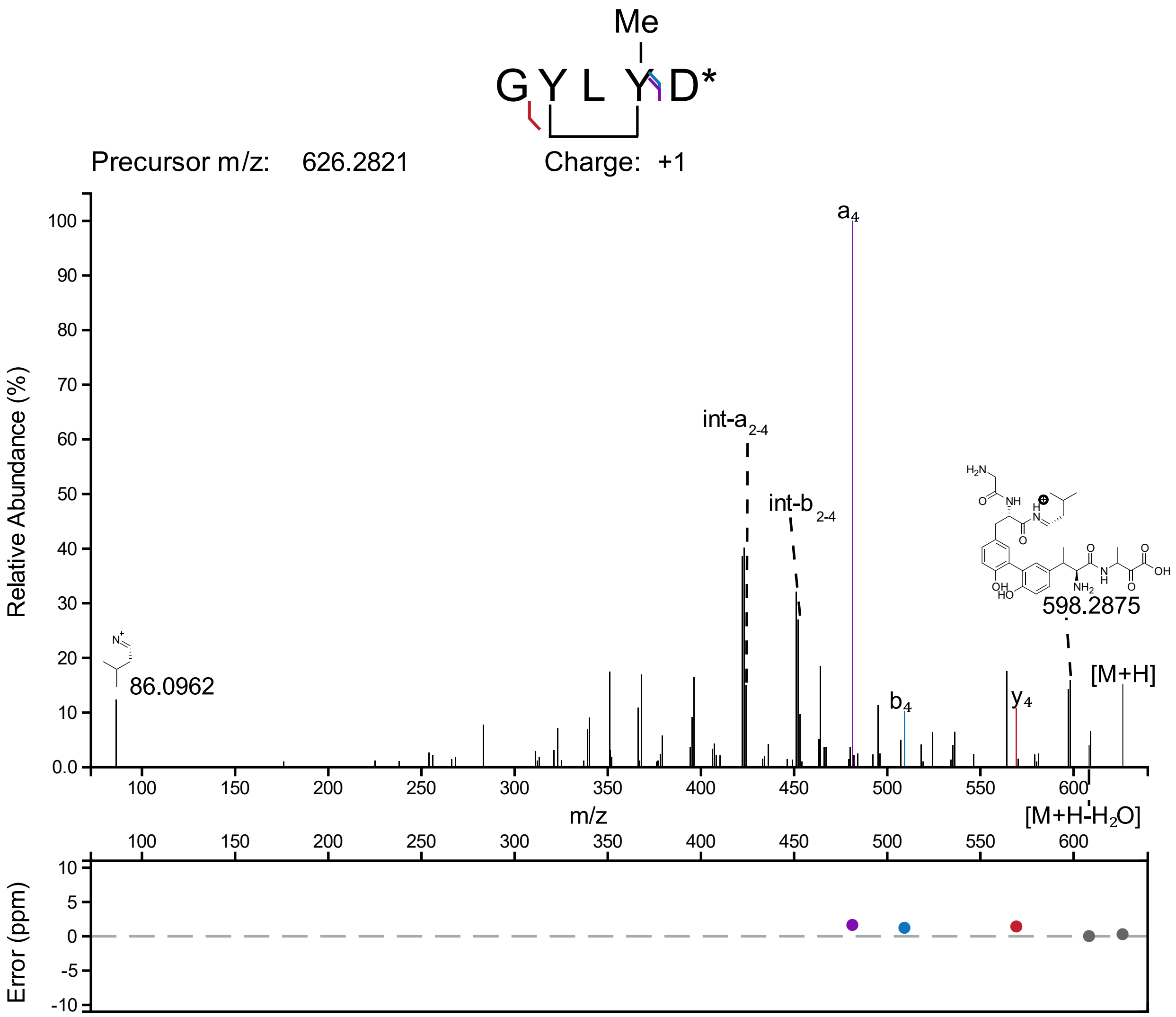

Figure S14: NMR spectra of BthAD-modified product after GluC cleavage. The solvent used was 40% ACN-d3/60% H_2O. The structure of the product is shown in Figure 3 (main text).

a) ^{1}H-NMR

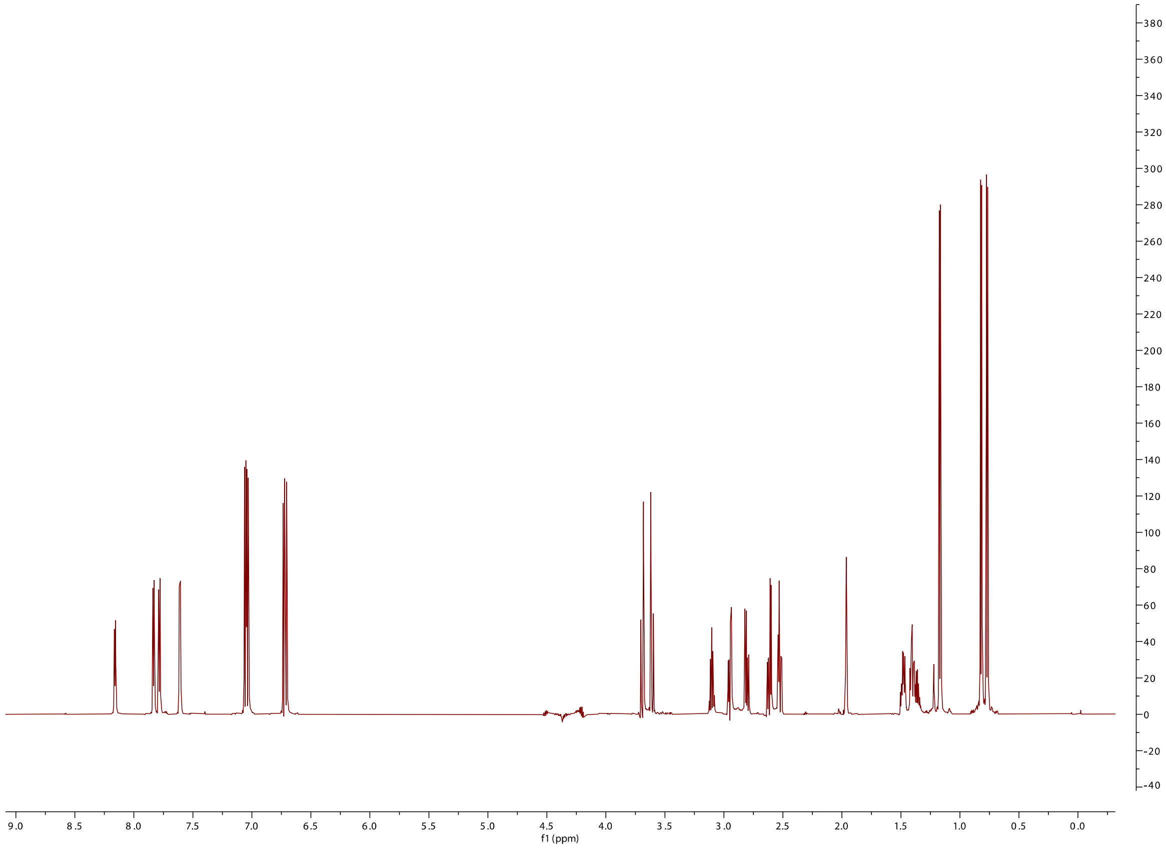

b) ^{1}H-^{1}H TOCSY

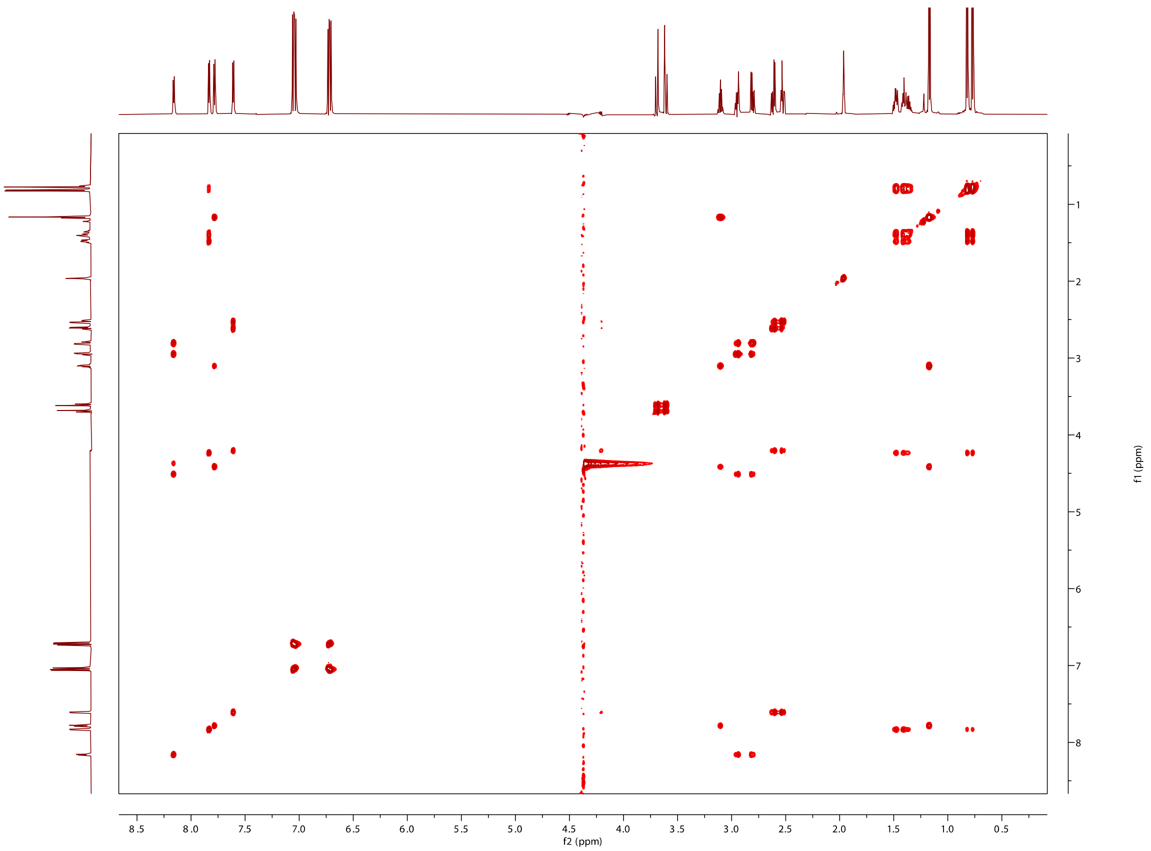

8.5
8.0
7.5
7.0
6.5
6.0
5.5
5.0
4.5
4.0
3.5
3.0
2.5
2.0
1.5
1.0
0.5
f2 (ppm)
f1 (ppm)
1
2
3
4
5
6
7
8

c) ^{1}H-^{1}H ROESY

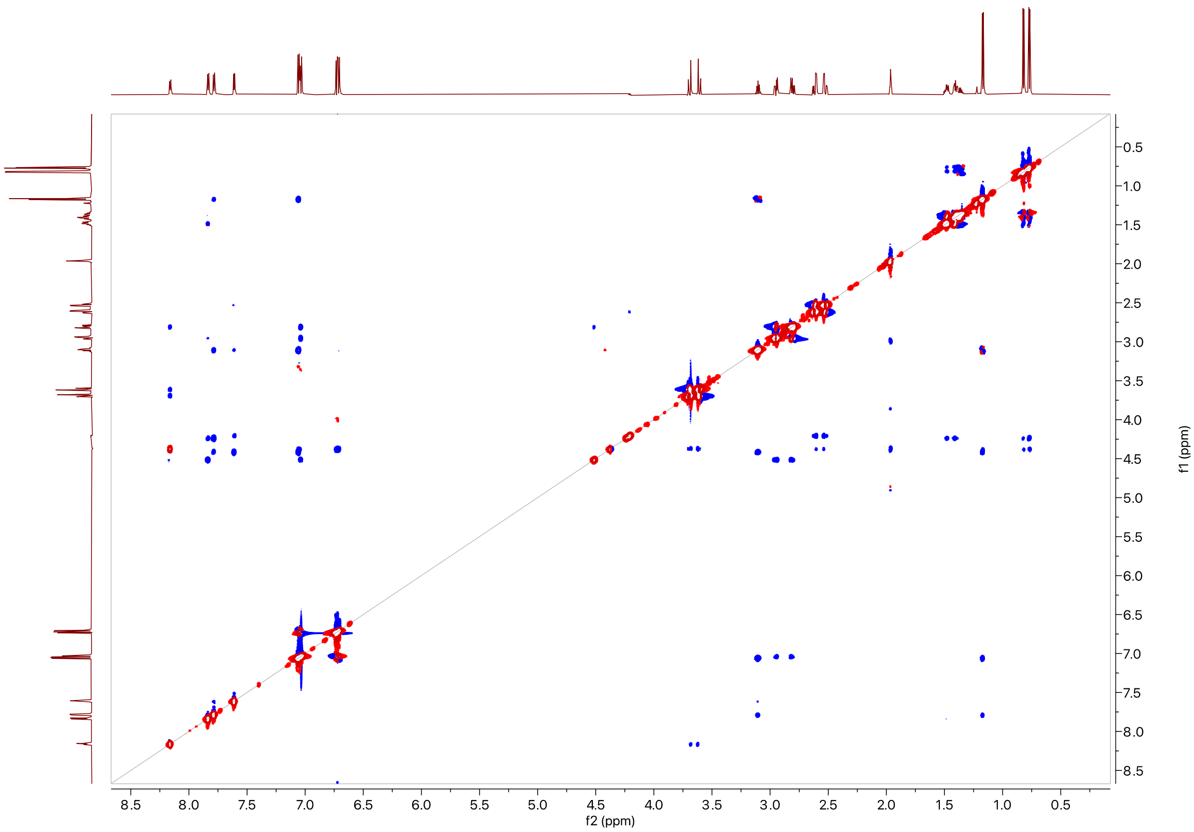

d) ^{1}H-^{13}C HSQC

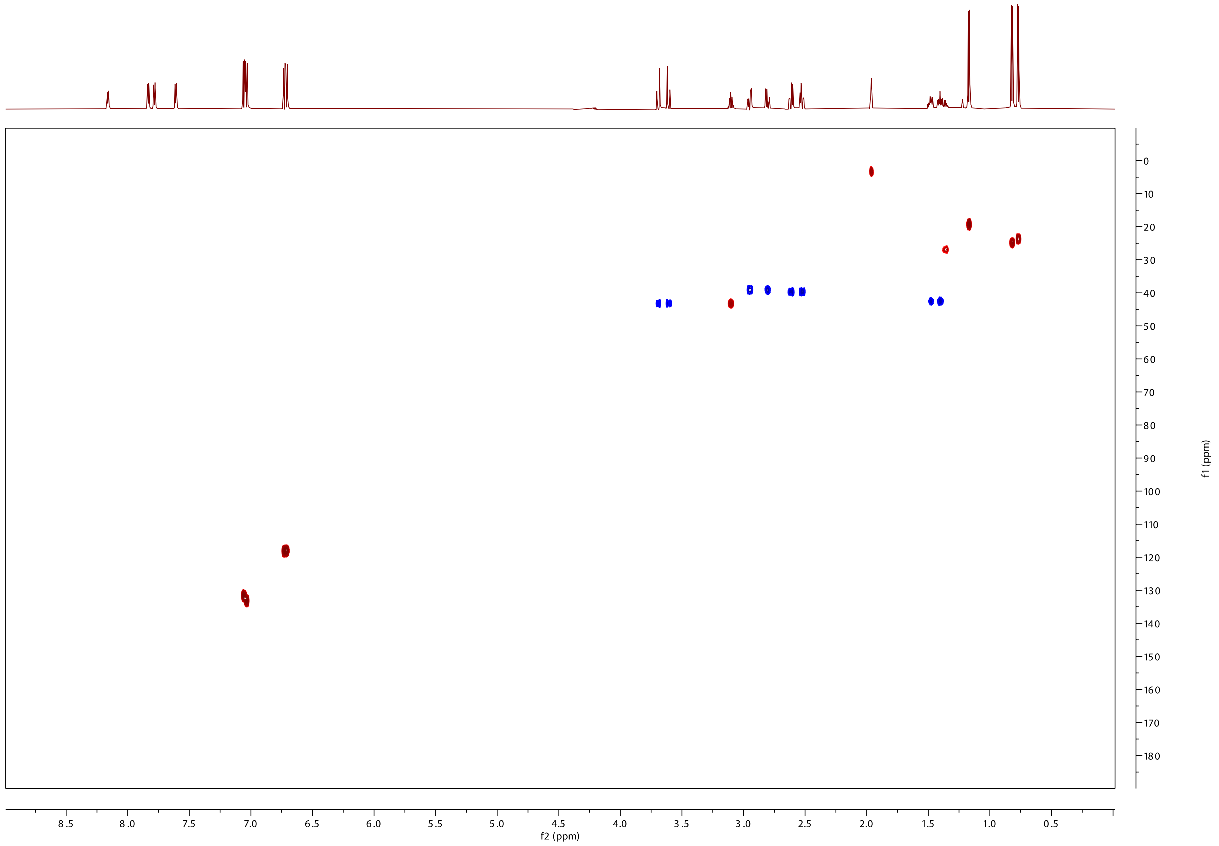
f1 (ppm)
f2 (ppm)
8.5 8.0 7.5 7.0 6.5 6.0 5.5 5.0 4.5 4.0 3.5 3.0 2.5 2.0 1.5 1.0 0.5
0 10 20 30 40 50 60 70 80 90 100 110 120 130 140 150 160 170 180

d) ^{1}H-^{13}C HMBC

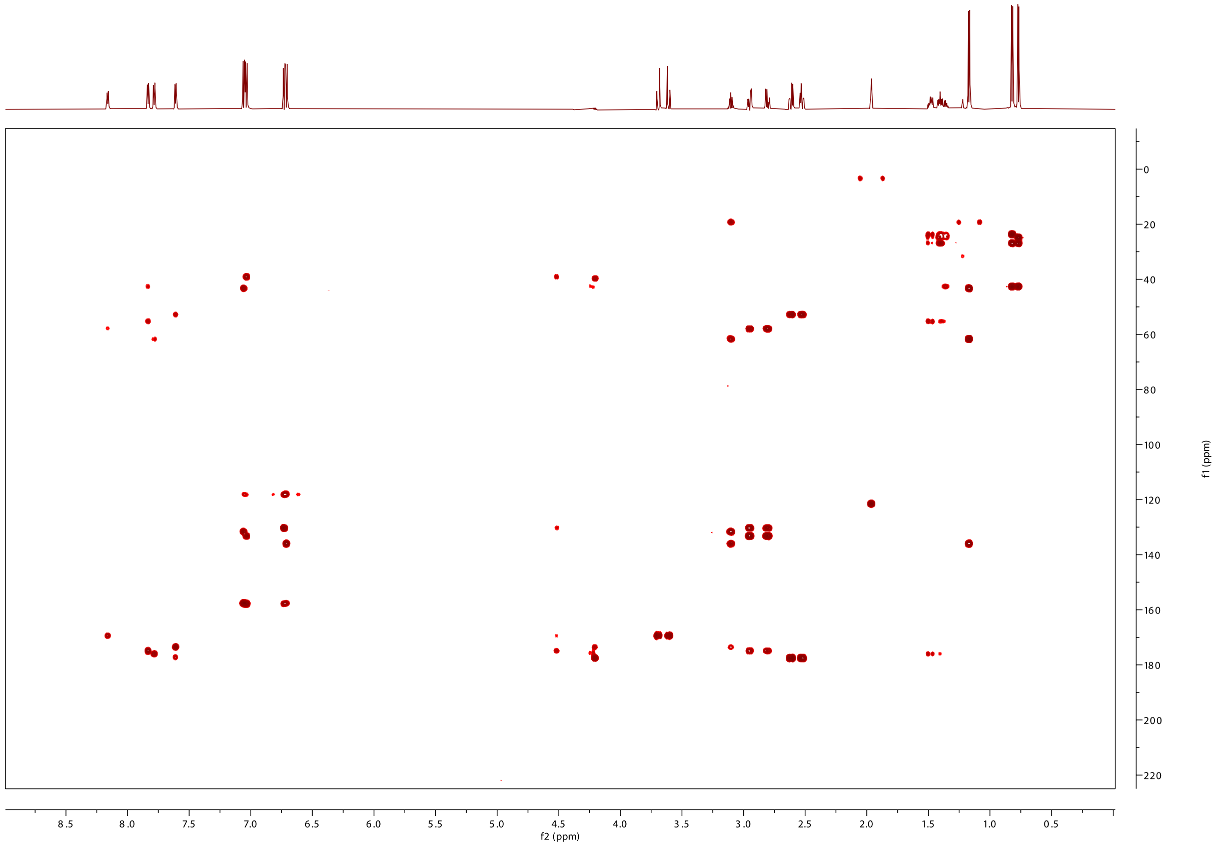

e) ^{1}H-^{13}C HMBC key cross peaks to assign the structure to β-methyltyrosine. See also Figure 3 (main text).

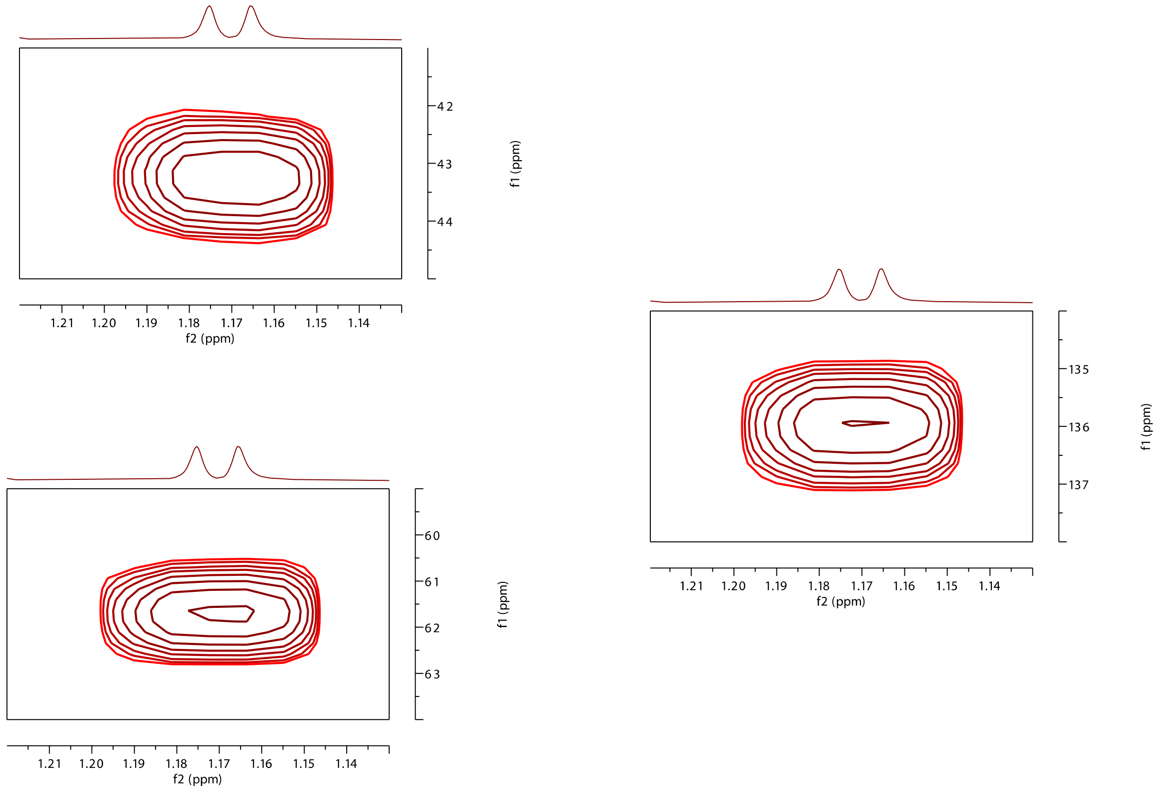

Table S4: Summary of ^{1}H and ^{13}C chemical shifts of every residue in BthAD-modified product after GluC cleavage.

residue number	AA	NH/C=O	αH/C	βH/C	γH/C	δH/C and others
1	Gly	169.4	3.69, 3.62<br>43.3			
2	Tyr	8.16<br>175.0	4.50<br>58.0	2.94, 2.80<br>39.2	136.0	130.3<br>7.04, 133.5<br>6.73, 118.4<br>C-OH: 157.6
3	Leu	7.83<br>176.0	4.23<br>55.3	1.48, 1.40<br>42.7	1.36<br>27.1	0.82, 25.1<br>0.77, 23.7
4	Tyr	7.78<br>173.6	4.42<br>61.6	3.10<br>43.1	1.17<br>19.3	135.9<br>7.06, 131.6<br>6.71, 118.0<br>C-OH: 158.0
5	Asp	7.61<br>177.2	4.20<br>52.8	2.61, 2.52<br>39.8		177.6

Figure S15: Marfey's analysis of BthAD-modified product. A. Marfey's derivatization scheme to determine the stereochemistry of β-methyltyrosine. B. Extracted ion chromatogram (EIC m/z = 700.1937) of hydrolyzed BthA and subsequently derivatized β-methyltyrosine using L-FDAA as the derivatization reagent. Commercial isomers of β-methyltyrosine were derivatized in the same manner and co-injected with the BthA-derived material. Results using D-FDAA are shown in Figure 3D (main text). In this experiment, treatment of modified BthA and standards in 6 M DCL resulted in exchange of the ortho protons of the Tyr residues with solvent.[7,8] DCl is used such that any potential epimerization can be detected by incorporation of additional deuterium.

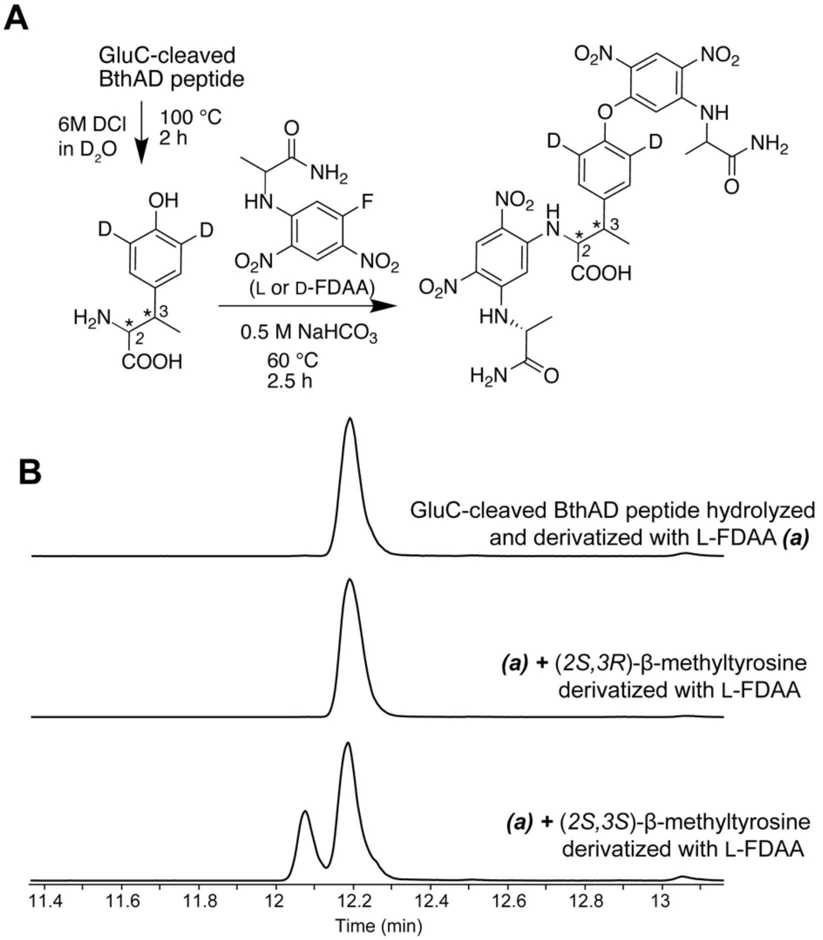

Figure S16: NMR spectra of BthAO-modified product after GluC cleavage. The utilized solvent was 10% D_2O/90% H_2O.

a) Depiction of structural elucidation

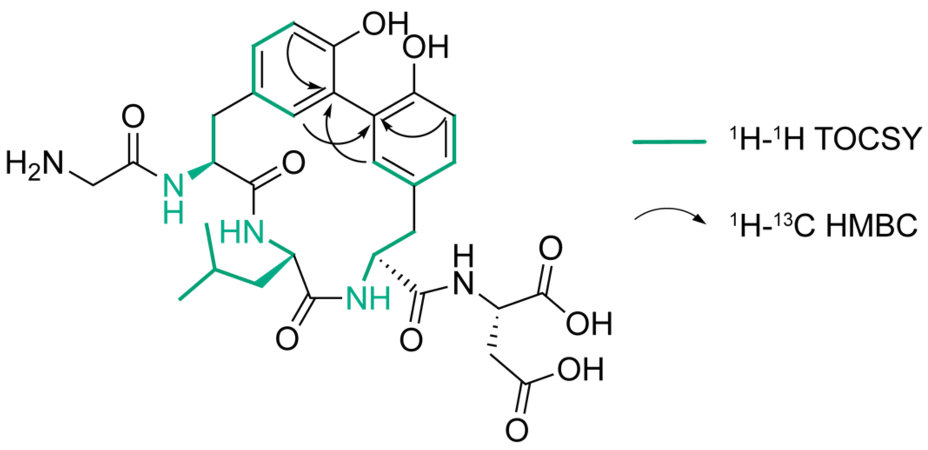

b) ^{1}H-NMR spectrum. The major singlet at 1.76 ppm is residual ammonium acetate from HPLC purification.

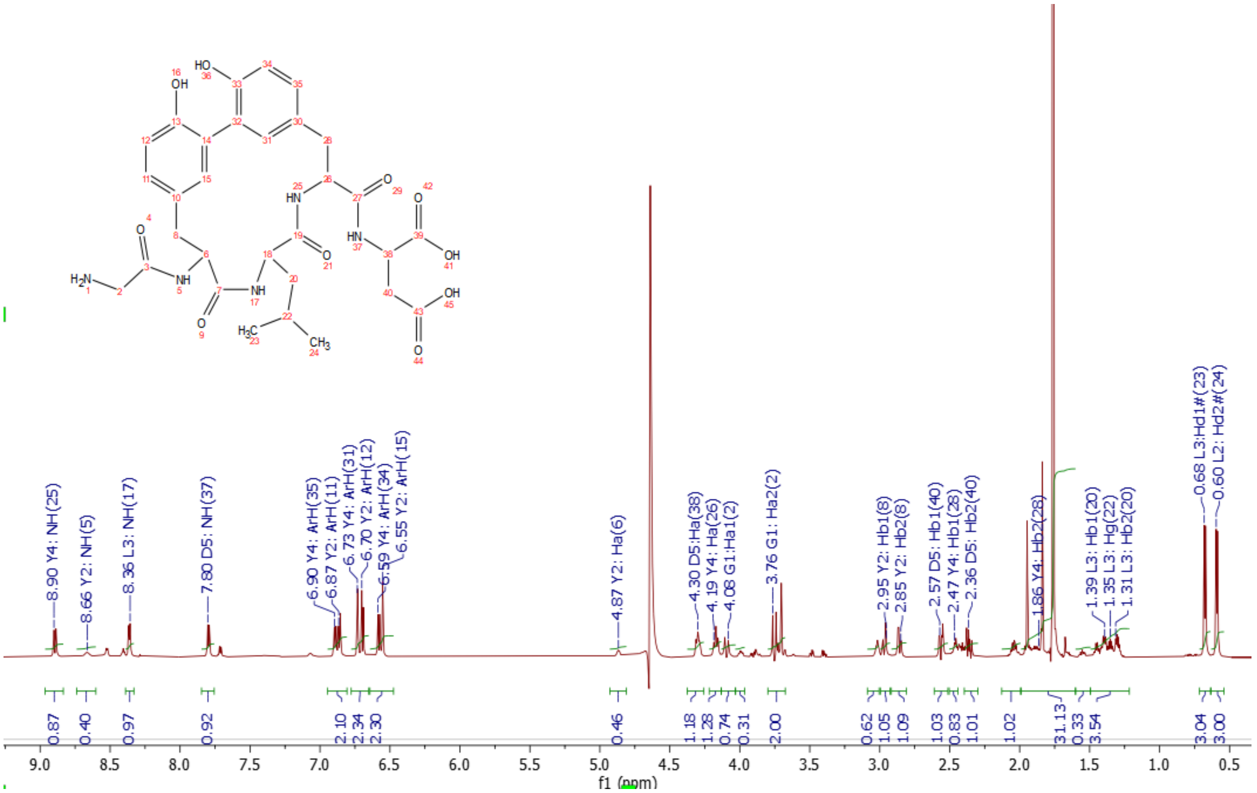

c) ^{1}H-^{1}H TOCSY

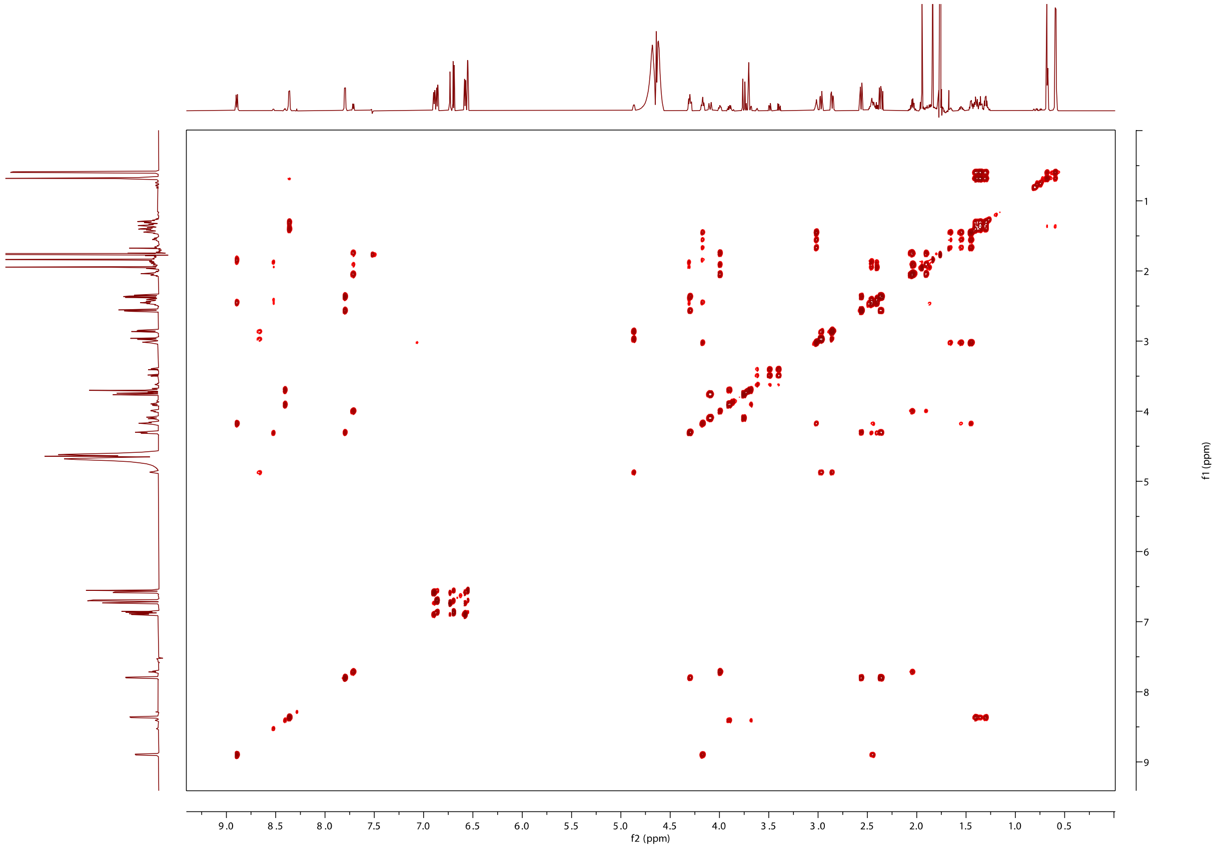

d) ^{1}H-^{1}H NOESY

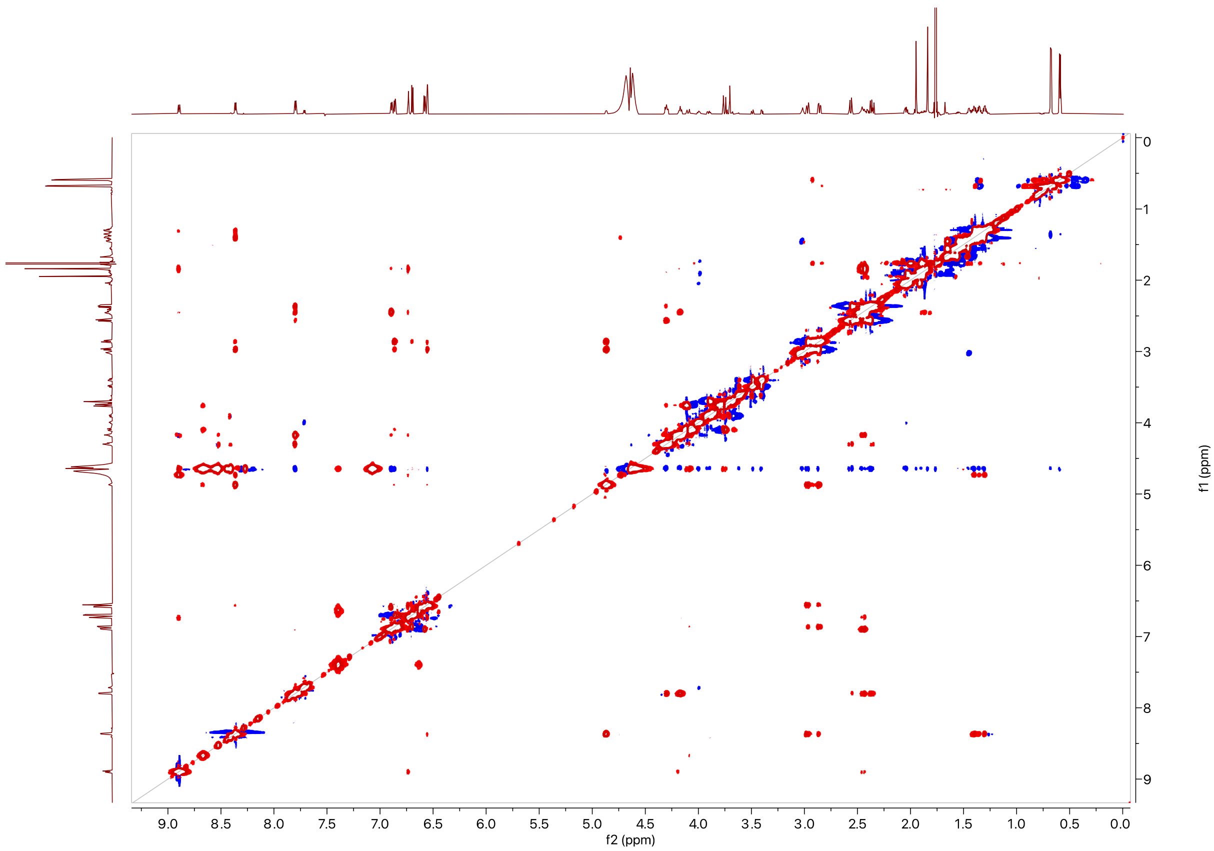

0
1
2
3
4
5
6
7
8
9
f1 (ppm)
9.0
8.5
8.0
7.5
7.0
6.5
6.0
5.5
5.0
4.5
4.0
3.5
3.0
2.5
2.0
1.5
1.0
0.5
0.0
f2 (ppm)

e) ^{1}H-^{13}C HSQC

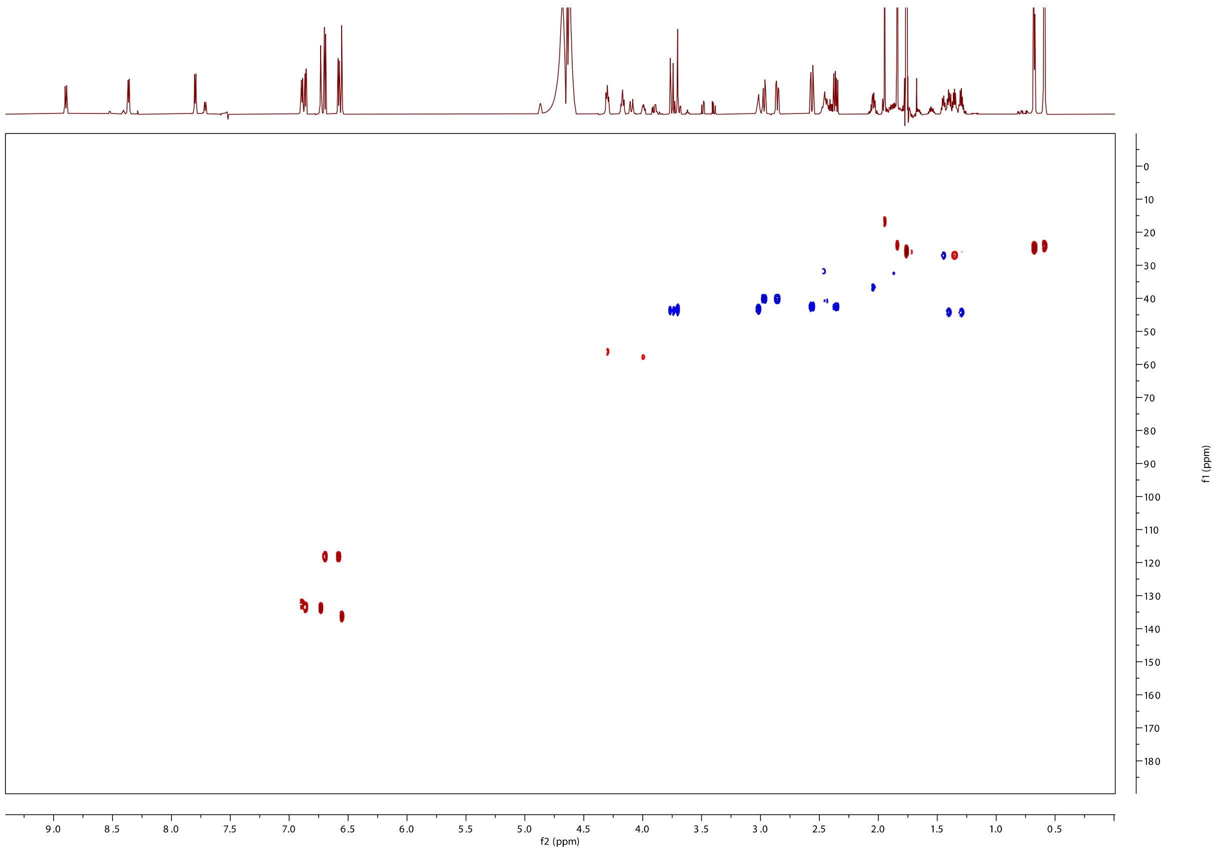

f) ^{1}H-^{13}C HMBC

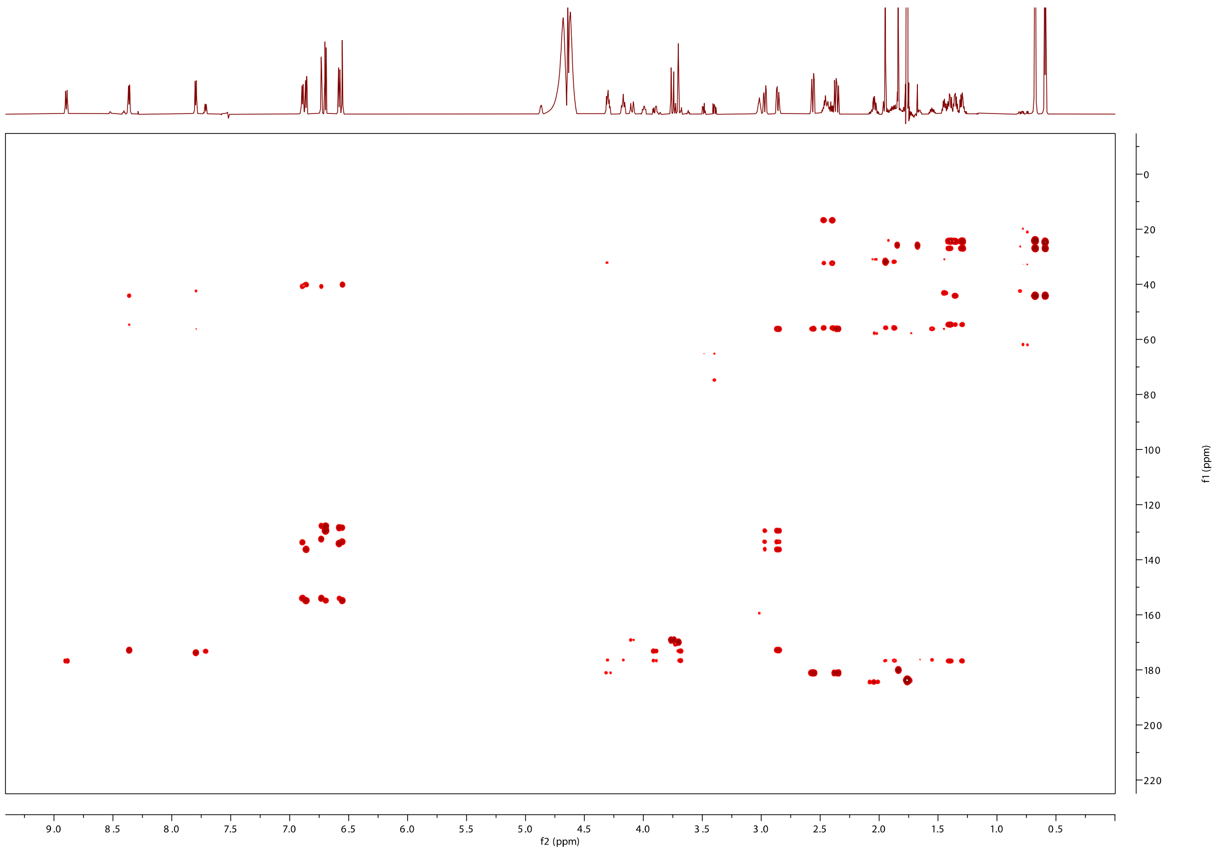

g) Assignment of amino acids using ^{1}H-^{1}H TOCSY data

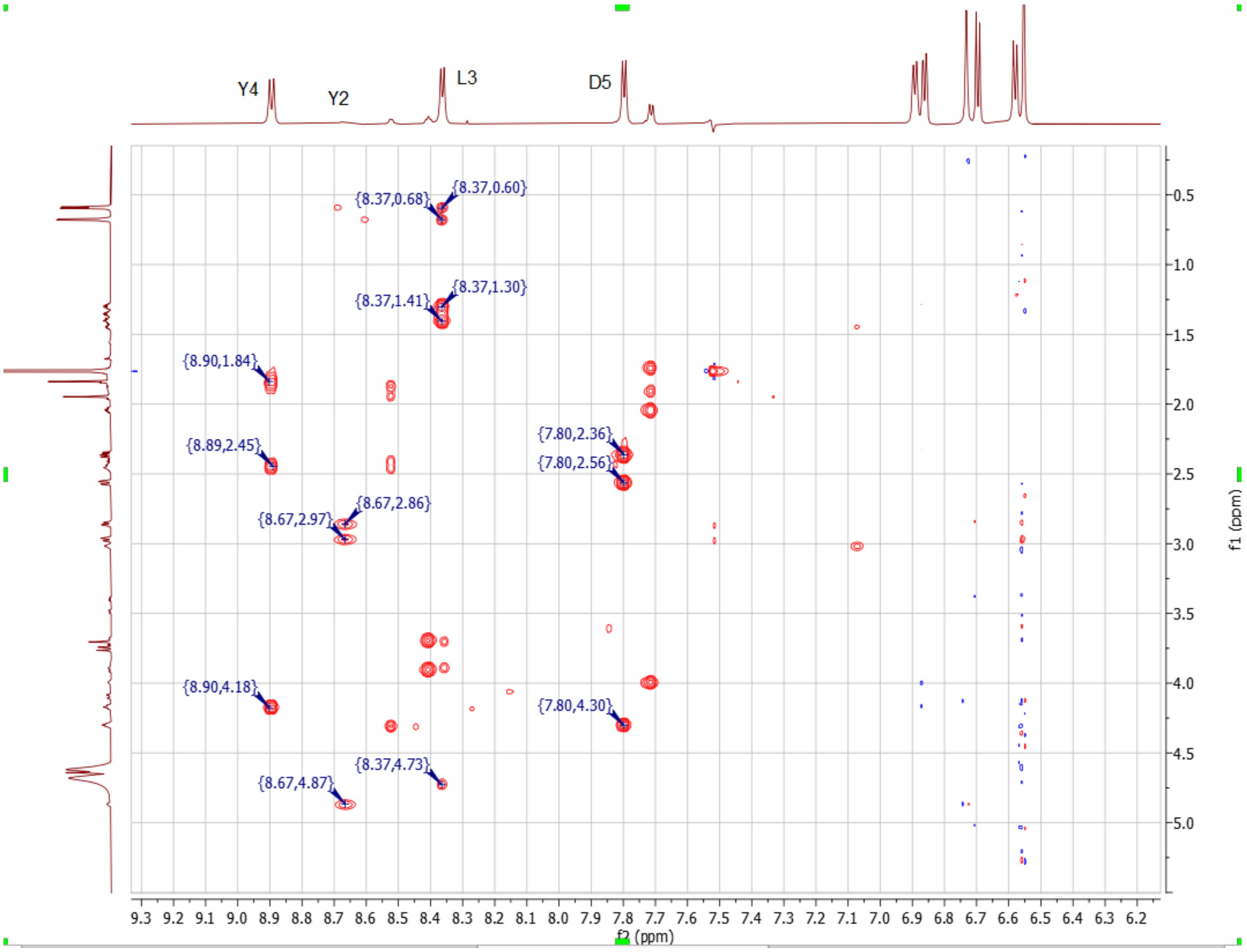

h) Annotated ^{1}H-^{1}H NOESY correlation

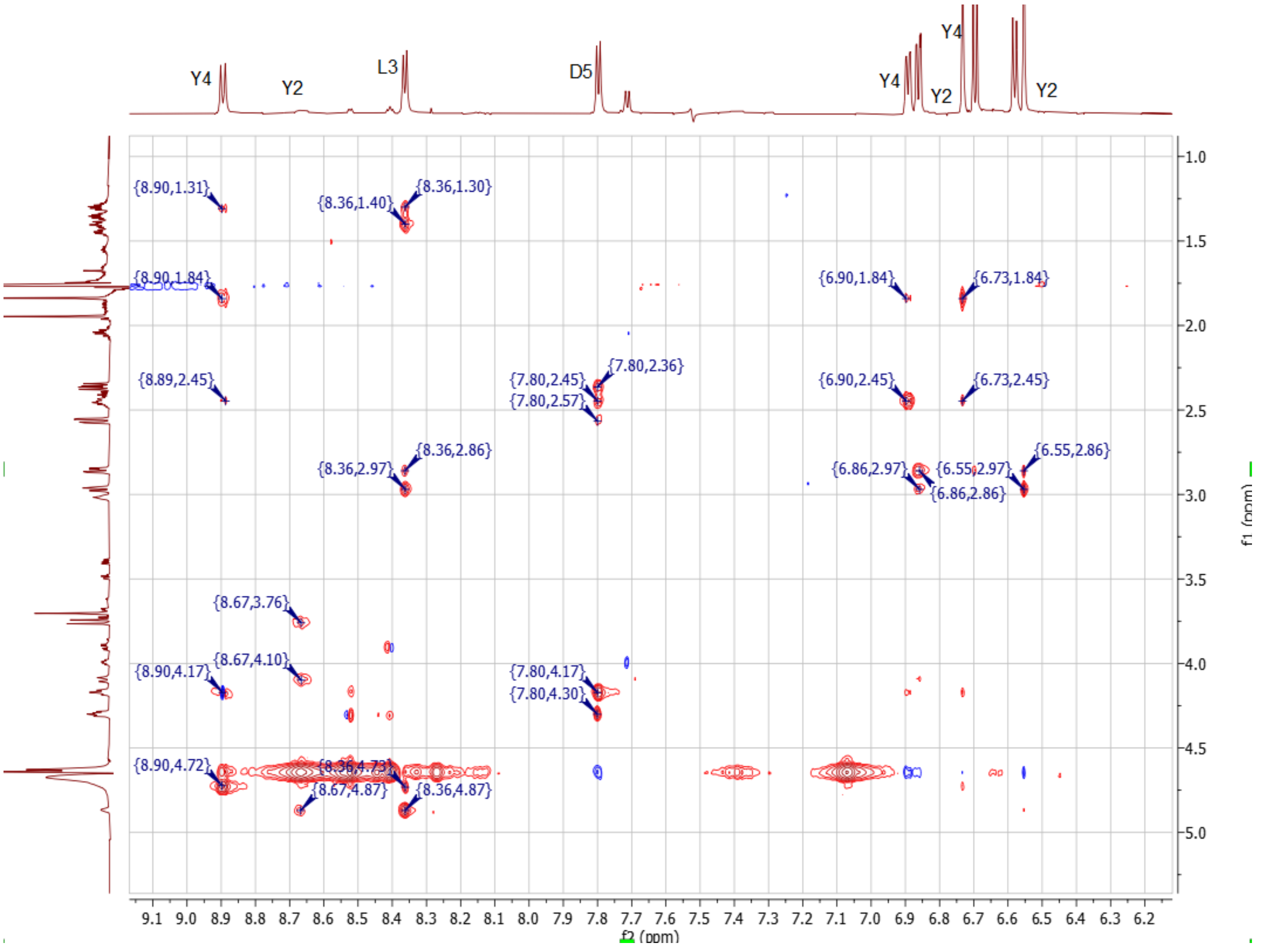

i) Key ^{1}H-^{13}C HMBC correlations for structural elucidation.

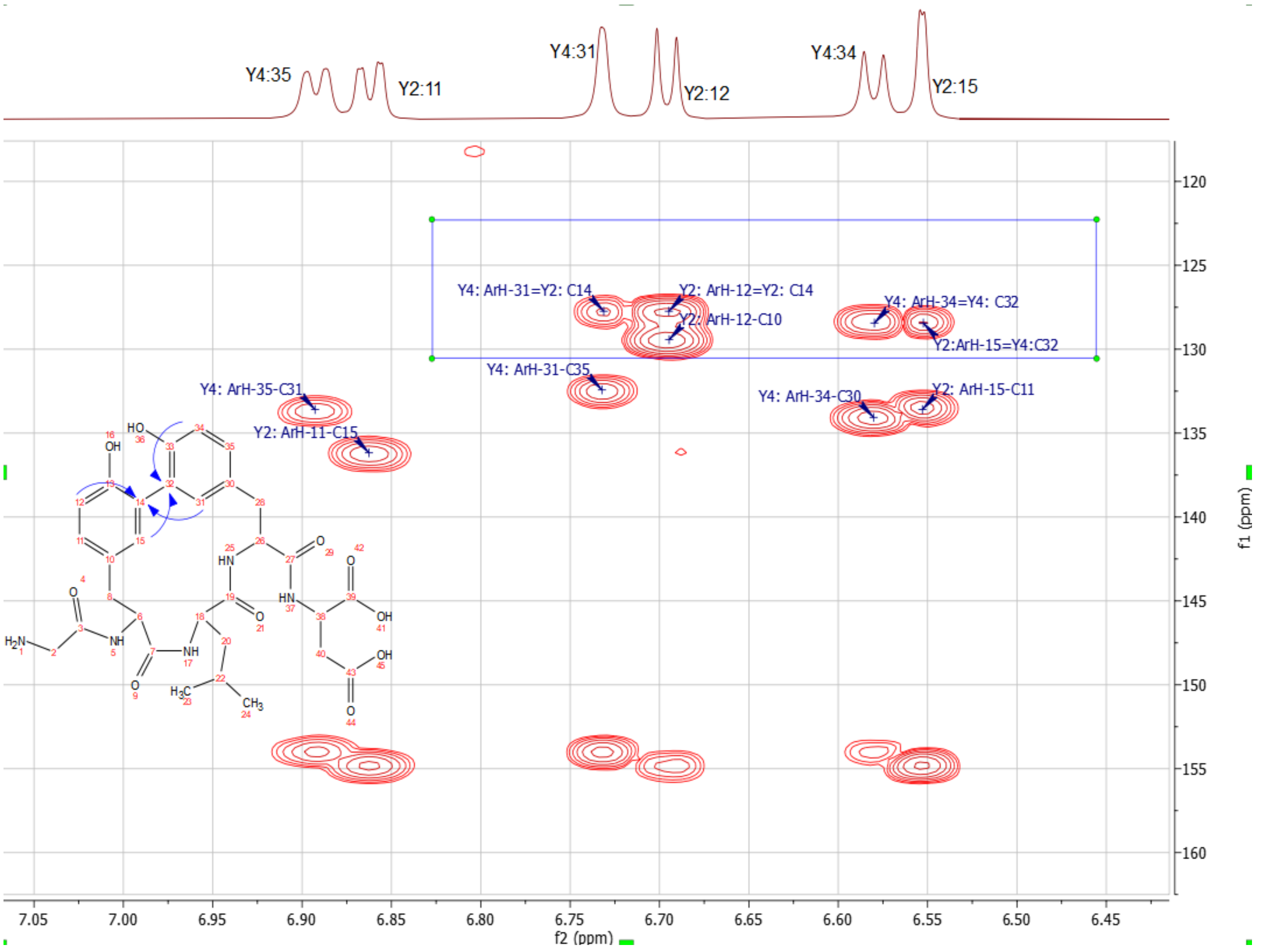

Table S5: Summary of ^{1}H and ^{13}C chemical shift of every residue in BthAO-modified product after GluC cleavage.

residue number	AA	NH/C=O	αH/C	βH/C	γH/C	δH/C and others	
1	Gly	169.2	3.75, 3.70<br>43.3				
2	Tyr	8.67<br>172.8	4.87<br>56.2	2.97, 2.87<br>40.2	129.6	6.86 (dd), 133.4; 6.70 (d), 118.1; 6.55 (d, 2Hz), 136.3.<br>OH-C: 154.9, Ar-C (linker) 127.8	
3	Leu	8.36<br>176.8	4.72<br>54.9	1.40, 1.31<br>44.0	1.36<br>26.9	0.69, 24,6<br>0.60, 24.2	
4	Tyr	8.90<br>173.8	4.18<br>57.2	2.45, 1.85<br>40.8	134.1	6.89 (dd), 132.6; 6.58 (d), 118.1<br>6.73(d, 2Hz), 133.8.<br>OH-C: 153.9; Ar-C (linker): 128.4	
5	Asp	7.80<br>181.0	4.29<br>56.0	2.57, 2.36<br>42.4		181.1	

Figure S17: NMR spectra of BthADO-modified product after GluC cleavage. The solvent used was 40% CD_3CN/60% H_2O. The structure of the product is shown below:

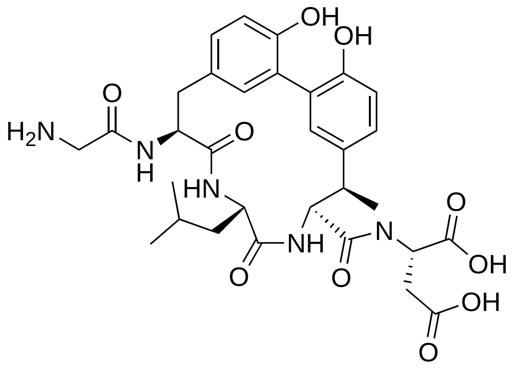

a) ^{1}H-NMR spectrum

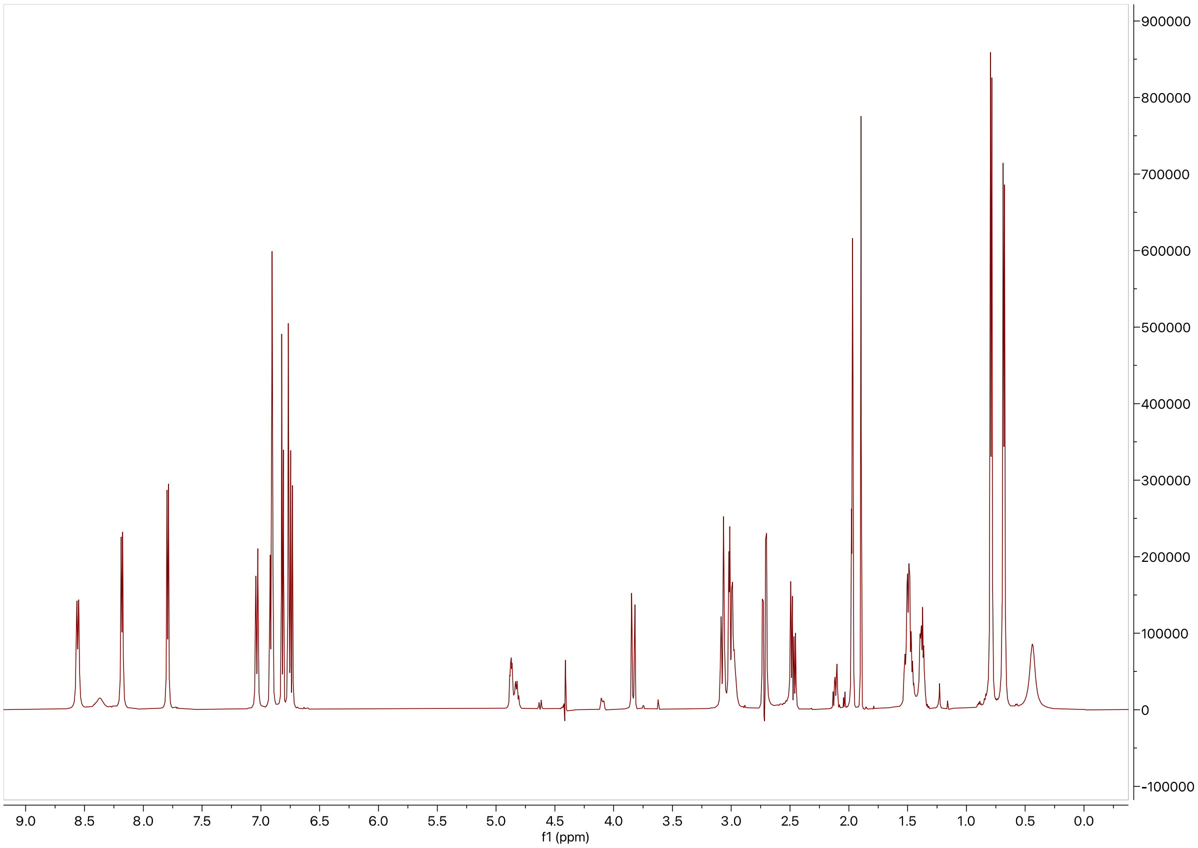

b) ^{1}H-^{1}H TOCSY

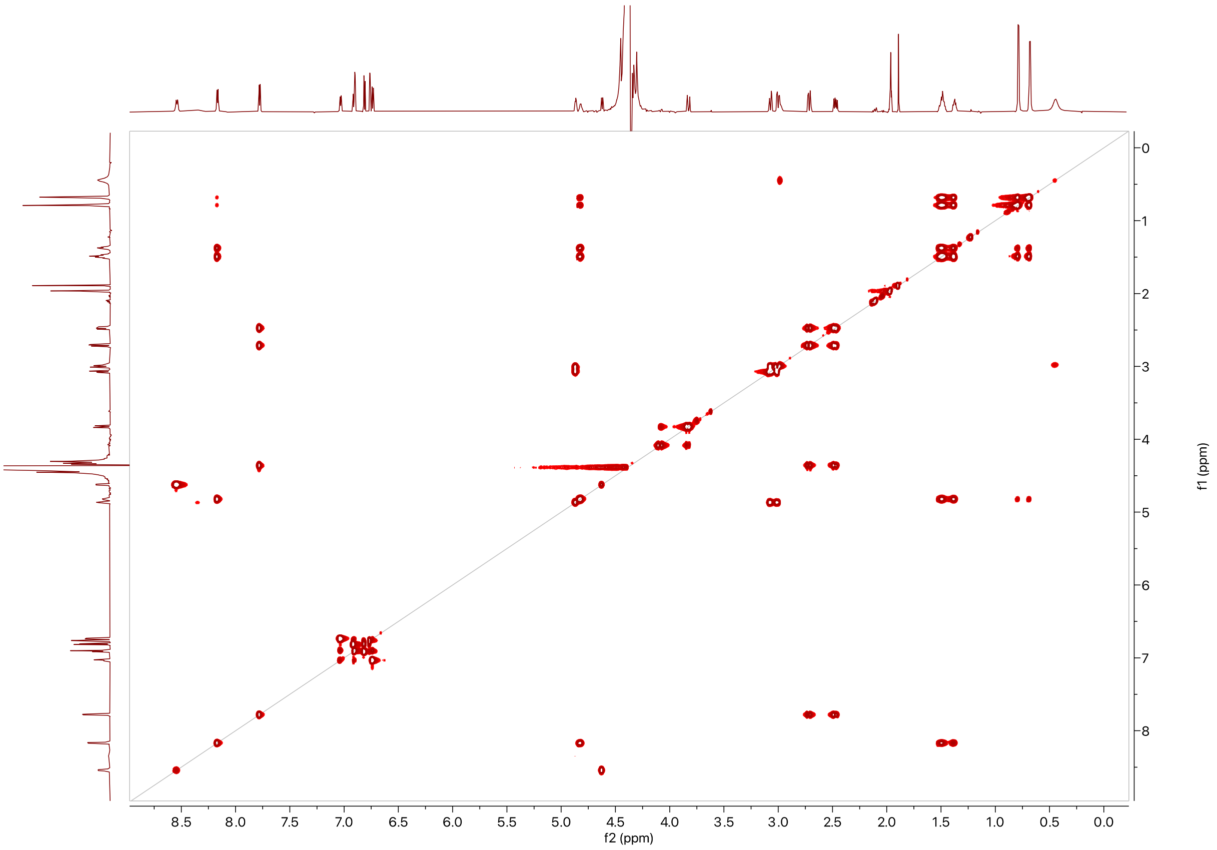

8.5
8.0
7.5
7.0
6.5
6.0
5.5
5.0
4.5
4.0
3.5
3.0
2.5
2.0
1.5
1.0
0.5
0.0
f2 (ppm)
0
1
2
3
4
5
6
7
8
f1 (ppm)

c) ^{1}H-^{1}H NOESY

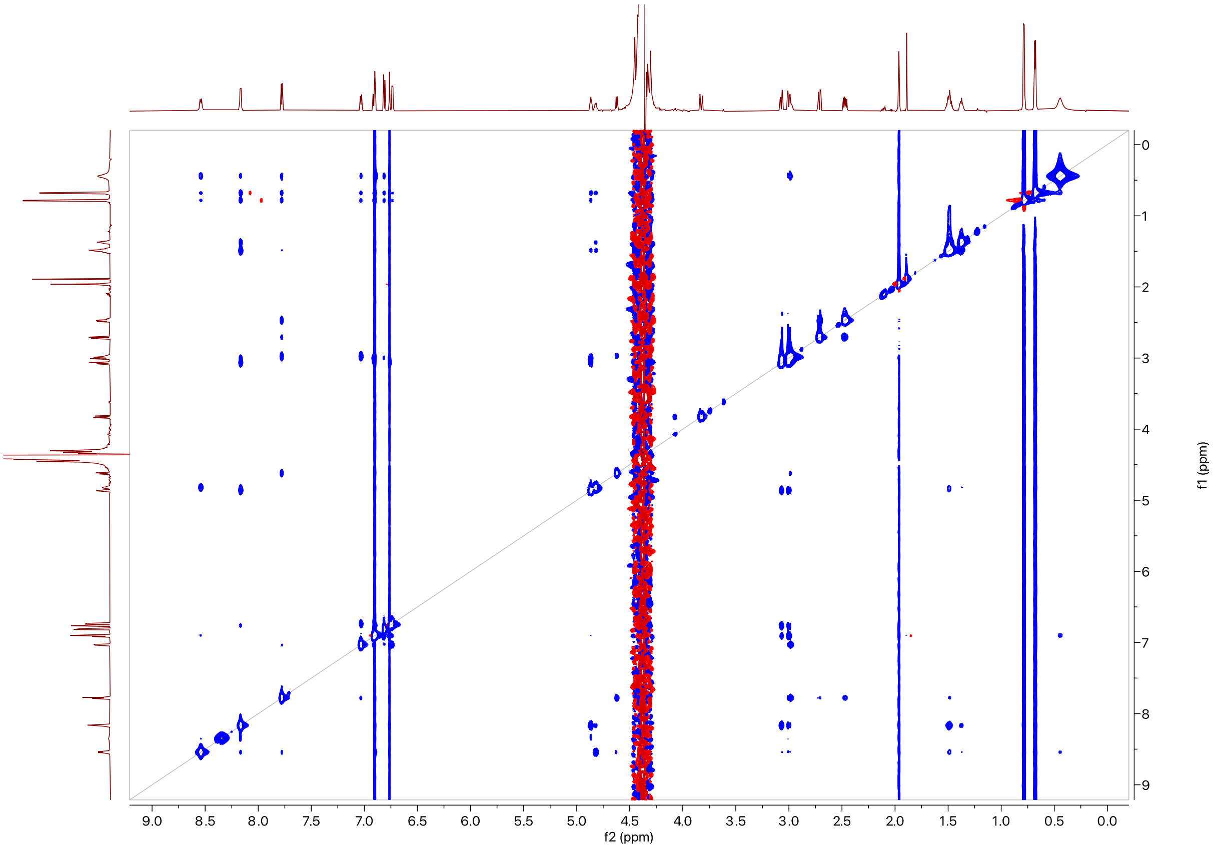

d) ¹H-¹³C HSQC

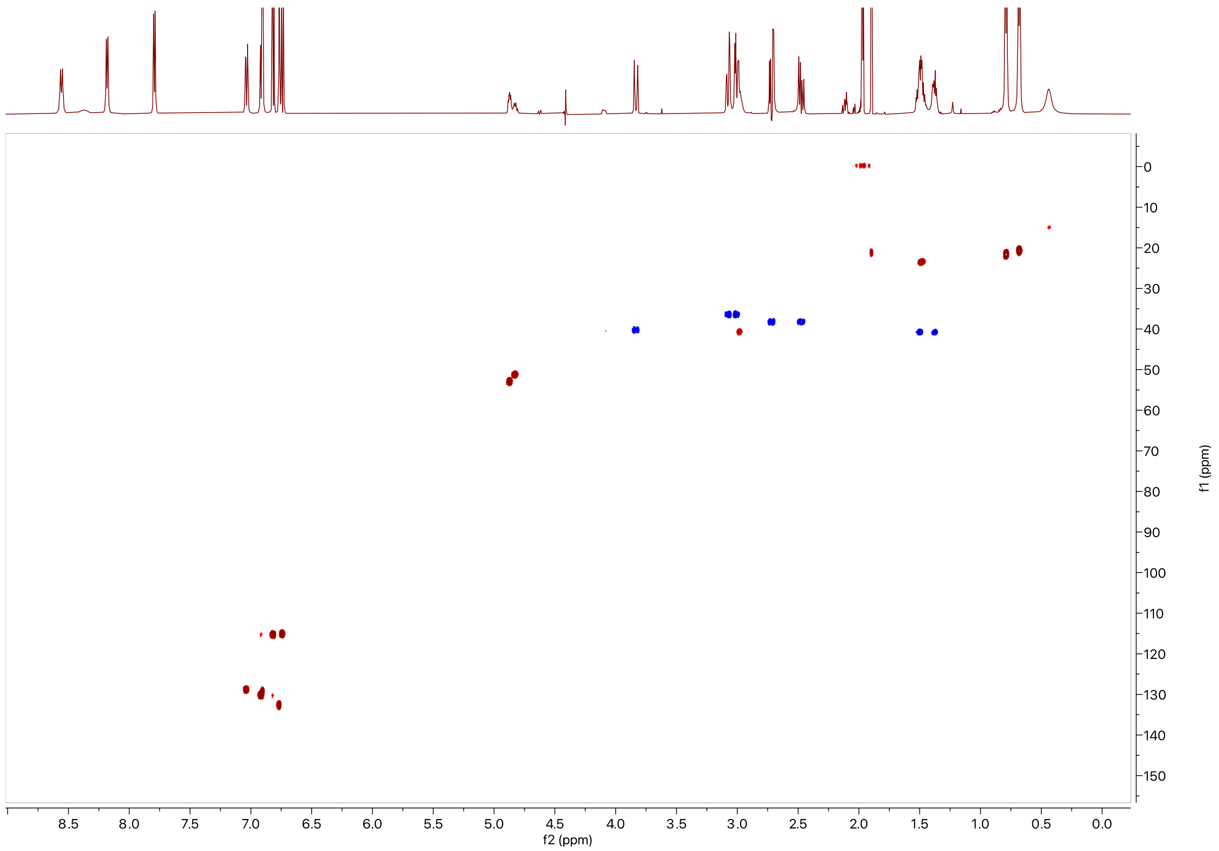

e) ^{1}H-^{13}C HMBC

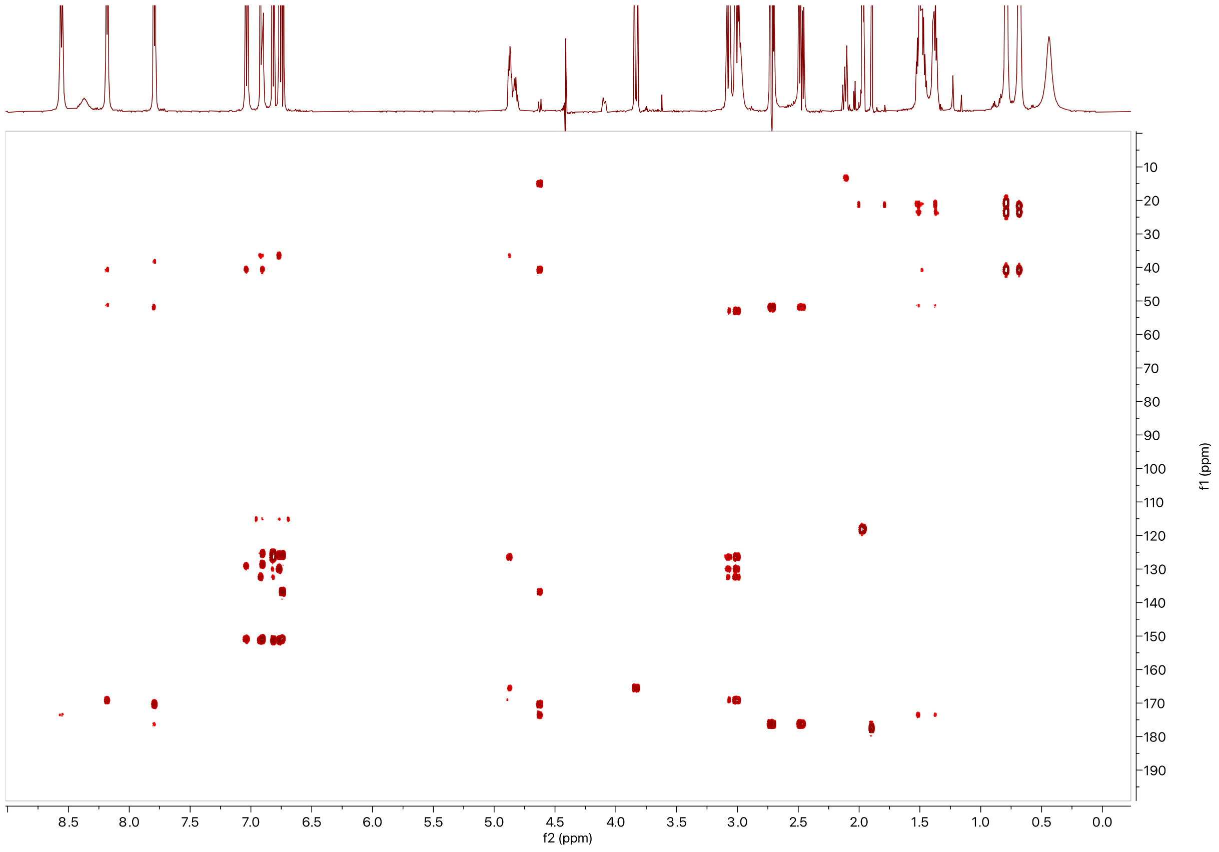

Table S6: Summary of ^{1}H and ^{13}C chemical shifts of every residue in the BthAO-modified product after GluC cleavage.

number	AA	NH/C=O	αH/C	βH/C	γH/C	δH/C and others
1	Gly	 167.2	4.08, 3.83<br>42.1			
2	Tyr	8.35 (br)<br>170.8	4.87<br>54.8	3.08, 3.01<br>38.2	 127.1	6.92(dd), 131.8; 6.82(d), 117.0<br>6.77(d, 2Hz), 134.3<br>OH-C: 153.0; C-Ar (linker): 128.3
3	Leu	8.17<br>175.1	4.83<br>53.0	1.51, 1.38<br>42.6	1.51<br>25.3	0.79, 23.4<br>0.69, 22.4
4	Tyr	8.54<br>172.1	4.63<br>57.6	3.00 (br)<br>42.4	0.45 (br)<br>16.8	138.5 (γ)<br>7.04(dd), 130.3; 6.74(d), 116.8<br>6.91(d, 2Hz), 131.1<br><br>OH-C: 152.7; C-Ar (linker): 127.6
5	Asp	7.78<br>N/A	4.36<br>53.6	2.72, 2.47<br>40.0		178.0 (γ)

Figure S18: Exciton-coupled circular dichroism (ECD) spectra of synthetic standards.

a) (*R*)-(+)-5,5′,6,6′,7,7′,8,8′-octahydro-1,1′-bi-2-naphthol

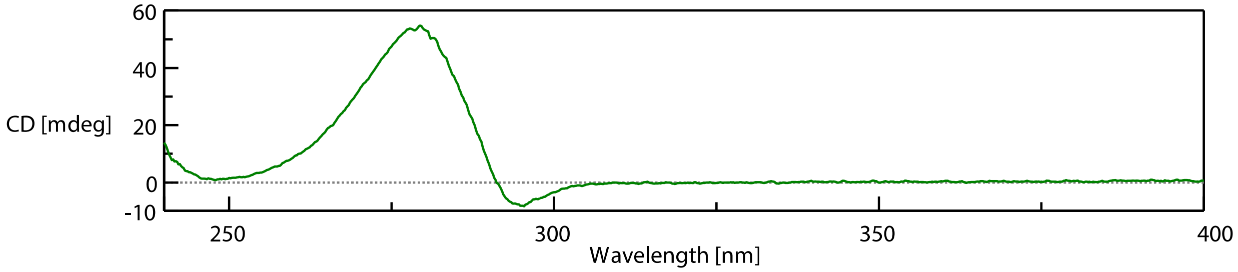

b) (*S*)-(-)-5,5′,6,6′,7,7′,8,8′-octahydro-1,1′-bi-2-naphthol

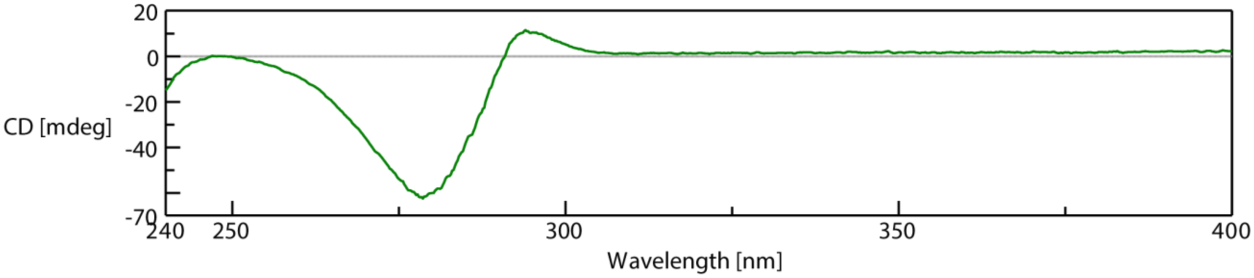

c) (*R*)-(+)-5,5',6,6'-Tetramethyl-3,3'-di-t-butyl-1,1'-biphenyl-2,2'-diol

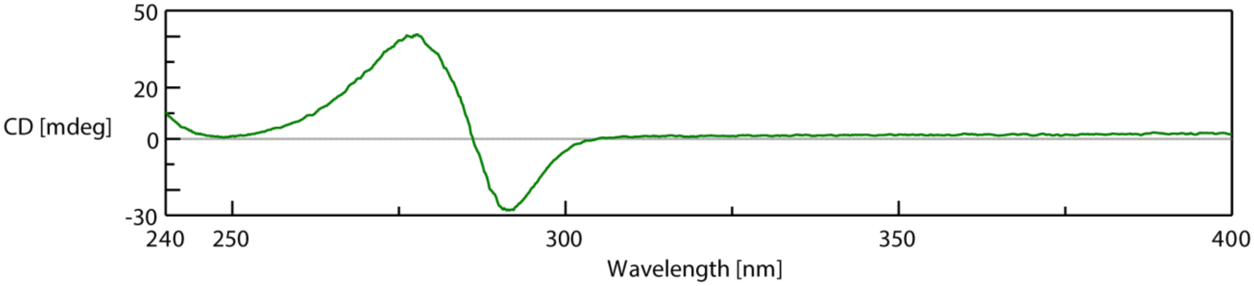

d) (*S*)-(-)-5,5',6,6'-Tetramethyl-3,3'-di-t-butyl-1,1'-biphenyl-2,2'-diol

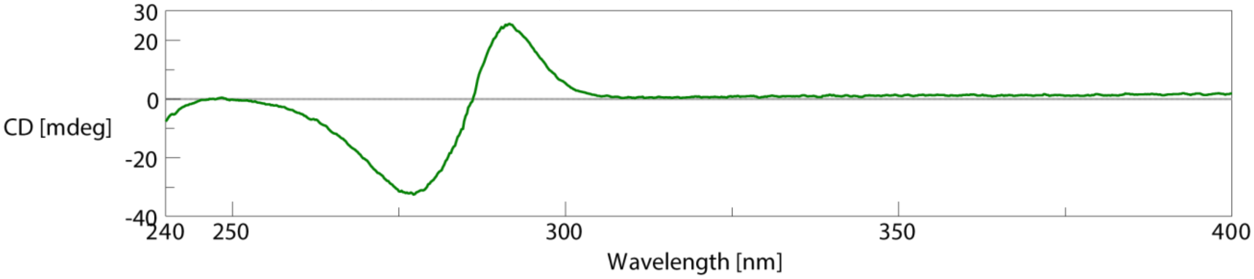

Figure S19: ECD spectrum (top) and UV spectrum (bottom) of the GluC-cleaved, BthAO-modified product. Abs: absorbance.

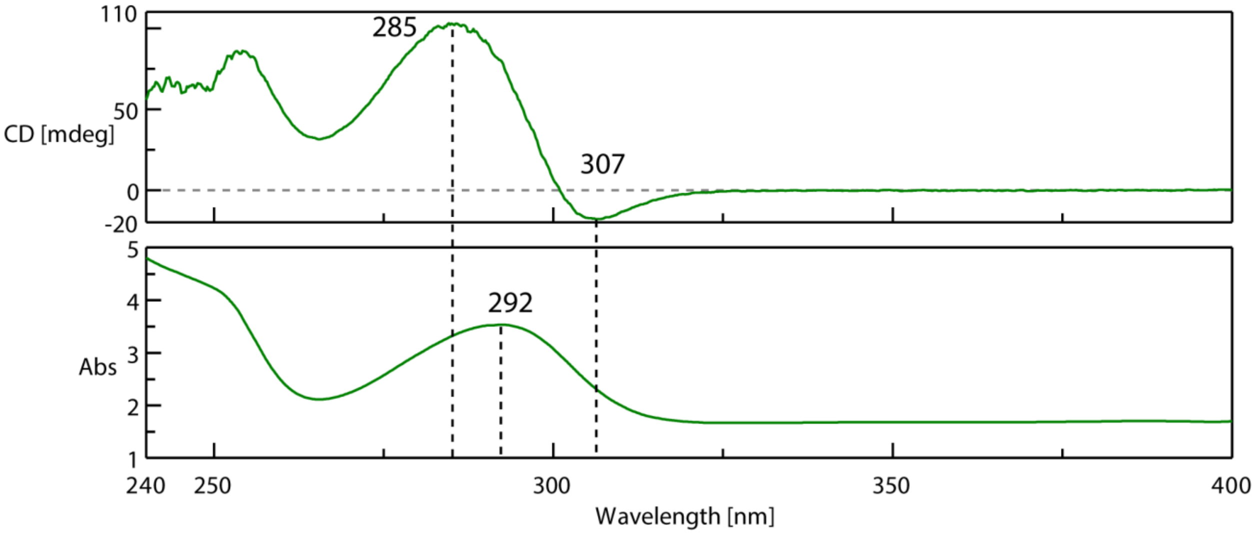

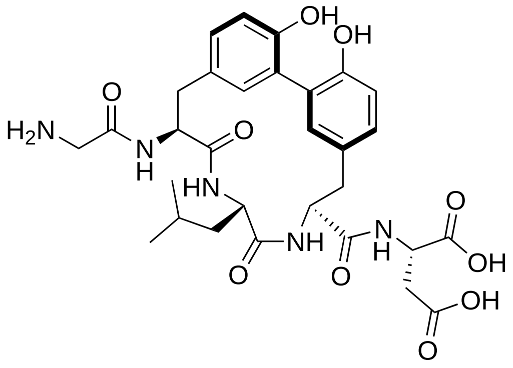

Figure S20: ECD spectrum (top) and UV spectrum (bottom) of the GluC-cleaved, BthAD-modified product, which does not contain the biaryl crosslink. Abs: absorbance

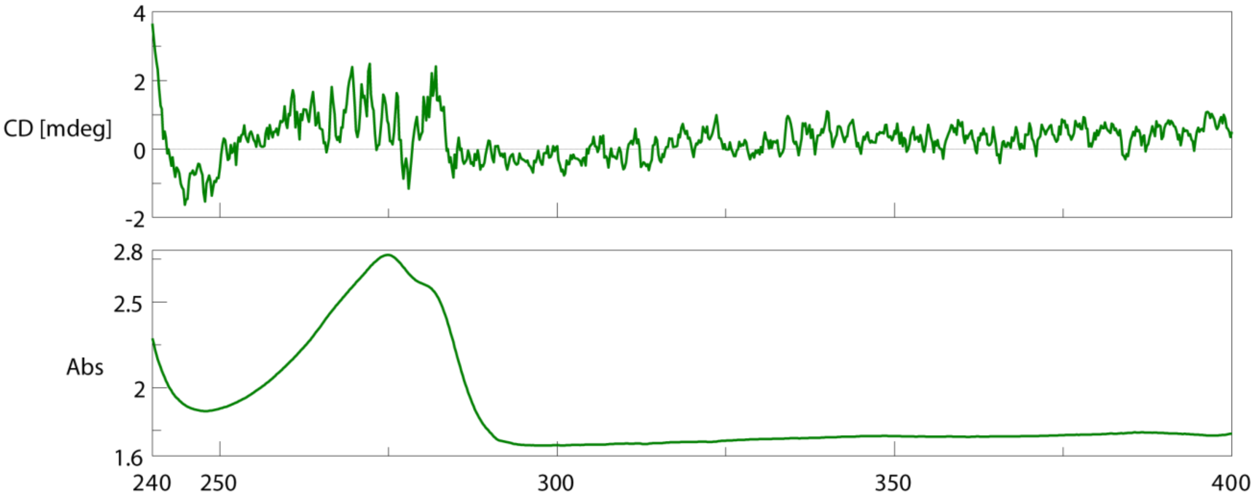

Figure S21: Deconvoluted HR-ESI mass of BthA coexpressed with modifying enzymes (BthO removed) in *Burkholderia* sp. FERM BP-3421 using the initial pSCrhaB2 construct (Table S2). The sequence of the peptide is shown below with the cut site bolded (endoproteinase GluC was used). The removal of BthO abolished BthHI activity but enabled BthD activity. Calculated exact mass for GluC-cleaved BthA: 629.2696 Da. Calculated exact mass for GluC-cleaved BthD-modified BthA: 643.2852 Da.

MHHHHHHMATKPKKTKGVSVSIEGKLPKMTLDMPVDAKKIKAIQKCLENGKLTITMSKVDLAGG
RMG**E**GYLYD

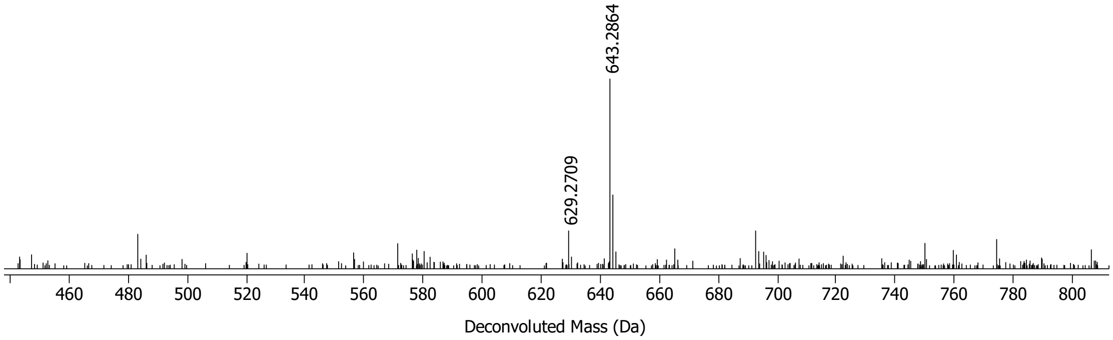

Figure S22: HR-ESI-MS/MS spectrum of BthA coexpressed with modifying enzymes (BthO removed) in *Burkholderia* sp. FERM BP-3421 using the initial pSCrhaB2 construct (Table S2). The removal of BthO abolished BthHI activity but enabled BthD activity.

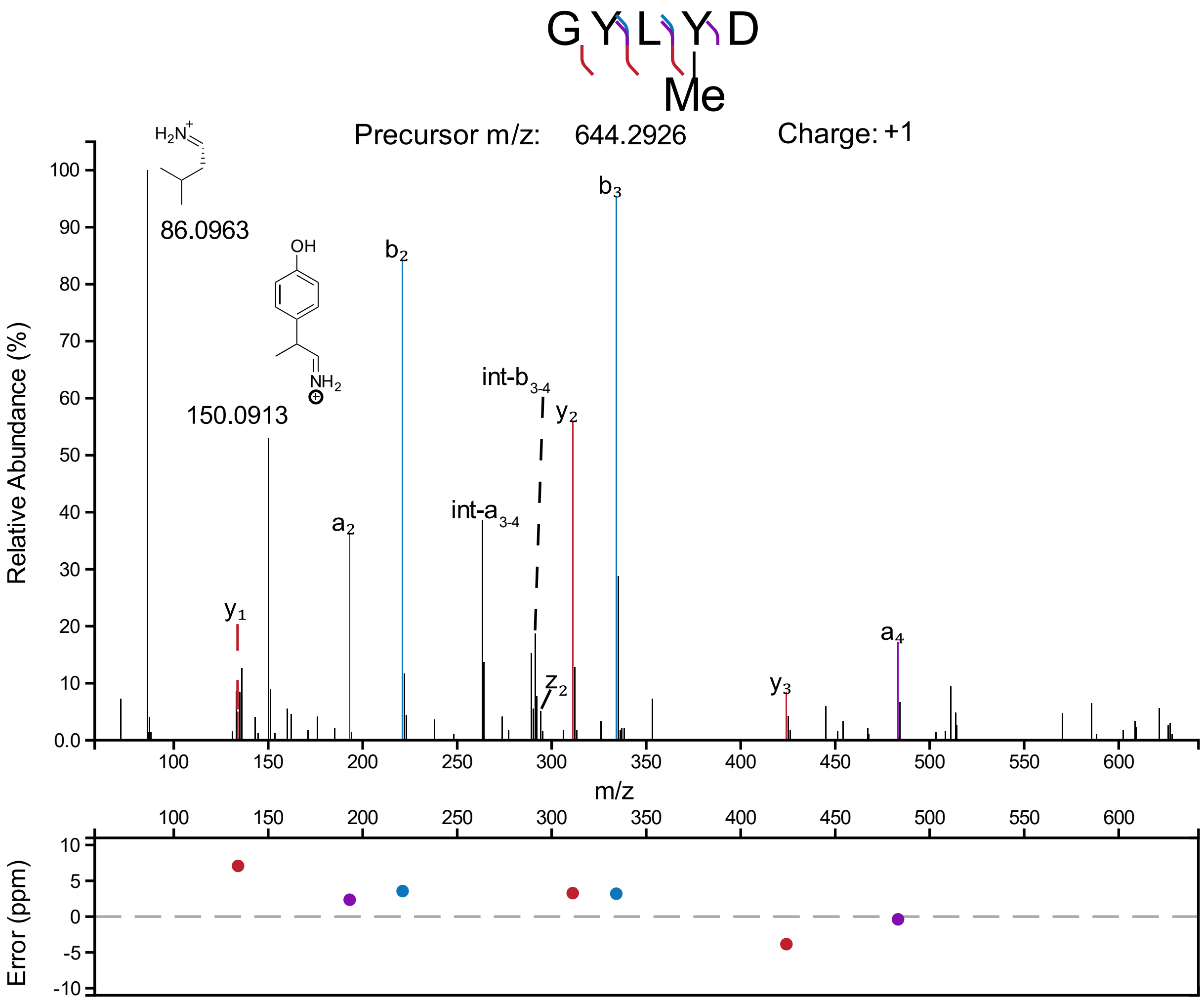

Figure S23: NMR spectra of BthAOHIS-modified product after GluC cleavage. The utilized solvent was 10% D_2O/ 90% H_2O. The structure of the product and key NMR depiction is in Figure 4 (main text).

a) ^{1}H NMR:

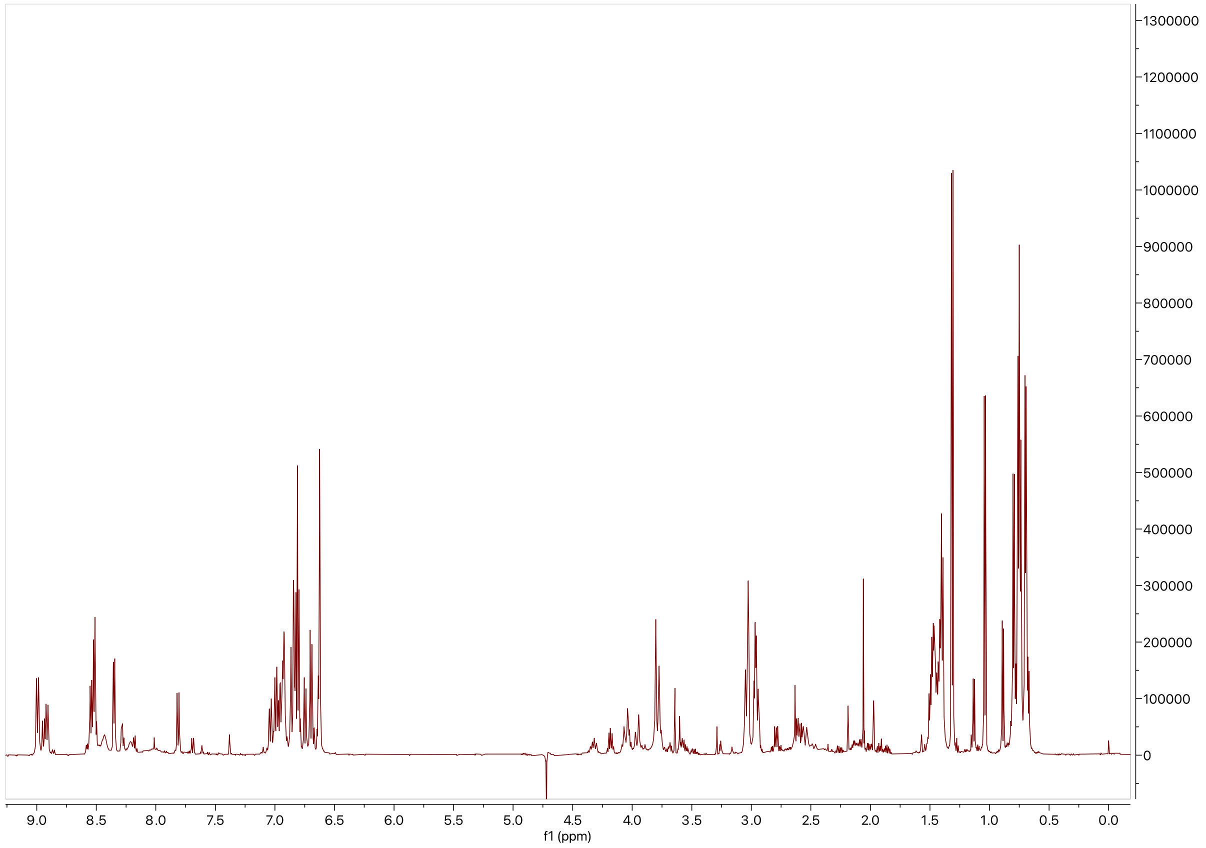

b) ¹H-¹H TOCSY

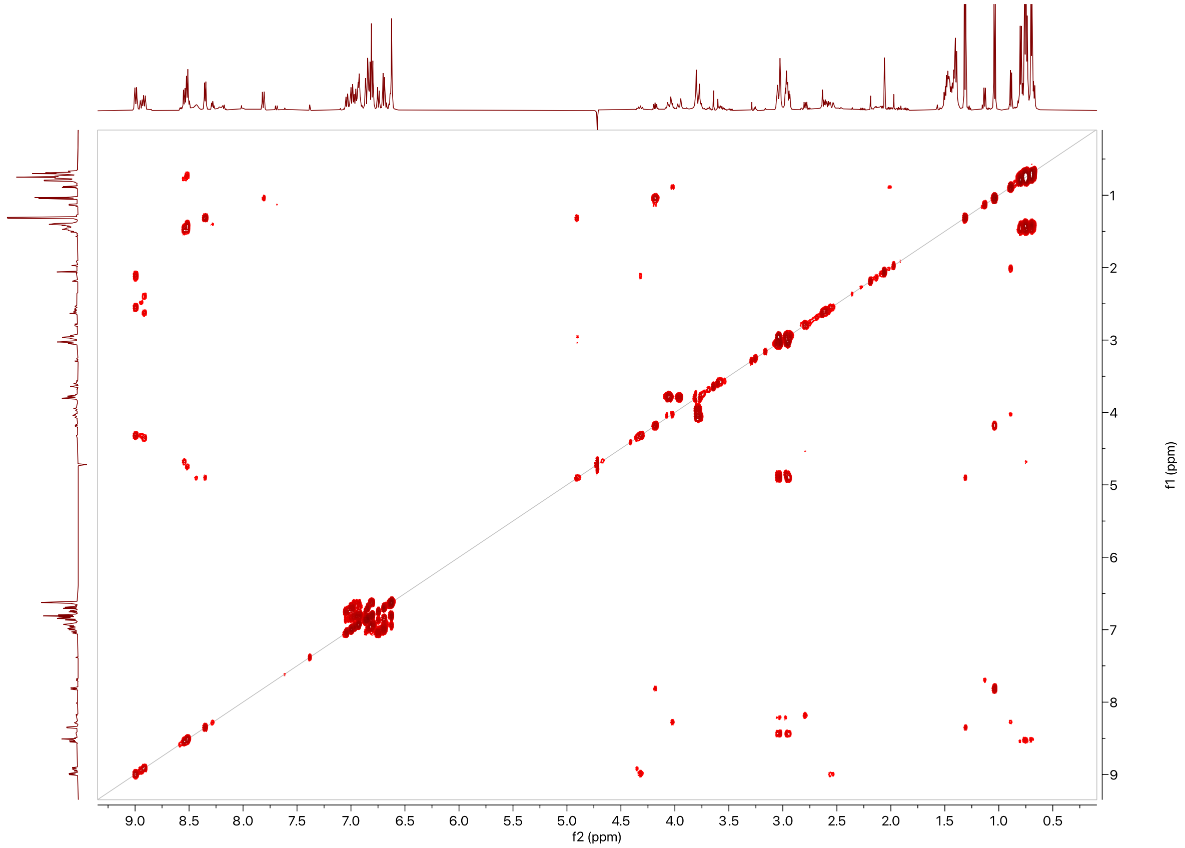

9.0
8.5
8.0
7.5
7.0
6.5
6.0
5.5
5.0
4.5
4.0
3.5
3.0
2.5
2.0
1.5
1.0
0.5
f2 (ppm)
1
2
3
4
5
6
7
8
9
f1 (ppm)

c) ^1H-^1H NOESY

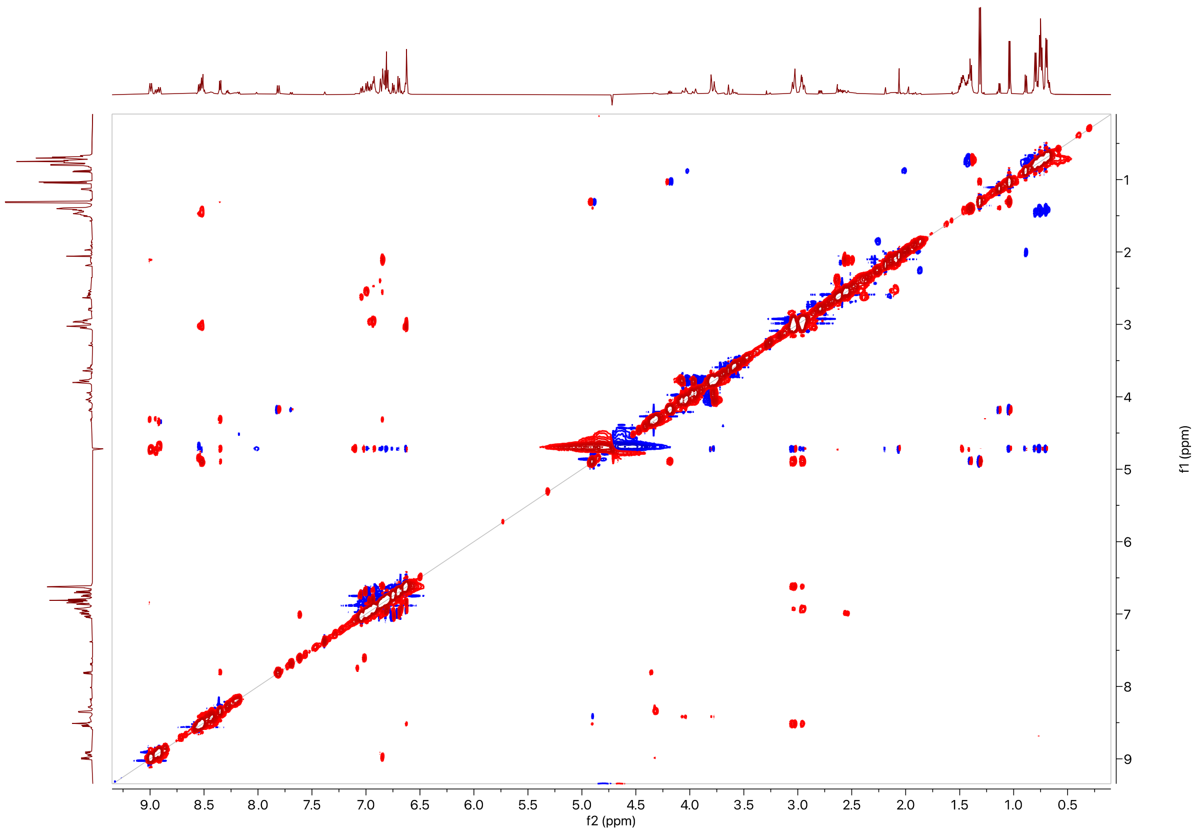

9.0
8.5
8.0
7.5
7.0
6.5
6.0
5.5
5.0
4.5
4.0
3.5
3.0
2.5
2.0
1.5
1.0
0.5
f2 (ppm)
1
2
3
4
5
6
7
8
9
f1 (ppm)

d) ^{1}H-^{1}H ROESY

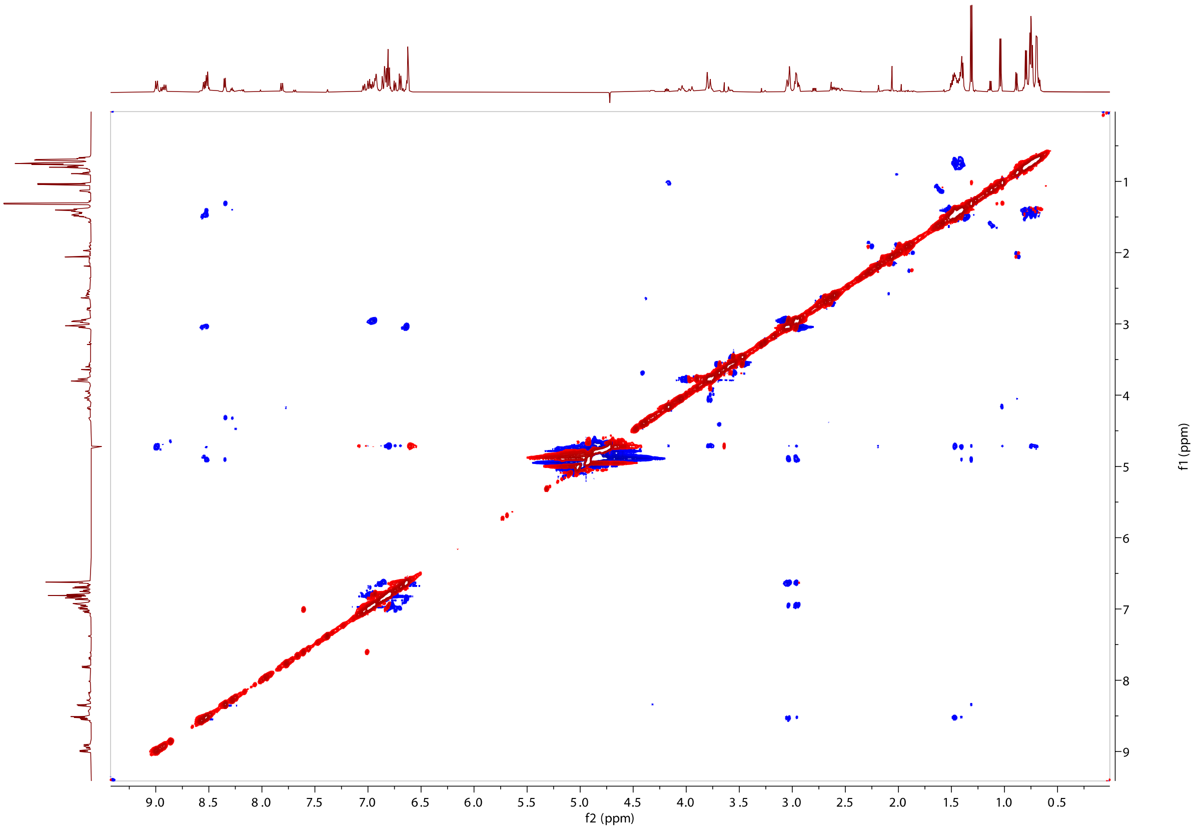

9.0
8.5
8.0
7.5
7.0
6.5
6.0
5.5
5.0
4.5
4.0
3.5
3.0
2.5
2.0
1.5
1.0
0.5
f2 (ppm)
1
2
3
4
5
6
7
8
9
f1 (ppm)

e) ^{1}H-^{13}C HSQC

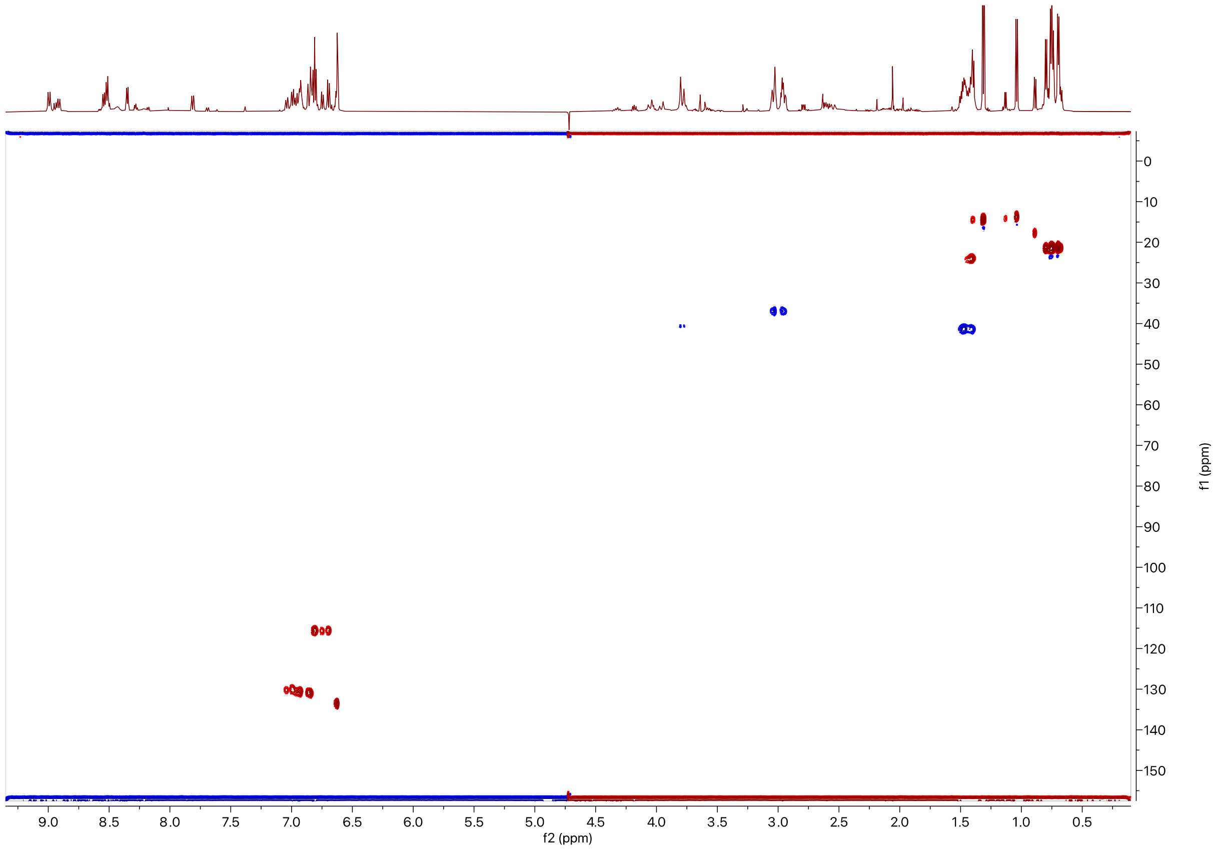

e) $^{1}H-^{13}C$ HMBC

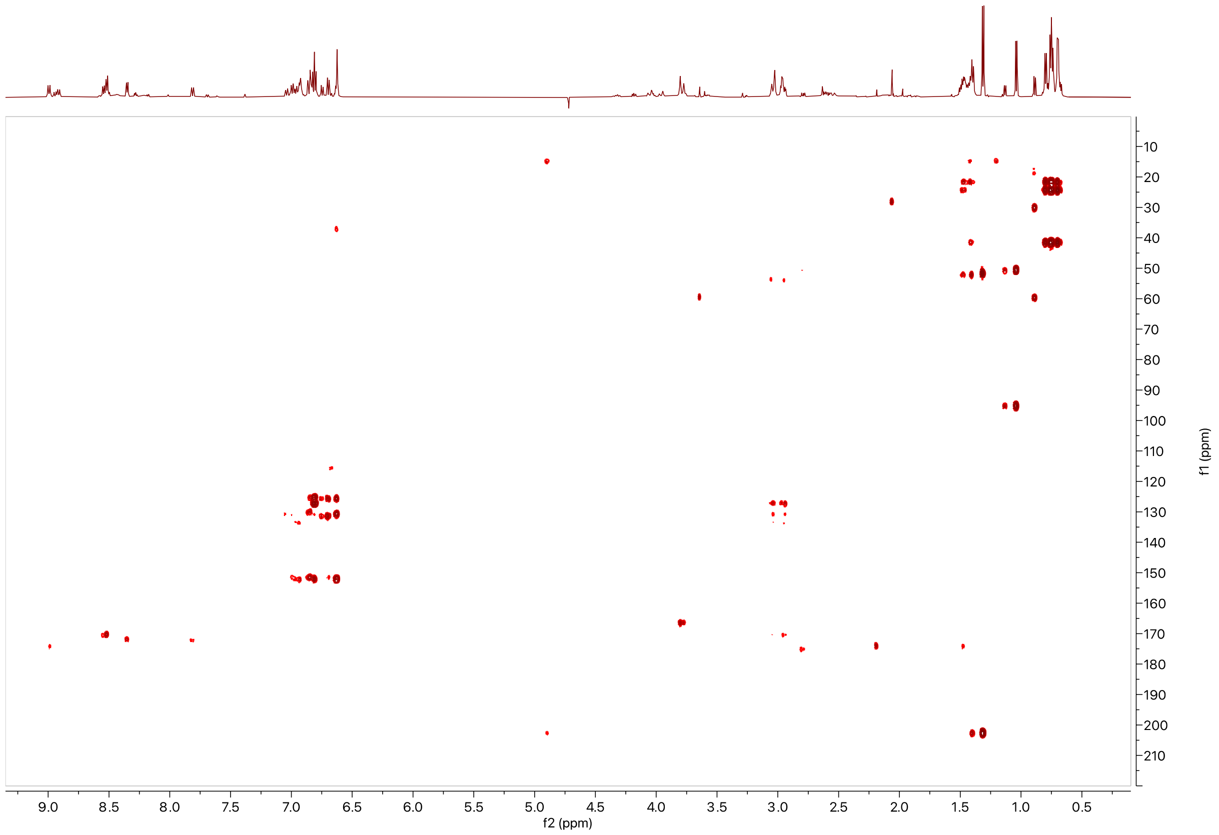

f) ^{1}H-^{1}H TOCSY of the diol form of the product

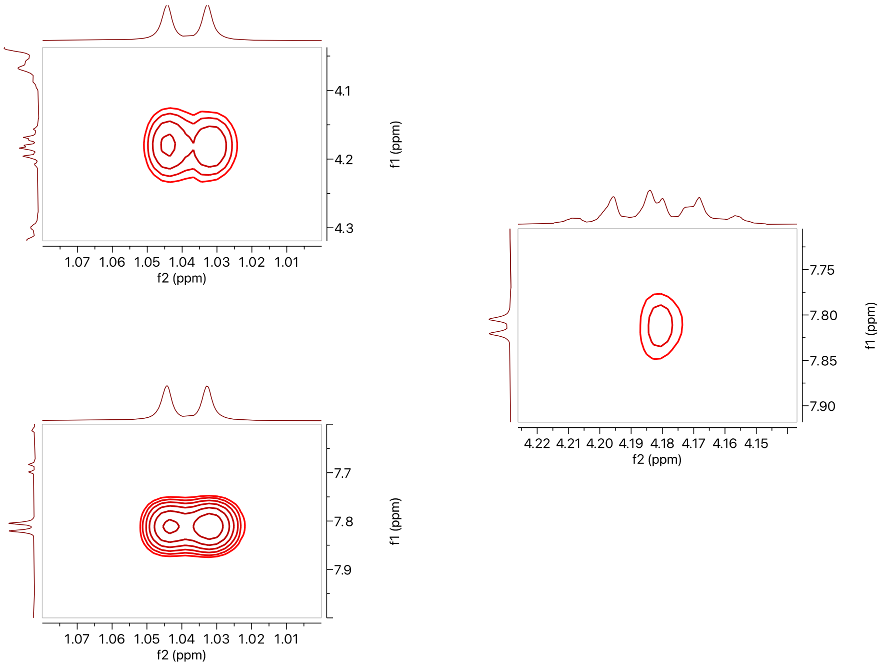

g) ^{1}H-^{13}C HMBC of the diol form of the product

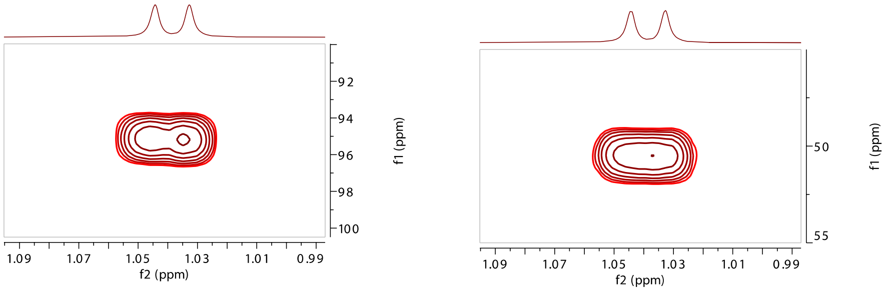

h) ^{1}H-^{1}H NOESY and ^{1}H-^{1}H ROESY cross peak between two methyl groups in the keto and diol form.

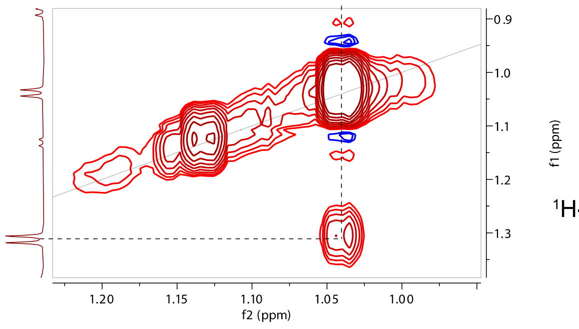

^{1}H-^{1}H NOESY

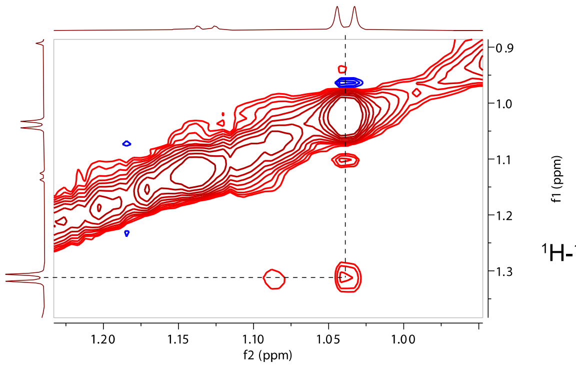

^{1}H-^{1}H ROESY

i) ^{1}H-^{13}C HSQC assignment of the aromatic region.

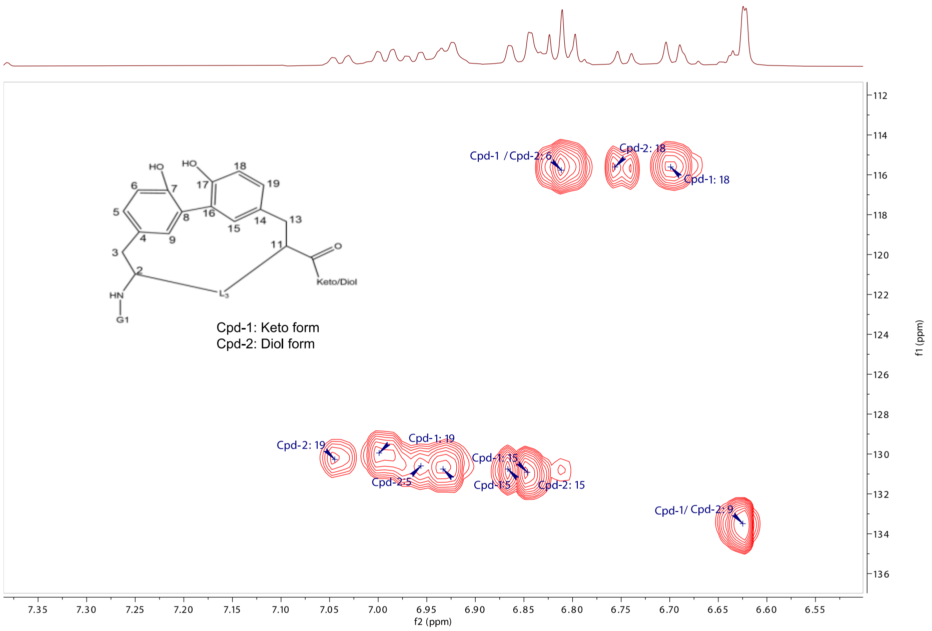

k) ^{1}H-^{13}C HMBC assignment of the aromatic region.

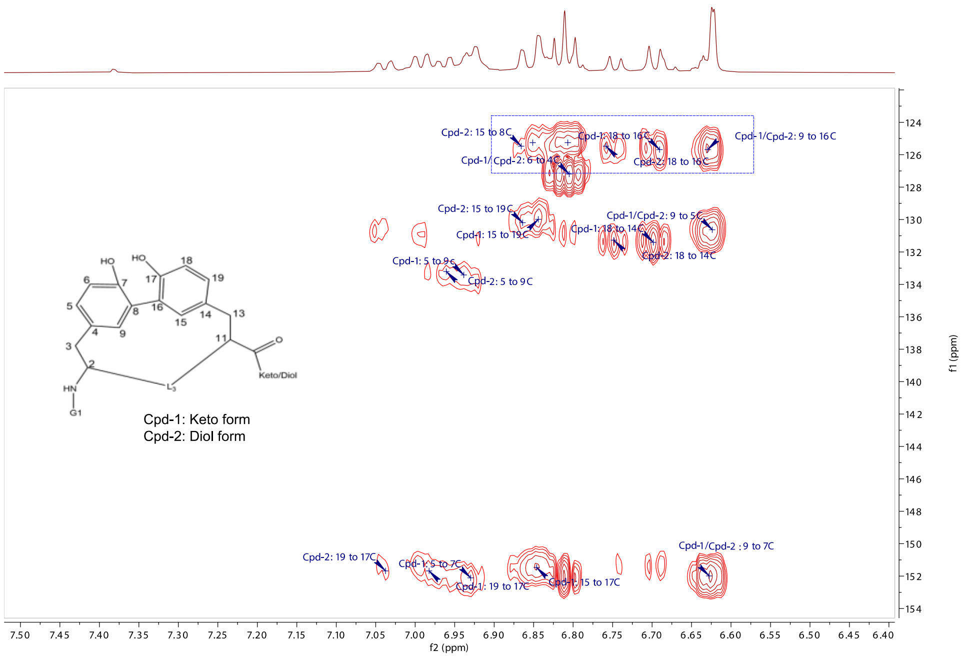

Table S7: Summary of ^{1}H and ^{13}C chemical shifts of every residue in BthAOHIS-modified product after GluC cleavage.

a) Keto form

number	AA	NH/C=O	αH/C	βH/C	γH/C	δH/C and others
1	G	166.4	4.06, 3.79<br>40.9			
2	Y	8.43<br>(br)<br>170.5	4.91<br>53.7	3.04, 2.95<br>37.1	127.4	6.93(dd), 130.9; 6.81 (d), 115.7<br>6.62 (d, 2Hz), 133.9<br>C-OH: 152.2, Ar-C (linker): 125.3
3	L	8.52<br>174.2	4.75<br>52.3	1.47<br>41.5	1.41<br>24.3	0.75, 21.7<br>0.70, 24.5
4	Y	8.99<br>171.9	4.31<br>54.7	2.54, 2.10<br>38.0	130.9	7.00 (dd), 130.2, 6.70 (d), 115.7<br>6.84 (d, 2Hz), 131.2<br>OH-C:151.4; Ar-C (linker): 125.6
5	D	8.35	4.90<br>51.6	1.31<br>14.6	202.5	171.9

b) Diol form

number	AA	NH/C=O	αH/C	βH/C	γH/C	δH/C and others
1	G	166.4	4.06, 3.79<br>40.9			
2	Y	8.21<br>(br)<br>170.5	4.84<br>54.0	3.04, 2.96<br>37.1	127.4	6.96 (dd), 130.8; 6.81 (d), 115.7<br>6.62 (d, 2Hz), 133.9<br>OH-C: 152.1, Ar-C (linker): 125.2
3	L	8.54<br>174.2	4.68<br>52.1	1.40<br>41.7	1.45<br>24.5	0.79, 21.8, 0.74, 21.5
4	Y	8.92<br>172.2	4.34<br>55.1	2.60, 2.39<br>37.4	130.7	7.04 (dd), 130.4; 6.74 (d), 115.7<br>6.87 (d, 2Hz), 130.9<br>OH-C: 151.5; Ar- C (linker): 125.6
5	D	7.81	4.18<br>50.5	1.04<br>13.9	95.3	

Figure S24: AlphaFold model of BthS (pink) superimposed with the crystal structure of MppJ (green, PDB: 4KIB). The active site of MppJ contains *S*-adenosyl-L-methionine, phenylpyruvic acid, and ferric ion. Parameters generated from DALI: Z-score = 25.7, RMSD = 3.6, number of aligned C-alpha atoms = 309, number of residues in target structure = 340. UCSF Chimera was used to generate these images.

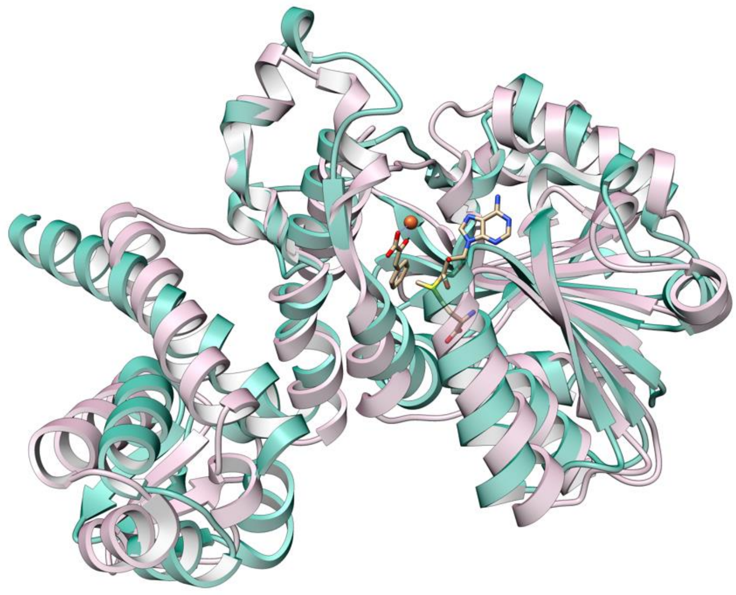

Figure S25. Proposed mechanism for the transformation catalyzed by BthHI. The BthH protein likely has two or three iron ions in its active site,[9-11] but only one is drawn. The others may aid in binding the substrate. The decarboxylation may or may not take place in the enzyme active site.

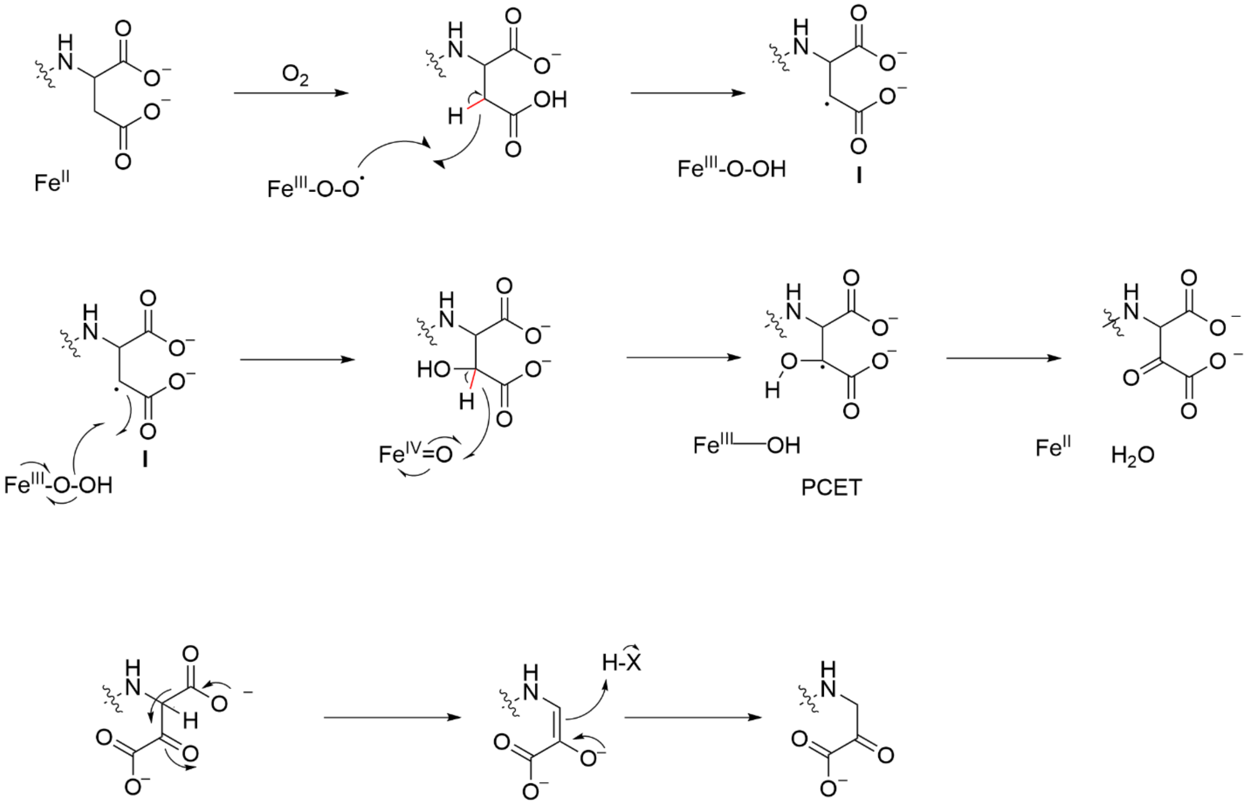

Supplemental References

(1) Yu, Y.; van der Donk, W. A. Biosynthesis of 3-thia-α-amino acids on a carrier peptide. *Proc. Natl. Acad. Sci. U. S. A.* **2022**, *119*, e2205285119.
(2) Jiang, M.; Hong, K.; Mao, Y.; Ma, H.; Chen, T.; Wang, Z. Natural 5-aminolevulinic acid: sources, biosynthesis, detection and applications. *Front. Bioeng. Biotechnol.* **2022**, *10*, doi.org/10.3389/fbioe.2022.841443.
(3) Cardona, S. T.; Valvano, M. A. An expression vector containing a rhamnose-inducible promoter provides tightly regulated gene expression in Burkholderia cenocepacia. *Plasmid* **2005**, *54*, 219-228.
(4) Brademan, D. R.; Riley, N. M.; Kwiecien, N. W.; Coon, J. J. Interactive peptide spectral annotator: A versatile web-based tool for proteomic applications. *Mol. Cell. Proteom.* **2019**, *18*, S193-S201.
(5) Niedermeyer, T. H.; Strohalm, M. mMass as a software tool for the annotation of cyclic peptide tandem mass spectra. *PLoS One* **2012**, *7*, e44913.
(6) Youran, L.; Shuyun, X.; Wilfred van der, D. A facile method for determining lanthipeptide stereochemistry. *bioRxiv* **2023**, 2023.2010.2026.564210.
(7) Livnat, I.; Tai, H.-C.; Jansson, E. T.; Bai, L.; Romanova, E. V.; Chen, T.-t.; Yu, K.; Chen, S.-a.; Zhang, Y.; Wang, Z.-y.; Liu, D.-d.; Weiss, K. R.; Jing, J.; Sweedler, J. V. A D-amino acid-containing neuropeptide discovery funnel. *Anal. Chem.* **2016**, *88*, 11868-11876.
(8) Tai, H. C.; Checco, J. W.; Sweedler, J. V. Non-targeted Identification of D-amino acid-containing peptides through enzymatic screening, chiral amino acid analysis, and LC-MS. *Methods Mol. Biol.* **2018**, *1719*, 107-118.
(9) Dou, C.; Long, Z.; Li, S.; Zhou, D.; Jin, Y.; Zhang, L.; Zhang, X.; Zheng, Y.; Li, L.; Zhu, X.; Liu, Z.; He, S.; Yan, W.; Yang, L.; Xiong, J.; Fu, X.; Qi, S.; Ren, H.; Chen, S.; Dai, L.; Wang, B.; Cheng, W. Crystal structure and catalytic mechanism of the MbnBC holoenzyme required for methanobactin biosynthesis. *Cell Res.* **2022**, *32*, 302-314.
(10) Park, Y. J.; Jodts, R. J.; Slater, J. W.; Reyes, R. M.; Winton, V. J.; Montaser, R. A.; Thomas, P. M.; Dowdle, W. B.; Ruiz, A.; Kelleher, N. L.; Bollinger, J. M., Jr.; Krebs, C.; Hoffman, B. M.; Rosenzweig, A. C. A mixed-valent Fe(II)Fe(III) species converts cysteine to an oxazolone/thioamide pair in methanobactin biosynthesis. *Proc. Natl. Acad. Sci. U. S. A.* **2022**, *119*, e2123566119.
(11) Zheng, Y.; Xu, X.; Fu, X.; Zhou, X.; Dou, C.; Yu, Y.; Yan, W.; Yang, J.; Xiao, M.; van der Donk, W. A.; Zhu, X.; Cheng, W. Structures of the holoenzyme TglHI required for 3-thiaglutamate biosynthesis. *Structure* **2023**, *31*, 1220-1232.e1225.
